# Ancient River Inference Explains Exceptional Oriental Freshwater Mussel Radiations

**DOI:** 10.1038/s41598-017-02312-z

**Published:** 2017-05-18

**Authors:** Ivan N. Bolotov, Alexander V. Kondakov, Ilya V. Vikhrev, Olga V. Aksenova, Yulia V. Bespalaya, Mikhail Yu. Gofarov, Yulia S. Kolosova, Ekaterina S. Konopleva, Vitaly M. Spitsyn, Kitti Tanmuangpak, Sakboworn Tumpeesuwan

**Affiliations:** 1 Department of Science, Northern Arctic Federal University, Arkhangelsk, Russian Federation; 2 Department of Science, Faculty of Science and Technology, Loei Rajabhat University, Loei, Thailand; 3 Department of Biology, Faculty of Science, Mahasarakham University, Maha Sarakham, Thailand

## Abstract

The concept of long-lived (ancient) lakes has had a great influence on the development of evolutionary biogeography. According to this insight, a number of lakes on Earth have existed for several million years (e.g., Baikal and Tanganyika) and represent unique evolutionary hotspots with multiple intra-basin radiations. In contrast, rivers are usually considered to be variable systems, and the possibility of their long-term existence during geological epochs has never been tested. In this study, we reconstruct the history of freshwater basin interactions across continents based on the multi-locus fossil-calibrated phylogeny of freshwater mussels (Unionidae). These mussels most likely originated in Southeast and East Asia in the Jurassic, with the earliest expansions into North America and Africa (since the mid-Cretaceous) following the colonization of Europe and India (since the Paleocene). We discovered two ancient monophyletic mussel radiations (mean age ~51–55 Ma) within the paleo-Mekong catchment (i.e., the Mekong, Siam, and Malacca Straits paleo-river drainage basins). Our findings reveal that the Mekong may be considered a long-lived river that has existed throughout the entire Cenozoic epoch.

## Introduction

The concept of long-lived (ancient) lakes is one of the most important scientific breakthroughs in evolutionary biogeography^1–3^. According to this concept, a few lakes on Earth (e.g., Baikal, Tanganyika, Malawi, and Titicaca) are considered to have existed for several million years. These lakes have different origins and environmental characteristics, but all of them are well-known evolutionary hotspots with exceptionally high biodiversity and levels of endemism^3–7^. The lakes should therefore be considered great ‘natural laboratories’ to study the general patterns of species diversification, adaptive radiation and convergent evolution. One of the main diagnostic features of ancient lakes is the presence of sister species flocks, which have evolved through intra-lake radiations^3, 8–11^.

Rivers are usually considered highly dynamic systems undergoing rapid environmental changes^12–14^. To the best of our knowledge, the possibility of the stable existence of river systems during long-term periods comparable with geological epochs has never been tested. A number of scholars suggest that certain endemic species may be considered as ancient relicts surviving for several millions of years in a fluvial system^15, 16^. Riverine radiations have not attracted the same attention as speciation in lakes, and there are only certain known examples, particularly, among gastropods^17–21^. De Bruyn *et al*. show that paleo-drainage re-arrangements resulting from Quaternary climate change played a significant role in the spatiotemporal evolution of halfbeak fishes (Zenarchopteridae) across mainland Southeast Asia and the Greater Sunda Islands^22^. The radiation of these fish taxa was largely associated with gigantic paleo-river systems of the former Sundaland (i.e., the Mekong, Malacca Straits, Siam, North Sunda, and East Sunda paleo-river drainage basins). Wesselingh notes that unionoid radiations are known from North American and Chinese river systems and suggests that their high species richness should reflect a long basin history with drainage reorganizations and plenty of relatively isolated rivers, where speciation can occur^23^.

We use freshwater mussels in the family Unionidae, or naiads, as a model system for our biogeographic reconstruction. These mussels have been considered the most species-rich bivalve family, with ~620–680 extant species^24–27^. The mussels can disperse in the larval stage together with host fishes, and their native spreading is usually associated with direct connections between freshwater basins, e.g., during low sea levels resulting in the formation of gigantic freshwater drainages, which join river systems of continental margins and islands by the drying shelf ^22, 28–31^. Interestingly, naiads are comparatively rare elements of ancient lake faunas, e.g., with 2–4 endemic species living sympatrically in the East African lakes^23, 32, 33^. The biogeographic history of naiads has been the subject of numerous studies that are based on classical biogeographic approaches^34, 35^. In contrast, the global reconstruction of the naiad biogeography based on a phylogenetic approach are still lacking, although such work for another mussel family, Margaritiferidae, has recently been published^36^. Lopes-Lima *et al*. revised the taxonomy of the Unionidae, separated the primary monophyletic clades and mapped the distribution range of each clade^27^. However, the evolutionary biogeographic patterns of unionids are almost unknown, with only a few reports published^37^. The Oriental naiads are poorly studied by means of a molecular approach^27, 37–42^. The morphology-based taxonomic concept suggests that there are many widespread species, which ranged across a plethora of river systems from the Greater Sunda Archipelago and the Malay Peninsula to Indo-China and India^42–45^, although recent molecular studies reveal possible cryptic taxa^41^.

Our discovery of the ancient age of the primary clades of the Unionidae, the species-rich bivalve family, identified using the most comprehensive multi-locus fossil-calibrated phylogeny together with wide-scale biogeographic modelling, provides surprising new insight into the early evolutionary history of freshwater basins across continents. The origin of naiads is confidently placed within Southeast and East Asia, which underscores a new crucial biogeographic pattern because naiads are keystone species of wide range freshwater ecosystems in Eurasia, North America, and Africa, as well as on a variety of islands. The Indo-Chinese naiad radiations are the first molecular evidence of the long-term intra-basin evolution of naiads, which inhabited the putative gigantic paleo-Mekong catchment (i.e., the Mekong, Siam, and Malacca Straits paleo-river drainage basins) during approximately 50 Ma. These findings provide new evidence for understanding the origin and dispersal routes of freshwater fauna around the world. Additionally, we show that each large freshwater basin of Indo-China is a separate evolutionary hotspot harboring a unique endemic naiad fauna and should therefore be a special focus for conservation efforts.

## Results

### Comprehensive phylogeny of the Unionidae

**Figure 1:**
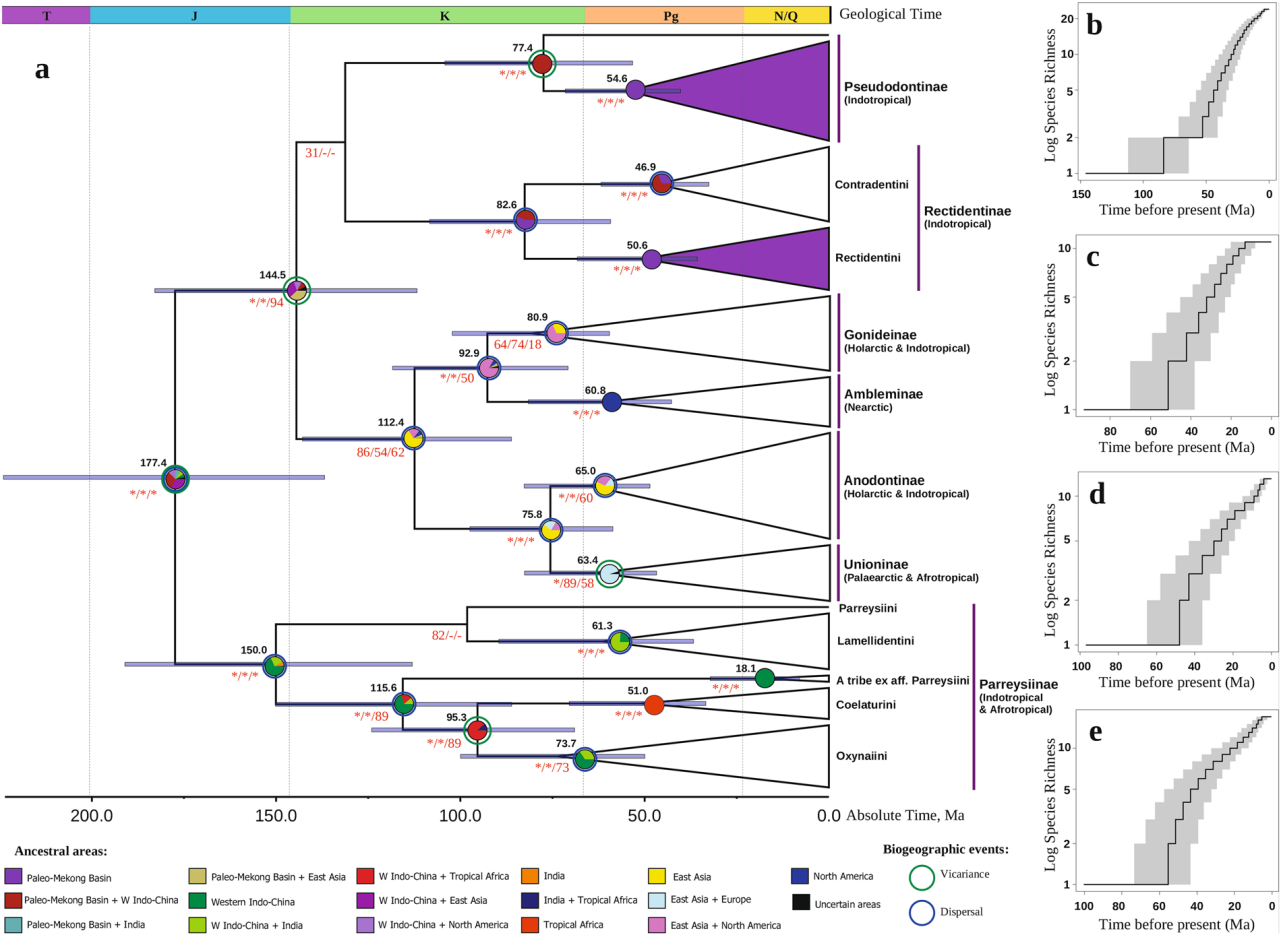
Multi-locus fossil-calibrated phylogeny, ancestral area reconstruction and diversification of the Unionidae. (**a**) Biogeography and divergence times of the primary clades of the Unionidae inferred from statistical analyses based on the fossil-calibrated ultrametric chronogram calculated under a lognormal relaxed clock model and a Yule process speciation implemented in BEAST 1.8.3 and obtained for the complete data set of mitochondrial and nuclear sequences (five partitions: three codons of COI + 16S rRNA + 28S rDNA). Pie chaps near nodes indicate the probabilities of certain ancestral areas with respect to combined results under three different modeling approaches (S-DIVA, DEC and S-DEC). The violet filling of collapsed nodes indicates the two ancient bivalve radiations within the Paleo-Mekong drainage basin. Black numbers near nodes are the mean age values, and bars are 95% confidence intervals of the estimated divergence time between lineages (Ma). Red numbers near nodes are BPP values inferred from BEAST, BPP values inferred from MrBayes and BS values inferred from RAxML (an asterisk indicates BPP and BS values of ≥ 95%; and “-” indicates a topological difference). The ancestral area reconstruction and timing of doubtful nodes, the position of which differs in different analyses, are omitted. Stratigraphic chart according to the International Commission on Stratigraphy, 2015. The full variants of the chronogram and ancestral area reconstructions are presented in Supplementary Figs 4 and 5. The list of sequences is presented in Supplementary Table 1. (**b**–**e**) Semilogarithmic lineage-through-time (LTT) median plots of chronograms estimated from 36,004 post-burn-in Bayesian trees for several endemic Indo-Chinese clades, including (**b**) Rectidentinae, (**c**) Rectidentini (a paleo-Mekong clade), (**d**) Contradentini, and (**e**) Pseudodontinae (a paleo-Mekong clade). The gray filling indicates 95% confidence intervals.

**Figure 2:**
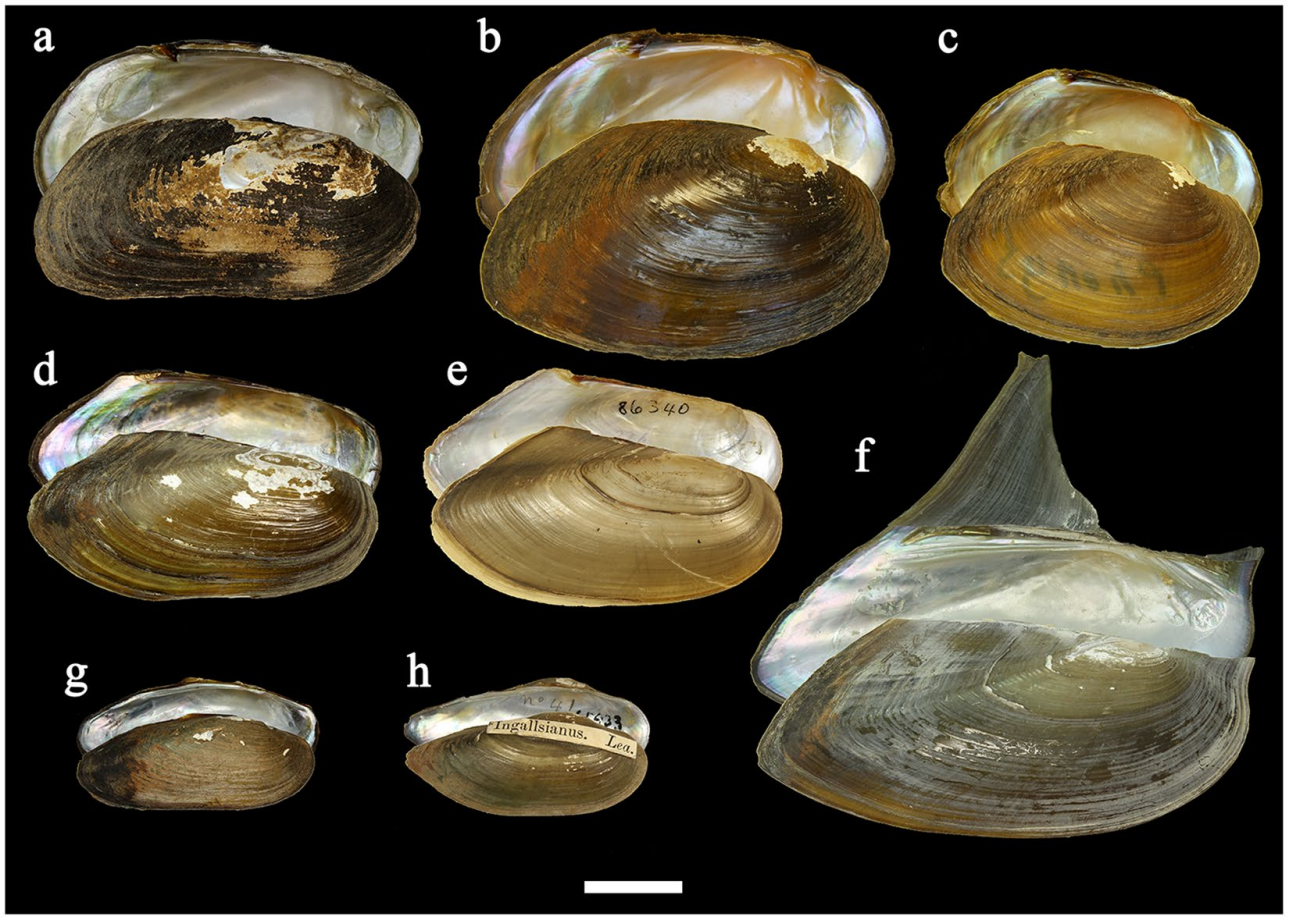
Shell shape variability within the two ancient radiations from the paleo-Mekong basin. (**a**–**e**) Pseudodontinae, including (**a**) *Pseudodon* aff. *mouhotii* sp.1, Nam Long River, Laos (RMBH biv182/19), (**b**) *P*. aff. *vondembushianus* sp.1, Phong River, Thailand (RMBH biv122/5), (**c**) *P*. cf. *ellipticus*, Phong River, Thailand (RMBH biv120/3), (**d**) *Pilsbryoconcha* cf. *compressa*, artificial pond near Ban Nhong Buen village, Thailand (RMBH biv116), (**e**) *P. exilis*, Thailand (USNHM 86340). (**f**–**h**) Rectidentini, including (**f**) *Hyriopsis sp.2*, Chi River, Thailand (RMBH biv130), (**g**) *Ensidens* aff. *sagittarius* sp.2, artificial pond near Ban Nhong Buen village, Thailand (RMBH biv117), and (**h**) *E. ingallsianus*, Thailand (USNHM 85933). Scale bar = 2 cm (Photos: Ilya V. Vikhrev).

The resulting alignment contains 1,848 bp-long sequences for 337 in-group mussel specimens with three gene fragments: 659 bps of mitochondrial *cytochrome c oxidase I* (COI) and 434 bps of 16S rRNA, in addition to 755 bps of nuclear 28S rDNA (Supplementary Table 1). The highly resolved and well-supported multi-locus phylogeny of the Unionidae obtained from this combined data set includes 122 species from across the entire range of the family: the Oriental Region (Indo-China, Malay Peninsula and India), East Asia, Europe, Africa and North America (Fig. 1a). Searches with BEAST, MrBayes and RAxML all resulted in congruent topologies for in-group taxa (Supplementary Figs 1–4 and Supplementary Table 2). We found seven primary clades within the family, which appear to be a subfamily level: Anodontinae, Unioninae, Gonideinae, Ambleminae, Rectidentinae, Pseudodontinae and Parreysiinae. Two subfamilies, Rectidentinae and Pseudodontinae, exclusively range in Indo-China and surrounding areas. Among them, we recorded two well-supported monophyletic clades, the range of which is restricted within the Mekong and Chao Phraya catchments and rivers of the Malay Peninsula, i.e., the paleo-Mekong basin, which likely joined the Mekong, Siam, and Malacca Straits paleo-river systems (Fig. 2). First, it is a large clade within Pseudodontinae, which includes all of our sequenced representatives of the subfamily, excluding only a highly divergent lineage from the Irrawaddy catchment. Second, it is the entire tribe of Rectidentini within the Rectidentinae.

### Origin and ancient radiations of the Unionidae

**Figure 3:**
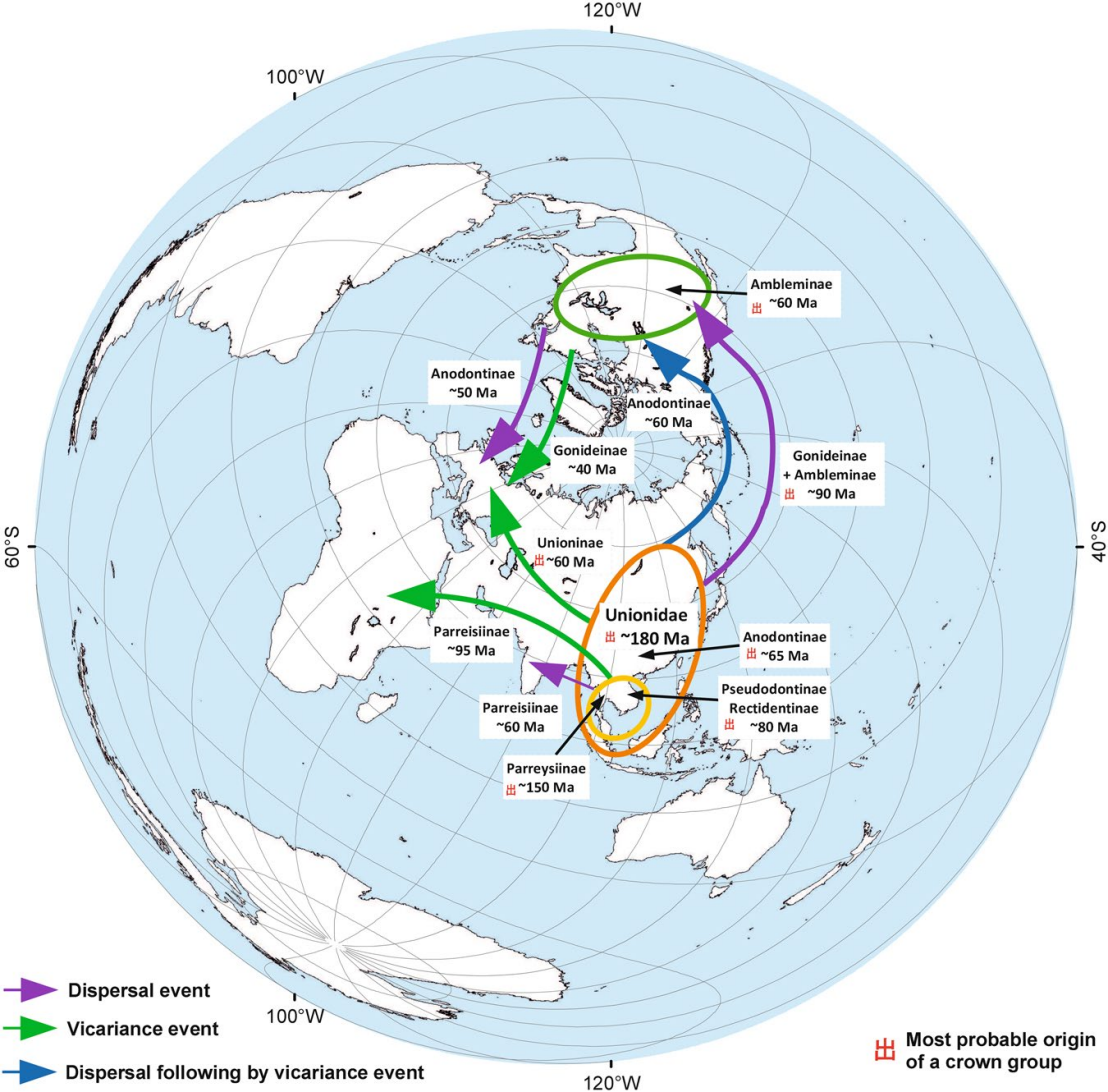
Simplified scheme of origin and expansion routes inferred across clades of the Unionidae. The black numbers near arrows show the timing of putative expansion events (mean age) obtained from the multi-locus fossil-calibrated phylogenetic model (see Fig. 1 and Supplementary Figs 4 and 5 for details). Ellipses indicate the putative places of origin of the family (orange) and several subfamilies (yellow and green). The map was created using ESRI ArcGIS 10 software (www.esri.com/arcgis); the topographic base of the map was created with ESRI Data and Maps.

The combined results of our worldwide biogeographic modelling (S-DIVA, DEC and S-DEC approaches) based on the fossil-calibrated chronogram inferred from the relaxed molecular clock analyses returned a robust ancestral area reconstruction for the primary clades of the Unionidae (Figs 1a and 3, Supplementary Fig. 5). This scenario suggests that the Unionidae MRCA originated somewhere within East and Southeast Asia (probability 33.1% for East Asia + western Indo-China and 29.3% for the paleo-Mekong basin + western Indo-China). The S-DEC model supports the same scenario (probability 33.6% for East Asia + western Indo-China and 33.4% for the paleo-Mekong basin + western Indo-China), whereas the DEC model highlights the possible primary role of East Asia and western Indo-China (probability 51.1%). The S-DIVA model assumes that the Unionidae MRCA most likely originated within Indo-China (probability 46.6% for the paleo-Mekong basin + western Indo-China). The origin of the crown group of the family was placed in the Jurassic (mean age 177 Ma, 95% HPD 137–224 Ma). Based on the combined biogeographic model, the Parreysiinae MRCA most likely originated in western Indo-China (probability 68.3%), and the MRCA of all other clades most likely evolved in East and Southeast Asia (probability 37.9% for East Asia + western Indo-China and 31.7% for East Asia + the paleo-Mekong basin). The crown groups of these two most ancient unionid clades originated almost simultaneously in the Late Jurassic (mean age ~150 Ma, 95% HPD 112–191 Ma).

The crown groups of Rectidentinae and Pseudodontinae, autochthonous Indo-Chinese subfamilies (probability 100%), simultaneously originated in the Late Cretaceous (mean age ~80 Ma, 95% HPD 53–108 Ma). The combined scenario suggests that two possible endemic clades of the paleo-Mekong catchment, Rectidentini and the corresponding Pseudodontinae subclade, likely evolved through intra-area radiation (probability 100% for both clades). Their origin is placed in the Eocene (mean age 51–55 Ma, 95% HPD 36–71 Ma). The MRCA of Anodontinae, Unioninae, Gonideinae and Ambleminae likely originated in East Asia (probability 65.8%). The East Asian origin is also suggested for the Anodontinae + Unioninae MRCA (probability 60.9%) and for the Anodontinae MRCA (probability 56.7%). In contrast, the Unioninae MRCA likely ranged across Eurasia (probability 95.3%), and those of Gonideinae + Ambleminae and Gonideinae were likely continuously distributed in East Asia and North America (probability 83.1% and 68.2%, respectively). Additionally, North America is the most likely place of origin of the Ambleminae (probability 100%). The dating of origin for almost all these clades is similar and placed near the Cretaceous/Paleocene boundary (mean age 61–65 Ma, 95% HPD 43–83 Ma), excluding that for the Gonideinae, which appears to be slightly more ancient (mean age ~80 Ma, 95% HPD 60–102 Ma).

With respect to our combined biogeographic scenario, Coelaturini, an African tribe, appears to be a descendant of a widespread clade of Parreysiinae, representatives of which spread from western Indo-China into Africa in the mid-Cretaceous (probability 88.2%; mean age 95 Ma, 95% HPD 69–124 Ma). Another descendant of this MRCA is Oxynaiini, a clade of most likely western Indo-Chinese origin (probability 65.6%), with recent species also distributed in India. These two clades diverged via a vicariance event (probability 57.8%). The Lamellidentini MRCA appears to be continuously ranged in western Indo-China and India near the Cretaceous/Paleocene boundary (probability 75.8%; mean age 61 Ma, 95% HPD 37–90 Ma). The western Palearctic taxa of Anodontinae and Gonideinae (e.g., well-known species such as *Anodonta anatina*, *A. cygnea*, *Microcondylaea compressa*, and others) originated through secondary spreading from North America to Europe, likely via the North Atlantic land connection (probability 66.2 and 100%, respectively) (Fig. 3). These events are placed in the Eocene (mean age 47 Ma, 95% HPD 34–62 Ma and mean age 38 Ma, 95% HPD 23–55 Ma for Anodontinae and Gonideinae, respectively). In contrast, common European members of the Unioninae (e.g., *Unio crassus*, *U. pictorum*, *U. tumidus*, and others) are descendants of an MRCA with a broad trans-Eurasian range (probability 95.3%), which has been separated via a vicariance event (probability 95.3%).

### Diversification rates in autochthonous Indo-Chinese clades

Our lineage-through-time modelling suggests slow diversification rates in the Rectidentinae and Pseudodontinae (Fig. 1b–e). The constant-rate test suggests that almost all clades diversified under the pure-birth (constant) model, with the exception of Rectidentini, which has a significantly decreasing rate of diversification through time (Supplementary Table 3). Paradis’s test of diversification with three survival models returns a declining diversification rate in Rectidentinae as a whole, Rectidentini and the Mekong’s Pseudodontinae clade, but not Contradentini (Supplementary Table 3).

### Spatial patterns of the naiad biodiversity across the Oriental Region

**Figure 4:**
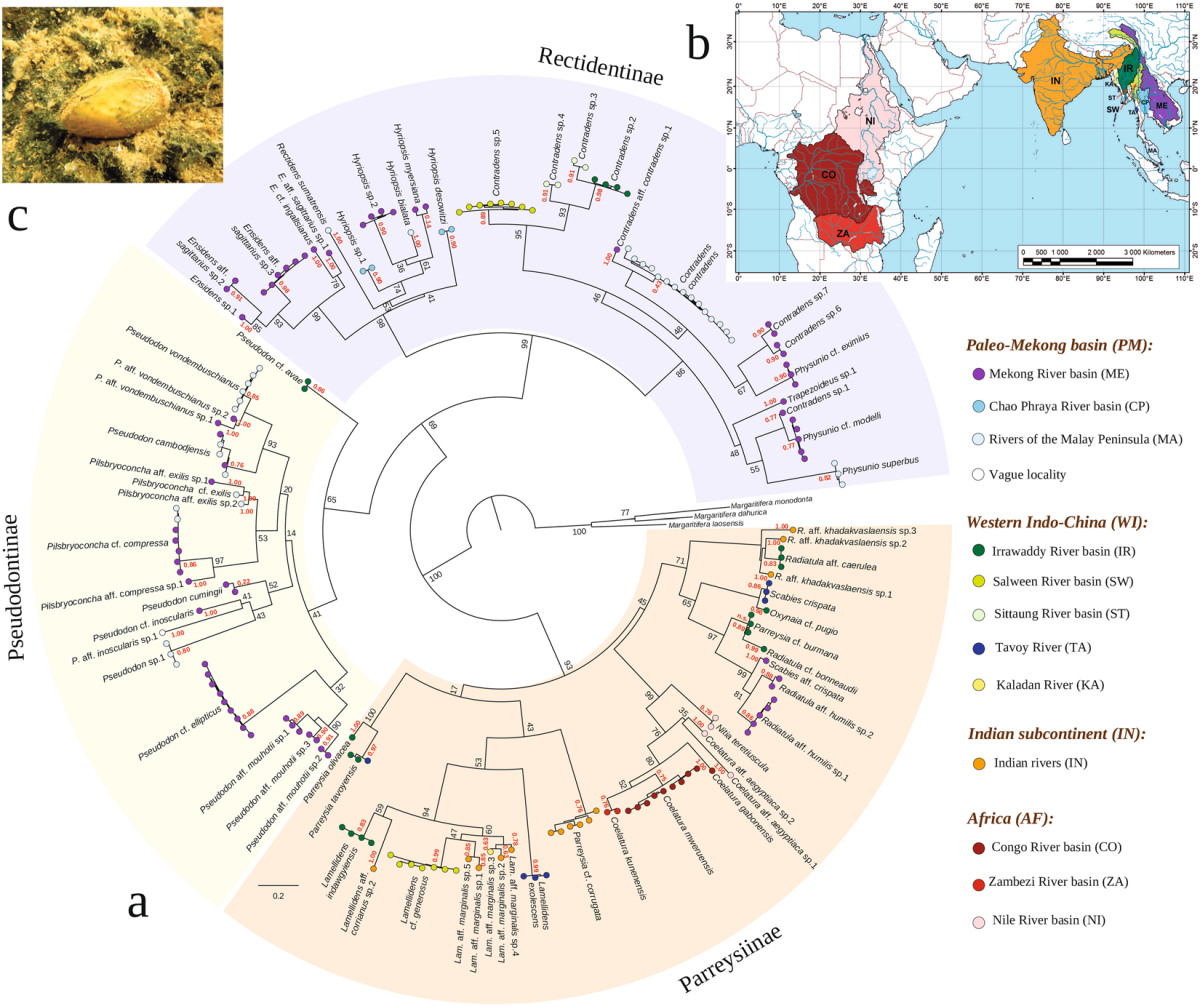
Spatial patterns in the biodiversity of the Unionidae from the Oriental Region and related African taxa discussed in this study. (**a**) Maximum likelihood tree of unique haplotypes obtained from RAxML HPC Black Box v. 8.2.9. Color circles correspond to the distribution range of each haplotype. Red numbers near terminal nodes are support values for each prospective species (MOTU) based on the Bayesian PTP model. Black numbers near other nodes are ML support values. The list of sequences is presented in Supplementary Table 1. (**b**) Map of the primary distribution areas of the Unionidae. The map was created using ESRI ArcGIS 10 software (www.esri.com/arcgis); the topographic base of the map was created with Natural Earth Free Vector and Raster Map Data (www.naturalearthdata.com). (**c**) Live specimen of *Parreysia* cf. *burmana*, Irrawaddy River catchment, Myanmar (Photo: Ivan N. Bolotov).

Our data set contains 271 specimens of naiads from the Oriental Region, including those from the Mekong, Salween, Sittaung and Irrawaddy catchments, Indian rivers and some small river basins in Indo-China and the Malay Peninsula (Supplementary Tables 1 and 4). Additionally, 16 specimens of related African forms were included in the analysis. This sample contains 192 unique COI haplotypes. The delimitation of these haplotypes using the Poisson Tree Processes (PTP) model with the supplement of the analyses of the 16S rRNA and 28S rDNA gene sequences revealed 72 Molecular Operational Taxonomic Units (MOTUs) corresponding to prospective biological species, including 64 species from the Oriental Region and 6 species from Africa (Fig. 4). Approximately half of these species represent cryptic taxa (40 species, 56% of a total sample), which were not clearly assigned to valid species, and several of which may be new to science. No haplotypes distributed across two or more separate freshwater basins were discovered. Moreover, no widespread species ranging across a variety of the Oriental basins have been recorded. The distribution range of the majority of species in our data set was situated within a certain freshwater drainage, excluding those of three species: *Pilsbryoconcha* cf. *compressa*, *Pseudodon cambojensis* and *Parreysia tavoyensis* (Fig. 4 and Supplementary Table 5). Extremely low similarity between unionid faunas from each catchment reflects such a drainage-shaped distribution pattern (Supplementary Tables 5 and 6).

## Discussion

### Origin and early diversification of the Unionidae

In this study, we present results of the global phylogenetic and biogeographic modelling based on the most comprehensive multi-locus data set for the Unionidae to date (337 representatives of 122 species) with a special focus on the Indo-Chinese fauna. In general, our phylogenetic analyses support the taxonomy of Lopes-Lima *et al*.^27^, but expanded sampling of the Oriental taxa shows that the placement of the Pseudodontinae members as a tribe within Gonideinae was incorrect, and they actually represent a separate subfamily. This clade is robust and well supported in almost all variants of phylogenetic analyses, including those for separate gene partitions (Supplementary Table 2). Together with the Modellnaiinae, which contains a single monotypic genus^27, 43^, there are three endemic Southeast Asian subfamilies, almost half of those currently known within the Unionidae. The Parreysiinae, a very ancient clade, may actually represent several subfamily-level taxa, but the relationships of these subclades are still unresolved and should be clarified in the future based on an extended sampling from Africa and the Oriental Region.

In accordance with our fossil-calibrated phylogenetic hypothesis, the origin of the crown group of the Unionidae is placed in the Early Jurassic (mean age ~180 Ma), which corresponds well to a wide-scale time-calibrated phylogeny obtained from all bivalve taxa^46^. Our present model suggests that the crown groups of two other freshwater mussel families, Hyriidae and Margaritiferidae, are 1.3–2.6 times younger compared with the Unionidae, with mean ages of 135 and 70 Ma, respectively (Supplementary Fig. 4). These dates are smaller than those inferred from previous models assuming mean ages of ~190 and 90 Ma for Hyriidae and Margaritiferidae, respectively^36, 47^, because we used more flexible stem priors for the majority of fossil calibrations (see Methods section). A possible younger age of the crown groups of the Hyriidae and Margaritiferidae compared with the Unionidae may partly explain lower species richness in both clades^48^ (Supplementary Fig. 6), although extremely low diversity in extant margaritiferids may also reflect their narrow ecological specialization^49^ and slow evolutionary rates^36^.

We provide the first molecular evidence that the origin and early diversification of the family were within East and Southeast Asia, which is well in agreement with paleontological records^50–53^. At first glance, the mean age of the Unionidae coincides with the initial stage of the break-up of Pangea ~180 Ma ago^54^. With respect to our hypothesis, main expansion events that have largely shaped the broad recent distribution of the family occurred during the period from the mid-Cretaceous to the mid-Paleogene (see Fig. 3). The earliest expansions were into North America and Africa (since the mid-Cretaceous) following colonization of Europe and India (since the Paleocene).

Traditionally, the presence of unionids in tropical Africa and India has attracted the full attention of scientists as an enigmatic biogeographic phenomenon, which may be explained by the transferring of Gondwanan forms to Asia on the Indian Plate. Examples of such forms are caecilians^55^, frogs^56, 57^ and freshwater crabs^58^. The origin of the African Coelaturini clade and its sister relationships with Oriental taxa have been under long-term discussions in light of two primary hypotheses: “Into Africa” and “Out of Africa”^32, 37, 59, 60^. With respect to the “Into Africa” hypothesis, the presence of the Unionidae in the Indian subcontinent and Africa can be explained by an invasion of the same Asiatic lineage when these continents contacted Eurasia during the Eocene (India) and Miocene (Africa)^37, 59, 60^. By contrast, the “Out of Africa” hypothesis predicts that the Unionidae arose on Gondwana, populating the northern continents from the south^37, 59, 60^. Our modelling returns the scenario that the MRCA of Parreysiinae originated in western Indo-China, with subsequent dispersal of a descendant into Africa, supporting the first hypothesis, but our dating suggests the mid-Cretaceous age of this spreading. With respect to these data, *Germainaia geayi*, a unique unionid-like mussel species from Madagascar^59, 60^, may also be a descendant of this ancient dispersal event. Currently, this species is placed within Parreysiinae based solely on morphological features because viable populations of this enigmatic taxon are still unknown^27^.

As for the clades, representatives of which ranged in India, our modelling suggests that they have clear affinities with western Indo-China, similar to those for related African forms. The MRCA of Lamellidentini has likely ranged in Indo-China and India, and its mean age (61 Ma) roughly corresponds to the time of presence of Asian forms in India during the Late Cretaceous, 65–70 Ma^61, 62^. Several geological models predict multiple ancient land connections between India and Asia, but their direct biogeographic connectivity remains largely unexplored^61^. In summary, we have suggested that unionids colonized India from western Indo-China via possible ancient connections between these landmasses. This pattern is reflected by certain other taxa, e.g., pachychilid freshwater gastropods (spreading from Southeast Asia to India) and freshwater crabs (vice versa)^58, 63^. Based on these data, faunal exchanges between Asia and India were sometime during the Eocene, 56–34 Ma ago^58, 63^.

### The concept of long-lived (ancient) rivers

The discovery of the two ancient monophyletic radiations of naiads (mean age 51–55 Ma), which were likely evolved within the paleo-Mekong catchment, is of exceptional interest. To our knowledge, they represent the first intra-basin freshwater bivalve radiations with relatively high richness of extant species (17 and 12 species in a Pseudodontinae subclade and Rectidentini, respectively). These findings suggest that the Mekong may be considered a long-lived river existing thorough the Cenozoic epoch, an exceptional evolutionary hotspot of freshwater biodiversity, which has worldwide significance. This hypothesis is the first attempt to explain the radiation of freshwater taxa in a river system by means of the concept of long-lived ecosystems and appears to be a novel theoretical basis for further biogeographic studies with a special focus on the world’s largest rivers (e.g., Mekong, Yangze, Mississippi, Amazon, Orinoco, and others). Our results are in agreement with the hypothesis of Schneider *et al*. that the Mekong and Yangtze unionid faunas basically must have developed in independent radiations during the entire Cenozoic^53^. The Yangtze with 68 nominal unionid taxa is considered to be the most species-rich basin in Asia, followed by the Mekong catchment, with 51 nominal species^42^. Currently the Yangtze fauna is poorly studied by means of a molecular approach, although this basin may have keystone significance in further understanding the origin and evolutionary patterns in freshwater mussels.

Moreover, our model supports the assumption of Wesselingh that a long freshwater basin history together with drainage re-arrangements and a plethora of relatively isolated tributaries are the main drivers of diversification processes in riverine unionoids^23^. Interestingly, our results reveal slowed diversification rates in the endemic Indo-Chinese clades. This finding together with a small amount of available data on slow substitution rates within Unionoida^36, 64^ suggest a possible link between delayed diversification and slow molecular evolution in freshwater mussels, but this preliminary assumption need to be checked in the future. Based on the comprehensive meta-analysis of multiple terrestrial and freshwater taxa, de Bruyn *et al*. note that within-area diversification and subsequent emigration have been the predominant signals characterizing Indo-China’s biota since at least the Early Miocene^30^. Our results fully support this finding, but the endemic Indo-Chinese naiad fauna appears to be much more ancient and likely evolved within this area at least since the Late Cretaceous.

### Distribution patterns and conservation priorities for the Oriental Unionidae

The analysis of spatial biodiversity patterns does not support previous taxonomic solutions, suggesting that many species of naiads have wide distribution ranges across a variety of freshwater basins in the Oriental Region, iconic examples of which are *Trapezoideus exolescens*, *Contradens contradens*, *Scabies crispata* and others^42–45^. Taking into account that the distribution range of the majority of species studied by us correspond to certain catchments, we suggest that widespread species of naiads are actually represent species complexes, each of which includes several cryptic species with a local range consistent with the records of Zieritz *et al*.^41, 42^. Only three species, *Pilsbryoconcha* cf. *compressa*, *Pseudodon cambojensis* and *Parreysia tavoyensis*, were recorded from separate catchments. The first two taxa likely spread via river connections within the paleo-Mekong catchment (Mekong and rivers of the Malay Peninsula) across Sundaland during the Pleistocene^22, 30, 31^, and the latter one reveals possible connections between the Irrawaddy and Tavoy drainage basins at the same time. Our findings reveal that each large river system in Indo-China differs by exceptionally high levels of endemism of the Unionidae, and may therefore represent a unique evolutionary hotspot for entire freshwater fauna. Drainage-associated spatial structure is a typical feature of freshwater mussel biodiversity in other regions, e.g., in the western North America^65^.

Finally, the Mekong, a trans-boundary “mother” river of Southeast Asia, should be considered as a high priority system for study and conservation due to its putative ancient age, the possible important role in origin of mussel faunas within other basins and an exceptional level of modern biodiversity. Further integrative studies of the Unionidae in each large river system in Southeast Asia, an actively developing tropical region with high economic growth and urbanization rates, are urgently need for effective conservation planning.

## Methods

### Taxon sampling and laboratory protocols

To reconstruct the phylogeny of the Unionidae, we sampled 337 representatives of 122 species from the entire range of the family: the Oriental Region (Indo-China and India), East Asia, Europe, Africa and North America (Supplementary Table 1). New sequences were obtained from 153 specimens belonging to 28 species that were collected from primary freshwater drainages across Indo-China, i.e., the Mekong, Salween, Sittaung and Irrawaddy catchments, as well as some smaller rivers (Supplementary Table 4). Additionally, new sequences were obtained from seven species inhabiting rivers of East Asia and Middle East (Supplementary Table 1). The total genomic DNA was isolated from ethanol-preserved tissue samples using the NucleoSpin^®^ Tissue Kit (Macherey-Nagel GmbH & Co. KG, Germany) following the manufacturer’s protocol. For molecular analyses, we obtained partial sequences of two mtDNA markers, i.e., the *cytochrome c oxidase* subunit I gene (COI) and the *16S ribosomal RNA* (16S rRNA), and a fragment of the *nuclear 28S ribosomal DNA* (28S rDNA). The list of primer sequences that were used for PCR is shown in Supplementary Table 7. Thermocycling was implemented with marker-specific PCR programs as follows: (i) COI: 95 °C (4 min), followed by 37 cycles at 94 °C (50 sec), 50 °C (50 sec), 72 °C (50 sec) and a final extension at 72 °C (5 min); (ii) 16S rRNA: 95 °C (4 min), followed by 33 cycles at 94 °C (50 sec), 47 °C (50 sec), 72 °C (50 sec) and a final extension at 72 °C (5 min); (iii) 28S rDNA: 95 °C (4 min), followed by 38 cycles at 94 °C (50 sec), 57 °C (50 sec), 72 °C (50 sec) and a final extension at 72 °C (5 min). Forward and reverse sequence reactions were performed directly on purified PCR products using the ABI PRISM^®^ BigDye™ Terminator v. 3.1 reagent kit and run on an ABI PRISM^®^ 3730 DNA Analyzer (Thermo Fisher Scientific Inc., Waltham, MA, USA). To check the resulting sequences, we used a sequence alignment editor (BioEdit v. 7.2.5). Additional sequences of 92 species (177 specimens in a total) were obtained from NCBI GenBank, including those from Indo-China (35 species, 105 specimens), India (9 species, 14 specimens), Africa (6 species, 16 specimens), East Asia (17 species, 17 specimens), Europe (9 species, 9 specimens), and North America (16 species, 16 specimens). This sample includes all primary clades of the Unionidae, which were determined in the recent study^27^. As an out-group, 25 mussel species were sampled, including representatives of Margaritiferidae (10 species), Iridinidae (2 species), Etheriidae (1 species), Mycetopodidae (1 species), Hyriidae (9 species) and Trigoniidae (2 species) (Supplementary Table 1).

### Sequence alignment, species delimitation and phylogenetic analyses

The sequence alignment was performed for each gene separately using the Muscle algorithm implemented in MEGA6^66^. The aligned sequence data sets were checked with GBlocks v. 0.91b to exclude hypervariable fragments from the sequence alignments using options for less stringent selection, which enabled gap positions, smaller final blocks and less strict flanking positions^67^. The resulting lengths of the sequence alignments are listed in Supplementary Table 8. To estimate each partition, namely, the COI, 16S rRNA, and 28S rDNA, for evidence of substitution saturation, we calculated the test of Xia *et al*.^68^ using DAMBE v. 5.3.108^69^, which showed little saturation even under the assumption of an asymmetrical tree. A partition homogeneity test was applied in PAUP* v. 4.0a150 to confirm the congruence of phylogenetic signals among all sequence data sets^70^. This test revealed the congruence of the phylogenetic signals among the sequence data sets, excluding those between 16S rRNA and 28S rDNA (Supplementary Table 9). However, we considered that the conflict between these data partitions does not affect the phylogeny because, following Whelan *et al*.^37^, it appears to be the result of copious homoplasy rather than independent histories of genes and the signal from their shared history is concordant in the combined analyses. This suggestion is confirmed by the congruence of the phylogenetic signals among the combined mtDNA data set (COI + 16S rRNA) and 28S rDNA (Supplementary Table 9).

To delimit species-level units, we used a molecular approach based on the concept of Molecular Operational Taxonomic Units (MOTUs thereafter)^71–74^, because current knowledge of the species-level taxonomy of the tropical Unionidae is very limited^39, 41, 42^. MOTUs were separated based on the Poisson Tree Processes (PTP) model to infer putative species boundaries on a phylogenetic input tree inferred from a maximum likelihood (ML) analysis^75^. The ML analysis was conducted based on an alignment of the COI haplotype sequences of Oriental and related Afrotropical taxa using RAxML v. 8.2.6 HPC Black Box^76^ at the San Diego Supercomputer Center through the CIPRES Science Gateway^77^. Three haplotypes of the Margaritiferidae species were used as an out-group (*Margaritifera laosensis*, *M. monodonta* and *M. dahurica*). Three in-group haplotypes (nos. 062 and 063 of *Parreysia* cf. *burmana* and no. 164 of *Lamellidens* aff. *corrianus* sp.1) were excluded from the final analysis, because they revealed doubtful placement in the COI phylogeny. This may reflect introgression events or incomplete lineage sorting, because the analysis of nuclear 28 S rDNA sequences supports the status of both taxa. We used a Bayesian implementation of the PTP model for species delimitation through an online bPTP server (http://species.h-its.org/ptp) with 500,000 Markov Chain Monte Carlo (MCMC) generations and 15% burn-in^75^. All out-group taxa were removed from the input tree using an appropriate option of the server. Additionally, we checked each MOTU using a 2% COI barcoding threshold, which was useful for the delimitation of the Unionidae lineages in Europe^78^. This approach has several limitations due to a taxon-specific variability in interspecific and intraspecific COI distances, especially within molluscan taxa^78–81^. However, it seems to be an appropriate method for preliminary delimitation of samples into putative (sub)species-level units together with PTP modelling, although we were unable to find a clear barcoding gap based on our COI data set (Supplementary Fig. 7). The PTP analysis based on the 192 unique COI haplotypes revealed that an estimated number of species in the in-group data set is between 67 and 92 with a mean value of 78.8 (acceptance rate: 0.2). The PTP solution with the highest support returned 76 putative species-level clusters. The majority of coalescent nodes corresponding to prospective species were well supported by the PTP Bayesian support values of ≥ 0.75 (Fig. 4), although three *Lamellidens* species revealed lower support values (0.58–0.73). In several cases, the modelling results were debatable. Two MOTUs with high support values ( > 0.90) were suggested within *Lamellidens exolescens*, but the maximal COI p-distance between these MOTUs was 0.45%. Additionally, three MOTUs were recorded within the *Parreysia* cf. *burmana* clade, but the maximal COI p-distance between them was 1.36%. By contrast, the *Parreysia* cf. *corrugata* clade, which divides into the two sub-clades with mean p-distance of 2.2%, was considered belonging to a single species. In this study, we treated both *Lamellidens exolescens* and *Parreysia* cf. *burmana* as species-level taxa. In general, our results support the solution that there are 72 prospective in-group species-level units from the Oriental and African regions.

The single-gene alignments were joined in combined multi-gene nucleotide sequence data set (COI + 16S rRNA + 28S rDNA) and collapsed into unique haplotypes (Supplementary Table 1) by using an online FASTA sequence toolbox (FaBox 1.41)^81^. Absent sites were treated as missing data. For phylogenetic analyses, we used the resulting combined data set with unique haplotypes, including those that were possibly identical but differed by the availability of gene partitions for certain specimens. Additionally, mtDNA and nDNA data sets and each gene partition were analyzed separately. In accordance with the results of the species delimitation analysis, only one sequence of each putative biological species remained within the in-group of the combined data set to avoid uncertainty in subsequent phylogenetic and biogeographic modelling (Lopes-Lima *et al*. 2017). The ML phylogenetic analysis was conducted using RAxML v. 8.2.6 HPC Black Box^76^ at the San Diego Supercomputer Center through the CIPRES Science Gateway^77^. We tested the following data sets: (i) five partitions (3 codons of COI + 16S rRNA + 28S rDNA), (ii) three partitions (COI + 16S rRNA + 28S rDNA), (iii) mtDNA with four partitions (3 codons of COI + 16S), (iv) mtDNA with two partitions (COI + 16S), (v) COI (3 codons), (vi) COI, and (vii) 28S. A unique GTR model was applied for each partition with corrections for gamma distribution. Nodal support values were estimated using an automatic, rapid bootstrapping algorithm according to the developer’s recommendation^82^, and the majority rule consensus tree was constructed from the independent searches. Bayesian inference (BI) analyses were performed in MrBayes v. 3.2.6^83^ at the San Diego Supercomputer Center through the CIPRES Science Gateway^77^. The partition combinations and each gene alignment tested were similar under the ML model. The best models of sequence evolution for each partition based on the corrected Akaike Information Criterion (AICc) of MEGA6^66^ are presented in Supplementary Table 10. Four runs, each with three heated (temperature = 0.1) and one cold Markov chain, were conducted for 50 million generations. Trees were sampled every 1000th generation. After completion of the MCMC analysis, the first 25% of trees were discarded as burn-in (pre-convergence part), and the majority rule consensus tree was calculated from the remaining trees. Convergence of the MCMC chains to a stationary distribution was checked visually based on the plotted posterior estimates using an MCMC trace analysis tool (Tracer v. 1.6)^84^. The effective sample size (ESS) value for each parameter sampled from the MCMC analysis was always recorded as > 5000. The combined set of trees showed a smooth frequency plot. To detect an incongruence of the optimal topology inferred from different sequence data sets, we conducted Repeatability Clade Analysis (RCA) of Bolotov *et al*.^36^ (Supplementary Table 2).

### Divergence time estimates

We estimated the acceptance of a global molecular clock to our multi-gene data set using the maximum likelihood test of MEGA6^66^, which revealed that the null hypothesis of equal evolutionary rate throughout the tree was rejected (*p* < 0.001). Thus, hypothetical divergence times were estimated in BEAST v. 1.8.3 based on multiple fossil calibration points using a lognormal relaxed clock algorithm with the Yule speciation process as the tree prior^85–87^. Calculations were performed at the San Diego Supercomputer Center through the CIPRES Science Gateway^77^. First, an uncalibrated ultrametric tree was obtained using BEAST. We specified similar settings to five partitions (3 codons of COI + 16S rRNA + 28S rDNA) as in the MrBayes analyses, but by using simplified evolutionary models. The HKY model was applied to each partition instead the GTR model, because the prior and posterior ESS values under the GTR model were recorded always < 100. This indicates that the GTR model is likely overly complex for our data. In accordance with the family-level phylogeny of Graf *et al*.^47^, we designated priors for out-group taxa using a “Monophyly” option of BEAUti v. 1.8.3^87^ as follows: (Hyriidae, (Iridinidae, Etheriidae, Mycetopodidae, (Unionidae, Margaritiferidae))). Two replicate searches were conducted, each with 100 million generations. The trees were sampled every 10,000th generation. The log files were checked visually with Tracer v. 1.6 for an assessment of the convergence of the MCMC chains and the effective sample size of parameters^84^. All ESS values were recorded as > 800; the posterior distributions were similar to the prior distributions. The resulting tree files from two independent analyses were compiled with LogCombiner v. 1.8.3 (Drummond *et al*.^87^). The first 10% of trees were discarded as an appropriate burn-in. The maximum clade credibility tree was obtained by using TreeAnnotator v. 1.8.3^87^.

From this uncalibrated ultrametric tree, we calculated an empirical scaling factor (ESF) for the evaluation of fossil-calibrated lineages using the node-to-tip length relative to the base of the tree and age of the oldest fossil in each lineage, consistent with the approach of Marshall^88^ (Supplementary Table 11). For time calibration of the molecular phylogeny, we used only fossil records that could be clearly assigned to be the most recent common ancestor (MRCA) of certain lineages with an ESF > 250 (Supplementary Table 11). In general, eight fossil calibrations were selected for timing of the phylogeny, including four new calibrations (Table 1). Additionally, we tested possible crown calibrations corresponding to the MRCA of Coelaturini, *Parreysia* and *Lamellidens* (Supplementary Table 12), but they were excluded from the analyses with respect to very low ESF values (<200), which could reveal incomplete fossil records of these lineages^88^. On this issue, the fossil calibrations that were used in the analyses are partly based on the out-group taxa, i.e., Hyriidae and Margaritiferidae, because fossil records of the Unionidae that could be directly assigned to certain recent lineages are relatively scarce^53^.

**Table 1:** List of fossil calibrations that were used in BEAST analyses.

Calibration no.	MRCA	Description	Reference
Calibration 1	*Lamprotula*	Hard minimum age: 34 Ma, †*Lamprotula hungi* Schneider, Böhme & Prieto, 2013. Diagnosis and phylogenetic placement: Medium large, sub-circular *Lamprotula* with relatively thin shell; ornamentation of irregular wrinkles on umbo; posterior area and posterior part of flank optionally ornamented with outward-bent flabellae and densely spaced, drop-shaped knobs; lateral teeth strongly arcuate, keel-like, without serration^53^. Stratigraphic horizon and locality: Na Duong Formation, Palaeogene, Cao Bang brickyard, Cao Bang Province, northern Vietnam^53^. Absolute age estimate: Eocene/Oligocene boundary, 34 Ma, based on stratigraphy; 95% soft upper bound 68 Ma (twice the age of the fossil). Prior setting: exponential distribution, mean (lambda) = 9.3, MRCA: *Lamprotula caveata* – *L. leaii*.	Present study: New stem calibration
Calibration 2	*Cristaria*	Hard minimum age: 34 Ma, †*Cristaria mothanica* Schneider, Böhme & Prieto, 2013. Diagnosis and phylogenetic placement: Oblique-oval *Cristaria* with markedly anterior positioned, slightly prosogyrous umbones, posterior half markedly higher than anterior part, and low posterior wing^53^. Stratigraphic horizon and locality: Na Duong and Rinh Chua formations, Palaeogene, Na Duong coalmine, Lang Son Province, northern Vietnam^53^. Absolute age estimate: Eocene/Oligocene boundary, 34 Ma, based on stratigraphy; 95% soft upper bound 68 Ma (twice the age of the fossil). Prior setting: exponential distribution, mean (lambda) = 9.3, MRCA: *Cristaria plicata* – *Cristaria* sp.	Present study: New stem calibration
Calibration 3	*Cuneopsis*	Hard minimum age: 34 Ma, †*Cuneopsis quangi* Schneider, Böhme & Prieto, 2013. Diagnosis and phylogenetic placement: Elongate, sub-rectangular, markedly inflated *Cuneopsis* with strongly prosogyrous umbones and slightly truncated posterior end^53^. Stratigraphic horizon and locality: Na Duong Formation, Palaeogene, Cao Bang brickyard, Cao Bang Province, northern Vietnam^53^. Absolute age estimate: Eocene/Oligocene boundary, 34 Ma, based on stratigraphy; 95% soft upper bound 68 Ma (twice the age of the fossil). Prior setting: exponential distribution, mean (lambda) = 9.3, MRCA: *Cuneopsis pisciculus* – *C. rufescens*.	Present study: New stem calibration
Calibration 4	*Margaritifera falcata* – *M. laevis*	Absolute age estimate: 46 Ma; 95% soft upper bound 92 Ma (twice the age of the fossil). Prior setting: exponential distribution, mean (lambda) = 12.5, MRCA: *Margaritifera falcata* – *M. laevis*.	Ref. 36: Crown calibration
Calibration 5	*Margaritifera margaritifera* – *M. dahurica*	Absolute age estimate: 34 Ma; 95% soft upper bound 68 Ma (twice the age of the fossil). Prior setting: exponential distribution, mean (lambda) = 9.3, MRCA: *Margaritifera margaritifera* – *M. dahurica*.	Ref. 36: Crown calibration
Calibration 6	Margaritiferidae	Hard minimum age: 129.4 Ma, †*Margaritifera idubedae* (Palacios & Sánchez, 1885). Diagnosis and phylogenetic placement: The assignation of this species to the genus is based on the shell shape, notable mantle attachment scars on the inner side of the valve and the arborescent rugosity of the muscle adductor scars^89^; it may however be a stem lineage of extant margaritiferids^36^. Stratigraphic horizon and locality: Hauterivian-Barremian Urbión Group, Valdehierro and Valdemadera sites, La Rioja Province, Cameros Basin, Spain^89^. Absolute age estimate: Hauterivian/Barremian boundary, 129.4 Ma, based on stratigraphy; 95% soft upper bound 259 Ma (twice the age of the fossil). Prior setting: exponential distribution, mean (lambda) = 35.1, MRCA: *M. auricularia* – *M. monodonta*.	Present study: New stem calibration
Calibration 7	Unionidae	Absolute age estimate: 155 Ma; 95% soft upper bound 310 Ma (twice the age of the fossil). Prior setting: exponential distribution, mean (lambda) = 42, MRCA: *Anodonta anatina* – *Parreysia olivacea*.	Ref. 47: Stem calibration
Calibration 8	‘The core Velesunioninae’ (*Velesunio* + *Alathyria* + *Lortiella* + *Microdontia*)	Absolute age estimate: 99.6 Ma; 95% soft upper bound 199 Ma (twice the age of the fossil). Prior setting: exponential distribution, mean (lambda) = 27, MRCA: *Alathyria pertexta* – *Velesunio ambiguus*.	Ref. 47: Stem calibration

Second, a calibrated phylogenetic tree was obtained based on these eight calibrations using the BEAST, LogCombiner and TreeAnnotator packages consistent with the approach and prior settings outlined above for an uncalibrated searching. Four replicate BEAST searches were conducted, each with 100 million generations. Trees were sampled every 10,000th generation. The log files were checked visually with Tracer v. 1.6 for an assessment of the convergence of the MCMC chains and the effective sample size of parameters^84^. The ESS values of each parameter were > 400 in each separate run and > 2000 in the combined data set. The posterior distributions were similar to the prior distributions. The resulting tree files from four independent analyses were compiled with LogCombiner v. 1.8.3^87^. The first 10% of trees from each run were discarded as an appropriate burn-in. The maximum clade credibility tree was obtained using TreeAnnotator v. 1.8.3^87^.

### Ancestral area reconstruction and diversification rate analyses

We tested ancestral area patterns using three different approaches, *i.e*., Statistical Dispersal-Vicariance Analysis (S-DIVA), Dispersal-Extinction Cladogenesis (Lagrange configurator, DEC), and Statistical Dispersal-Extinction Cladogenesis (S-DEC) implemented in RASP v. 3.2^90^. For the ancestral area reconstruction, we used the set of 36,004 fossil-calibrated binary trees that were combined from four runs of BEAST v. 1.8.3 (see above). As a condensed tree, we used the user-specified, fossil-calibrated consensus tree, which was obtained based on this set of trees using TreeAnnotator v. 1.8.3 (see above). From both of the tree data sets, out-group sequences were removed using the appropriate option of RASP v. 3.2. We coded seven possible distribution areas of the in-group taxa as follows: (a) Paleo-Mekong basin: the Mekong and Chao Phraya catchments, and rivers of the Malay Peninsula; (b) Western Indo-China: the Irrawaddy, Sittaung, Salween, Tavoy, and Kaladan catchments; (c) rivers of the Indian subcontinent; (d) tropical Africa: the Congo, Zambezi, and Nile drainage basins; (e) East Asia (from the Red River catchment to Far Eastern Russia); (f) Europe; and (g) North America (Supplementary Table 1). The approximate area of the paleo-Mekong catchment was estimated based on the results of comprehensive paleogeographic and phylogeographic reconstructions revealing ancient connections between the Mekong, Siam, Malacca Straits, North Sunda, and East Sunda paleo-river drainage basins^22, 28, 30, 31^. The S-DIVA models were calculated with the following parameters: max areas = 2; allow reconstruction with max reconstructions = 100; max reconstructions for final tree = 1000; and allowing extinctions. The DEC and S-DEC analyses were run with default settings and max areas = 2. In addition to the evaluations obtained from each analysis separately, we used generalized results of all three modeling approaches, which were combined using an algorithm implemented in RASP v. 3.2.

The diversification rates were assessed based on the combined Bayesian phylogeny across several endemic Indo-Chinese clades of naiads: (i) Rectidentinae; (ii) Rectidentini; (iii) Contradentini; and (iv) a Pseudodontinae clade from the paleo-Mekong basin. The set of 36,004 fossil-calibrated chronograms that were combined from four runs of BEAST v. 1.8.3 (see above) was used to construct semilogarithmic LTT plots in R-package ‘ape’ v. 4.0^19, 92^ with the supplement of ‘paleotree’ v. 2.7^93^. We did not use simulation for missing taxa^94^, because a total number of extant species in each group were uncertain. For example, Zieritz *et al*.^42^ list 9 valid species in Rectidentini, but our data set includes 13 species-level units. With respect to the same source, Pseudodontini from the Mekong and Chao Phraya catchments includes ~12 species (both *Pseudodon* and *Pilsbryoconcha*), which roughly correspond to our sample with 14 species. There are 12 nominal species of the Contradentini in Indo-China, including *Trapezoideus exolescens*, which is currently transferred to another genus, *Lamellidens* ^39^. Our sample with 13 species also corresponds to the remaining 11 nominal taxa. We therefore assumed that our samples of the Indo-Chinese clades of interest are nearly complete, which allows us to perform the diversification rate analyses. However, the true species richness of other clades of the Unionidae, as well as of the entire family, remains uncertain, because there is a plethora of nominal taxa, the status of which needs to be confirmed by using a molecular approach in the future^27, 41, 42^.

Two tests of a constant diversification rate for the endemic Indo-Chinese clades outlined above were calculated using ‘ape’ v. 4.0 based on the maximum clade credibility tree inferred from BEAST^91, 92^. First, was the analysis of diversification with three survival models, i.e., a constant diversification model, a variable diversification rate through time (Weibull model), and diversification changes at a specified time point^95^. As mean diversification rates, we used the delta parameter from the constant rate model of Paradis^95^. Additionally, beta values of the Weibull model were tested: *β* > 1 suggests declining, whereas *β* < 1 indicates an increasing rate of diversification. Second, the gamma statistic of Pybus and Harvey^94^ was applied. The null hypothesis of constant is rejected at the 5% level if a gamma statistic less than −1.645, which suggests a significantly decreasing rate of diversification through time^94^.

## Electronic supplementary material

Supplementary Information
