# Supplementary material for: Ancient River Inference Explains Exceptional Oriental Freshwater Mussel Radiations
Source: Sci Rep. 2017 May 18;7:2135. doi: 10.1038/s41598-017-02312-z (PMC5437074; doi:10.1038/s41598-017-02312-z)
Supplement: Supplementary file 1 — Supplementary Information [file 41598_2017_2312_MOESM1_ESM.pdf]

SUPPLEMENTARY INFORMATION

Ancient River Inference Explains Exceptional Oriental Freshwater Mussel Radiations

Ivan N. Bolotov*, Alexander V. Kondakov, Ilya V. Vikhrev, Olga V. Aksenova, Yulia V. Bespalaya, Mikhail Yu. Gofarov, Yulia S. Kolosova, Ekaterina S. Konopleva, Vitaly M. Spitsyn, Kitti Tanmuangpak and Sakboworn Tumpeesuwan

*Corresponding author: inepras@yandex.ru

Contents

Supplementary Table 1. List of sequences used in this study, including the species, the location and NCBI's GenBank accession numbers.

Supplementary Table 2. Results of Repeatability Clade Analysis (RCA) of main clades corresponding to the preferred topology

Supplementary Table 3. Diversification rate statistics for each endemic Indo-Chinese clade of the Unionidae

Supplementary Table 4. List of new sampling localities in Indo-China

Supplementary Table 5. List of sequenced specimens for each prospective biological species of the Unionidae from the Oriental Region over corresponding freshwater basins

Supplementary Table 6. Similarity matrix (Jaccard Similarity Index, %) between freshwater basins of the Oriental Region based on the presence/absence of each prospective biological species of the Unionidae

Supplementary Table 7. Primer sequences for PCR amplification and sequencing

Supplementary Table 8. Alignment length prior to and after treatment for length variability in GBlocks v. 0.91b

Supplementary Table 9. Probability (p-value) of phylogenetic conflict among sequence data sets from a partition-homogeneity test implemented in PAUP* v. 4.0a150

Supplementary Table 10. Models of sequence evolution for each partition based on corrected Akaike Information Criterion (AICc) of MEGA6 that were applied within a Bayesian inference framework

Supplementary Table 11. Evaluation of calibration lineages based on the empirical scaling factor (ESF) of Marshall

Supplementary Table 12. List of fossil calibrations that were not used in the BEAST analyses because of low ESF values (<100), which could reveal incomplete fossil records of the lineage

Supplementary Figure 1. Majority rule consensus phylogenetic tree of the Unionidae recovered from BI analysis of the complete data set of mitochondrial and nuclear sequences (five partitions: three codons of COI + 16S rRNA + 28S rDNA)

Supplementary Figure 2. Majority rule consensus phylogenetic tree of the Unionidae recovered from BI analysis of the mtDNA data set (four partitions: three codons of COI + 16S rRNA)

Supplementary Figure 3. Majority rule consensus phylogenetic tree of the Unionidae recovered from BI analysis of the nuclear 28S rDNA data set

Supplementary Figure 4. Fossil-calibrated ultrametric chronogram calculated under a lognormal relaxed clock model and a Yule process speciation implemented in BEAST 1.8.3 and obtained for the complete data set of mitochondrial and nuclear sequences (five partitions: three codons of COI + 16S rRNA + 28S rDNA)

Supplementary Figure 5. Historical biogeography of the Unionidae inferred from three different statistical modeling approaches, including (**a**) the combined results of S-DIVA, DEC and S-DEC; (**b**) S-DIVA; (**c**) DEC; and (**d**) S-DEC

Supplementary Figure 6. Plot of log species richness vs. clade age for selected families of the Unionoida

Supplementary Figure 7. Barcoding gap analyses for the Unionidae from the Oriental Region and related African taxa based on available COI sequences (N = 287)

Supplementary Table 1. List of sequences used in this study, including the species, the location and NCBI's GenBank accession numbers. Range Codes are as follows: (PM) Paleo-Mekong basin: Mekong and Chao Phraya catchments, and rivers of the Malay Peninsula; (WI) Western Indo-China: Irrawaddy, Sittaung, Salween, Tavoy, and Kaladan catchments; (IN) rivers of the Indian subcontinent; (AF) tropical Africa: Congo, Zambezi, and Nile drainage basins; (EA) East Asia (from the Red River catchment to Far Eastern Russia); (EU) Europe; and (NA) North America.

Putative species (MOTU)	Locality	Range Code	Specimen Voucher	Haplotype Code	COI	16S rRNA	28S rDNA	Reference
**In-Group Taxa**								
**UNIONIDAE Rafinesque, 1820**								
**PARREYSIINAE Henderson 1935**								
Lamellidens exolescens (Gould, 1843)	Myanmar: Tavoy River	WI	biv_145_3	Hap129	KX230532	KX230548	KX230559	Present study
Lamellidens exolescens (Gould, 1843)	Myanmar: Tavoy River	WI	biv_145_21	Hap129	KX230536	KX230550	KX230561	Present study
Lamellidens exolescens (Gould, 1843)	Myanmar: Tavoy River	WI	biv_146_2	Hap129	KX230537	KX230551	KX230562	Present study
Lamellidens exolescens (Gould, 1843)	Myanmar: Tavoy River	WI	biv_146_14	Hap129	KX230540	KX230552	KX230563	Present study
Lamellidens exolescens (Gould, 1843)	Myanmar: Tavoy River	WI	biv_145_12	Hap130	KX230535	KX230549	KX230560	Present study
Lamellidens exolescens (Gould, 1843)	Myanmar: Tavoy River	WI	biv_146_23	Hap131	KX230542	KX230553	KX230564	Present study
Lamellidens aff. *corrianus* (Lea, 1834) sp.1	Myanmar: Irrawaddy River	WI	UA: 20729.1/ UMMZ: 304642	Hap164	JN243903**	KP795042	JN243881	Ref. 37, 40
Lamellidens indawgyiensis Prashad, 1930	Myanmar: Irrawaddy River basin, Lake Indawgyi	WI	biv_109_1	Hap070	KX230544	KX230555	KX230566	Present study
Lamellidens indawgyiensis Prashad, 1930	Myanmar: Irrawaddy River basin, Lake Indawgyi	WI	biv_109_2	Hap071	KX230545	KX230556	KX230567	Present study
Lamellidens indawgyiensis Prashad, 1930	Myanmar: Irrawaddy River basin, Lake Indawgyi	WI	biv_109_3	Hap072	KX230546	KX230557	KX230568	Present study
Lamellidens indawgyiensis Prashad, 1930	Myanmar: Irrawaddy River basin, Chindwin River: Paukin Lake	WI	UA:20727.1/UMMZ:MC:304346	Hap167	JN243902	KF011263	JN243880	Ref. 37, 40
Lamellidens aff. *corrianus* (Lea, 1834) sp.2	India: Karli River basin, unnamed stream	IN	RNBI25	Hap165	JQ861226	n/a	n/a	GenBank
Lamellidens cf. *generosus* (Gould, 1847)	Myanmar: Salween River basin, Lake Inle	WI	biv_113_8	Hap042	KX865825	KX865596	KX865699	Present study
Lamellidens cf. *generosus* (Gould, 1847)	Myanmar: Salween River basin, Lake Inle	WI	biv_113_12	Hap043	KX865826	KX865597	KX865700	Present study
Lamellidens cf. *generosus* (Gould, 1847)	Myanmar: Salween River basin, Lake Inle	WI	biv_113_14	Hap044	KX865827	KX865598	KX865701	Present study
Lamellidens cf. *generosus* (Gould, 1847)	Myanmar: Salween River basin, Lake Inle, a channel in Nuangshve	WI	biv_112_7	Hap106	KX865828	KX865599	KX865702	Present study

Putative species (MOTU)	Locality	Range Code	Specimen Voucher	Haplotype Code	COI	16S rRNA	28S rDNA	Reference
Lamellidens cf. *generosus* (Gould, 1847)	Myanmar: Salween River basin, Lake Inle, a channel in Nuangshve	WI	biv_112_12	Hap106	KX865829	KX865600	KX865703	Present study
Lamellidens cf. *generosus* (Gould, 1847)	Myanmar: Salween River basin, Lake Inle, a channel in Nuangshve	WI	biv_112_15	Hap106	KX865830	KX865601	KX865704	Present study
Lamellidens cf. *generosus* (Gould, 1847)	Myanmar: Salween River basin, Nam Pilu River	WI	biv_141_5	Hap123	KX865831	KX865602	KX865705	Present study
Lamellidens cf. *generosus* (Gould, 1847)	Myanmar: Salween River basin, Nam Pilu River	WI	biv_141_9	Hap124	KX865832	KX865603	KX865706	Present study
Lamellidens cf. *generosus* (Gould, 1847)	Myanmar: Salween River basin, Nam Pilu River	WI	biv_141_18	Hap125	KX865833	KX865604	KX865707	Present study
Lamellidens cf. *generosus* (Gould, 1847)	Myanmar: Salween River basin, Snake Stream	WI	biv_142_1	Hap125	KX865834	KX865605	KX865708	Present study
Lamellidens aff. *marginalis* (Lamarck, 1819) sp.1	India	IN	SBM12	Hap169	KF690121	n/a	n/a	GenBank
Lamellidens aff. *marginalis* (Lamarck, 1819) sp.5	India	IN	SBM2	Hap166	KF690119	n/a	n/a	GenBank
Lamellidens aff. *marginalis* (Lamarck, 1819) sp.2	India: Karli River basin, Bangsaal River	IN	RNBI19	Hap170	JQ861227	n/a	n/a	GenBank
Lamellidens aff. *marginalis* (Lamarck, 1819) sp.3	Myanmar: Kaladan River basin, unnamed puddle	WI	biv_153	Hap136	KX230547	KX230558	KX230569	Present study
Lamellidens aff. *marginalis* (Lamarck, 1819) sp.4	India	IN	SBM8	Hap168	KF690117	n/a	n/a	GenBank
Parreysia cf. *corrugata* (Müller, 1774)	India: Karli River basin, Pitdhaval River	IN	RNBI17	Hap142	JQ861240	n/a	n/a	GenBank
Parreysia cf. *corrugata* (Müller, 1774)	India: Karli River basin, Pitdhaval River	IN	RNBI18	Hap143	JQ861239	n/a	n/a	GenBank
Parreysia cf. *corrugata* (Müller, 1774)	India: Krishna River basin, Ghataprabha River	IN	RNBI2	Hap140	JQ861237	n/a	n/a	GenBank
Parreysia cf. *corrugata* (Müller, 1774)	India: Karli River basin, Pitdhaval River	IN	RNBI14	Hap141	JQ861230	n/a	n/a	GenBank
Parreysia cf. *corrugata* (Müller, 1774)	India	IN	SBM3	Hap144	KF690110	n/a	n/a	GenBank
Parreysia cf. *corrugata* (Müller, 1774)	India	IN	SBM27	Hap145	KF690111	n/a	n/a	GenBank
Parreysia olivacea Prashad, 1930	Myanmar: Irrawaddy River	WI	UMMZ:304641	Hap139	KP795022	KP795044	KP795005	Ref. 40
Parreysia tavoyensis (Gould, 1843)	Myanmar: Tavoy River	WI	biv_149	Hap135	KX230543	KX230554	KX230565	Present study
Parreysia tavoyensis (Gould, 1843)	Myanmar: Irrawaddy River	WI	UA20726.1/ UMMZ:304640	Hap137	JN243901	KP795043	JN243877	Ref. 37, 40
Parreysia tavoyensis (Gould, 1843)	Myanmar: Irrawaddy River	WI	UA: 20722.1	Hap138	JN243900	n/a	JN243876	Ref. 37
Nitia teretiuscula (Philippi, 1847)	Egypt: Nile River	AF	ANSP:416305/ UA:20993.1	Hap173	JN243897	n/a	JN243875	Ref. 37
Nitia teretiuscula (Philippi, 1847)	Egypt: Nile River	AF	ANSP:416305	Hap174	KJ081160	n/a	JN243875	Ref. 32
Coelatura gabonensis (Küster, 1862)	Congo River	AF	ANSP:A21417/ UA:21018.1	Hap156	JN243895	n/a	JN243873	Ref. 37
Coelatura mweruensis (Smith, 1908)	Zambia: Congo River basin, Lake Mweru	AF	UMMZ:304377	Hap161	KJ081174	n/a	KJ081189	Ref. 32

Putative species (MOTU)	Locality	Range Code	Specimen Voucher	Haplotype Code	COI	16S rRNA	28S rDNA	Reference
Coelatura mweruensis (Smith, 1908)	Zambia: Congo River basin, Lake Bangweulu	AF	ANSP:416276	Hap157	KJ081167	n/a	KJ081186	Ref. 32
Coelatura mweruensis (Smith, 1908)	Zambia: Congo River basin, Lake Bangweulu	AF	ANSP:416276	Hap158	KJ081168	n/a	KJ081187	Ref. 32
Coelatura mweruensis (Smith, 1908)	Zambia: Congo River basin, Chembeshi River basin, Kalungu River	AF	ANSP:416247	Hap159	KJ081169	n/a	KJ081188	Ref. 32
Coelatura mweruensis (Smith, 1908)	Zambia: Congo River basin, Chembeshi River basin, Kalungu River	AF	ANSP:416247	Hap160	KJ081170	n/a	KJ081188	Ref. 32
Coelatura mweruensis (Smith, 1908)	Zambia: Congo River basin, Lake Mweru	AF	UMMZ:304377	Hap162	KJ081175	n/a	KJ081190	Ref. 32
Coelatura mweruensis (Smith, 1908)	Zambia: Congo River basin, Lake Mweru	AF	UMMZ:304377	Hap163	KJ081176	n/a	KJ081191	Ref. 32
Coelatura mweruensis (Smith, 1908)	Zambia: Congo River basin, Lake Mweru	AF	UMMZ:304379	Hap171	KJ081180	n/a	KJ081193	Ref. 32
Coelatura mweruensis (Smith, 1908)	Zambia: Congo River basin, Lake Mweru	AF	ANSP:416363/ UA:20834.1	Hap172	JN243896	n/a	JN243874	Ref. 37
Coelatura kunenensis (Mousson, 1887)	Zambia: Zambezi River	AF	ANSP:419411	Hap154	KJ081164	n/a	KJ081184	Ref. 32
Coelatura kunenensis (Mousson, 1887)	Zambia: Zambezi River	AF	ANSP:419411	Hap155	KJ081165	n/a	KJ081185	Ref. 32
Coelatura aff. *aegyptiaca* (Cailliaud, 1827) sp.1	Egypt: Nile River	AF	ANSP:416304	Hap152	JN243894	KP795045	JN243872	Ref. 37, 40
Coelatura aff. *aegyptiaca* (Cailliaud, 1827) sp.2	Egypt: Nile River	AF	ANSP:416304	Hap153	JN243892	n/a	JN243870	Ref. 37
Radiatula aff. *caerulea* (Lea, 1831)	Myanmar: Irrawaddy River basin, Lake Indawgyi	WI	biv_108 _1	Hap098	KX865835	KX865606	KX865709	Present study
Radiatula aff. *caerulea* (Lea, 1831)	Myanmar: Irrawaddy River basin, Lake Indawgyi	WI	biv_108 _2	Hap099	KX865836	KX865607	n/a	Present study
Radiatula aff. *caerulea* (Lea, 1831)	Myanmar: Irrawaddy River basin, Lake Indawgyi	WI	biv_108 _3	Hap100	KX865837	KX865608	n/a	Present study
Radiatula aff. *khadakvaslaensis* (Ray, 1966) sp.1	India	IN	SBM7	Hap148	KF690124	n/a	n/a	GenBank
Radiatula aff. *khadakvaslaensis* (Ray, 1966) sp.2	India	IN	SBM15	Hap149	KF690107	n/a	n/a	GenBank
Radiatula aff. *khadakvaslaensis* (Ray, 1966) sp.3	India: Karli River basin, Bangsaal River	IN	RNBI7	Hap147	JQ861244	n/a	n/a	GenBank
Radiatula cf. *bonneaudii* (Eydoux, 1838)	Myanmar: Irrawaddy River	WI	UA:20714.2/U MMZ: 304645	Hap146	JN243898	KP795047	JN243878	Ref. 37, 40
Parreysia cf. *burmana* (Blanford, 1869)	Myanmar: Irrawaddy River basin, Lake Indawgyi	WI	biv_107_1	Hap062	KX865838**	KX865609	KX865710	Present study
Parreysia cf. *burmana* (Blanford, 1869)	Myanmar: Irrawaddy River basin, Lake Indawgyi	WI	biv_107_2	Hap063	KX865839**	KX865610	KX865711	Present study
Parreysia cf. *burmana* (Blanford, 1869)	Myanmar: Irrawaddy River basin, Lake Indawgyi	WI	biv_107_3	Hap064	KX865840	KX865611	KX865712	Present study
Parreysia cf. *burmana* (Blanford, 1869)	Myanmar: Irrawaddy River basin, Lake Indawgyi	WI	biv_106_1	Hap101	KX865841	KX865612	KX865713	Present study
Parreysia cf. *burmana* (Blanford, 1869)	Myanmar: Irrawaddy River basin, Lake Indawgyi	WI	biv_106_3	Hap101	KX865842	KX865614	KX865714	Present study

Putative species (MOTU)	Locality	Range Code	Specimen Voucher	Haplotype Code	COI	16S rRNA	28S rDNA	Reference
Parreysia cf. *burmana* (Blanford, 1869)	Myanmar: Irrawaddy River basin, Lake Indawgyi	WI	biv_106_2	Hap102	KX865843	KX865613	KX865715	Present study
Radiatula aff. *humilis* (Lea, 1856) sp.1	Thailand: Mekong River basin, Chi River	PM	biv_126_1	Hap058	KX865844	KX865615	KX865716	Present study
Radiatula aff. *humilis* (Lea, 1856) sp.1	Thailand: Mekong River basin, Chi River	PM	biv_126_2	Hap059	KX865845	KX865616	KX865717	Present study
Radiatula aff. *humilis* (Lea, 1856) sp.1	Thailand: Mekong River basin, Chi River	PM	biv_126_3	Hap060	KX865846	KX865617	KX865718	Present study
Radiatula aff. *humilis* (Lea, 1856) sp.1	Thailand: Mekong River basin, Chi River	PM	biv_129 _1	Hap092	KX865847	KX865618	KX865719	Present study
Radiatula aff. *humilis* (Lea, 1856) sp.1	Thailand: Mekong River basin, Chi River	PM	biv_129 _3	Hap092	KX865848	KX865620	KX865720	Present study
Radiatula aff. *humilis* (Lea, 1856) sp.1	Thailand: Mekong River basin, Chi River	PM	biv_129 _2	Hap093	KX865849	KX865619	KX865721	Present study
Radiatula aff. *humilis* (Lea, 1856) sp.2	Thailand: Mekong River basin, Chi River	PM	biv_124_1	Hap079	KX865850	KX865621	KX865722	Present study
Radiatula aff. *humilis* (Lea, 1856) sp.2	Thailand: Mekong River basin, Chi River	PM	biv_124_2	Hap080	KX865851	KX865622	KX865723	Present study
Scabies aff. *crispata* (Gould, 1843)	Cambodia: Mekong River basin	PM	UMMZ:304646	Hap150	KP795023	KP795048	KP795006	Ref. 40
Scabies crispata (Gould, 1843)	Myanmar: Tavoy River	WI	biv_147_3	Hap132	KX865852	KX865623	KX865724	Present study
Scabies crispata (Gould, 1843)	Myanmar: Tavoy River	WI	biv_147_10	Hap132	KX865853	KX865624	KX865725	Present study
Scabies crispata (Gould, 1843)	Myanmar: Tavoy River	WI	biv_147_18	Hap132	KX865854	KX865625	KX865726	Present study
Scabies crispata (Gould, 1843)	Myanmar: Tavoy River	WI	biv_148_4	Hap132	KX865855	KX865626	KX865727	Present study
Scabies crispata (Gould, 1843)	Myanmar: Tavoy River	WI	biv_148_7	Hap133	KX865856	KX865627	KX865728	Present study
Scabies crispata (Gould, 1843)	Myanmar: Tavoy River	WI	biv_148_15	Hap134	KX865857	KX865628	KX865729	Present study
Oxynaia cf. *pugio* (Benson, 1862)	Myanmar: Irrawaddy River basin, Chindwin River: Paukin Lake	WI	UA:20739.1/UMMZ: 304644	Hap151	JN243899	KP795046	JN243879	Ref. 37, 40
**PSEUDODONTINAE Frierson, 1927**								
Pseudodon cf. *avae* (Theobald, 1873)	Myanmar: Irrawaddy River basin, a tributary of Lake Indawgyi	WI	biv_110_5	Hap089	KX865858	KX865629	KX865730	Present study
Pseudodon cf. *avae* (Theobald, 1873)	Myanmar: Irrawaddy River basin, a tributary of Lake Indawgyi	WI	biv_110_10	Hap089	KX865859	KX865630	KX865731	Present study
Pseudodon cf. *avae* (Theobald, 1873)	Myanmar: Irrawaddy River basin, a tributary of Lake Indawgyi	WI	biv_110_11	Hap091	KX865860	KX865631	KX865732	Present study
Pseudodon aff. *inoscularis* (Gould, 1844) sp.1	SE Asia	PM	H418.f	Hap181	DQ206793	n/a	n/a	GenBank
Pseudodon cf. *inoscularis* (Gould, 1844)	Cambodia: Mekong River basin	PM	UMMZ:304349/UMMZ:MC:304349	Hap178	KP795027	KF011261	KP795010	Ref. 40
Pseudodon aff. vondembuschianus (Lea, 1840) sp.1	Thailand: Mekong River basin, Phong River	PM	biv122	Hap109	KX865861	KX865632	KX865733	Present study
Pseudodon aff. vondembuschianus (Lea, 1840) sp.2	Laos: Mekong River basin	PM	UMMZ:304650	Hap180	KP795029	KP795052	AF400694	Ref. 40, 96

Putative species (MOTU)	Locality	Range Code	Specimen Voucher	Haplotype Code	COI	16S rRNA	28S rDNA	Reference
Pseudodon cambodjensis (Petit de la Saussaye, 1865)	Cambodia: Mekong River basin, Tonle Sap River: Pursat River	PM	UMMZ:304350/ UMMZ:MC:304 350	Hap179	KP795028	KF011262	KP795011	Ref. 40
Pseudodon cambodjensis (Petit de la Saussaye, 1865)	Thailand	PM	n/a	Hap200	KX822660	n/a	KX822616	Ref. 27
Pseudodon cambodjensis (Petit de la Saussaye, 1865)	Malaysia	PM	X154	Hap210	KX051299	n/a	n/a	Ref. 41
Pseudodon cambodjensis (Petit de la Saussaye, 1865)	Malaysia	PM	BIV1658	Hap211	KX051298	n/a	n/a	Ref. 41
Pseudodon cambodjensis (Petit de la Saussaye, 1865)	Malaysia	PM	X198	Hap212	KX051297	n/a	n/a	Ref. 41
Pseudodon cf. ellipticus Conrad, 1865	Thailand: Mekong River basin, Phong River	PM	biv_120_4	Hap048	KX865862	KX865633	KX865734	Present study
Pseudodon cf. ellipticus Conrad, 1865	Thailand: Mekong River basin, Phong River	PM	biv_120_12	Hap049	KX865863	KX865634	KX865735	Present study
Pseudodon cf. ellipticus Conrad, 1865	Thailand: Mekong River basin, Phong River	PM	biv_120_15	Hap049	KX865864	KX865635	KX865736	Present study
Pseudodon cf. ellipticus Conrad, 1865	Thailand: Mekong River basin, Phong River	PM	biv_120_3	Hap052	KX865865	KX865636	KX865737	Present study
Pseudodon cf. ellipticus Conrad, 1865	Thailand: Mekong River basin, Phong River	PM	biv_120_8	Hap053	KX865866	KX865637	KX865738	Present study
Pseudodon cf. ellipticus Conrad, 1865	Thailand: Mekong River basin, Phong River	PM	biv_120_14	Hap054	KX865867	KX865638	KX865739	Present study
Pseudodon cf. ellipticus Conrad, 1865	Thailand: Mekong River basin, Phong River	PM	biv120_7	Hap065	KX865868	KX865639	KX865740	Present study
Pseudodon cf. ellipticus Conrad, 1865	Thailand: Mekong River basin, Phong River	PM	biv120_11	Hap066	KX865869	KX865640	KX865741	Present study
Pseudodon cf. ellipticus Conrad, 1865	Thailand: Mekong River basin, Phong River	PM	biv120_13	Hap067	KX865870	KX865641	KX865742	Present study
Pseudodon cf. ellipticus Conrad, 1865	Thailand: Mekong River basin, Phong River	PM	biv205_4	Hap191	KY561622	KY561640	KY561654	Present study
Pilsbryoconcha aff. compressa (Martens, 1860) sp.1	Thailand: Mekong River basin, Chi River	PM	biv_125_1	Hap086	KX865871	KX865642	KX865743	Present study
Pilsbryoconcha cf. compressa (Martens, 1860)	Thailand: Mekong River basin, artificial pond near the Ban Nong-Bua village	PM	biv_116_1	Hap055	KX865872	KX865643	KX865744	Present study
Pilsbryoconcha cf. compressa (Martens, 1860)	Thailand: Mekong River basin, artificial pond near the Ban Nong-Bua village	PM	biv_116_2	Hap056	KX865873	KX865644	KX865745	Present study
Pilsbryoconcha cf. compressa (Martens, 1860)	Thailand: Mekong River basin, artificial pond near the Ban Nong-Bua village	PM	biv_116_3	Hap057	KX865874	KX865645	n/a	Present study
Pilsbryoconcha cf. compressa (Martens, 1860)	Thailand: Mekong River basin, Huai Nam Khu Reservoir	PM	biv_118	Hap107	KX865875	KX865646	KX865746	Present study
Pilsbryoconcha cf. compressa (Martens, 1860)	Malaysia	PM	BIV1607	hap055	KX051285	n/a	n/a	Ref. 41
Pilsbryoconcha cf. compressa (Martens, 1860)	Thailand	PM	n/a	Hap198	KX822656	n/a	KX822613	Ref. 27
Pilsbryoconcha cf. compressa (Martens, 1860)	Malaysia	PM	X257	Hap218	KX051283	n/a	n/a	Ref. 41
Pilsbryoconcha cf. compressa	Malaysia	PM	X278	Hap219	KX051284	n/a	n/a	Ref. 41

Putative species (MOTU)	Locality	Range Code	Specimen Voucher	Haplotype Code	COI	16S rRNA	28S rDNA	Reference
(Martens, 1860)								
Pilsbryoconcha aff. exilis (Lea, 1838) sp.1	Cambodia: Mekong River basin	PM	UMMZ:304647	Hap175	KP795024	KP795049	KP795007	Ref. 40
Pilsbryoconcha cf. exilis (Lea, 1838)	Malaysia	PM	X213	Hap206	KX051289	n/a	n/a	Ref. 41
Pilsbryoconcha cf. exilis (Lea, 1838)	Malaysia	PM	X85	Hap206	KX051290	n/a	n/a	Ref. 41
Pilsbryoconcha cf. exilis (Lea, 1838)	Malaysia	PM	X200	Hap206	KX051286	n/a	n/a	Ref. 41
Pilsbryoconcha cf. exilis (Lea, 1838)	Malaysia	PM	X131	Hap206	KX051287	n/a	n/a	Ref. 41
Pilsbryoconcha cf. exilis (Lea, 1838)	Malaysia	PM	X129	Hap206	KX051288	n/a	n/a	Ref. 41
Pilsbryoconcha aff. exilis (Lea, 1838) sp.2	Malaysia	PM	X219	Hap220	KX051291	n/a	n/a	Ref. 41
Pseudodon aff. mouhotii (Lea, 1863) sp.1	Laos: Mekong River basin, Nam Long River	PM	182_2	Hap110	KX865876	KX865647	KX865747	Present study
Pseudodon aff. mouhotii (Lea, 1863) sp.1	Laos: Mekong River basin, Nam Pe River	PM	183_4	Hap110	KX865877	KX865648	KX865748	Present study
Pseudodon aff. mouhotii (Lea, 1863) sp.1	Laos: Mekong River basin, Nam Long River	PM	182_19	Hap113	KX865878	KX865649	KX865749	Present study
Pseudodon aff. mouhotii (Lea, 1863) sp.1	Laos: Mekong River basin, a tributary of Nam Fa River near Vieng Phou Kha	PM	biv201_2	Hap187	KY561623	KY561641	KY561655	Present study
Pseudodon aff. mouhotii (Lea, 1863) sp.1	Laos: Mekong River basin, a tributary of Nam Fa River near Vieng Phou Kha	PM	biv201_3	Hap187	KY561624	KY561642	KY561656	Present study
Pseudodon aff. mouhotii (Lea, 1863) sp.1	Laos: Mekong River basin, a tributary of Nam Fa River near Vieng Phou Kha	PM	biv201_5	Hap187	KY561625	KY561643	KY561657	Present study
Pseudodon aff. mouhotii (Lea, 1863) sp.1	Laos: Mekong River basin, a tributary of Nam Fa River near Vieng Phou Kha	PM	biv202_1	Hap187	KY561626	KY561644	KY561658	Present study
Pseudodon aff. mouhotii (Lea, 1863) sp.2	Thailand: Mekong River basin, Loei River	PM	biv_119_1	Hap045	KX865879	KX865650	KX865750	Present study
Pseudodon aff. mouhotii (Lea, 1863) sp.2	Thailand: Mekong River basin, Loei River	PM	biv_119_2	Hap045	KX865880	KX865651	KX865751	Present study
Pseudodon aff. mouhotii (Lea, 1863) sp.2	Thailand: Mekong River basin, Loei River	PM	biv_119_3	Hap047	KX865881	KX865652	KX865752	Present study
Pseudodon aff. mouhotii (Lea, 1863) sp.2	Thailand: Mekong River basin, Loei River	PM	biv_119_4	Hap047	KX865882	KX865653	KX865753	Present study
Pseudodon aff. mouhotii (Lea, 1863) sp.3	Laos: Mekong River basin	PM	UMMZ:304649	Hap177	KP795026	KP795051	KP795009	Ref. 40
Pseudodon aff. mouhotii (Lea, 1863) sp.3	Laos: Mekong River basin	PM	NCSM84903	Hap202	KX822663	n/a	KX822619	Ref. 27
Pseudodon cumingii (Lea, 1850)	Laos: Mekong River basin	PM	UMMZ:304648	Hap176	KP795025	KP795050	KP795008	Ref. 40
Pseudodon cumingii (Lea, 1850)	Laos: Mekong River basin	PM	NCSM84884	Hap201	KX822662	n/a	KX822618	Ref. 27
Pseudodon sp.1	Malaysia	PM	X115	Hap214	KX051295	n/a	n/a	Ref. 41
Pseudodon sp.1	Malaysia	PM	X79	Hap216	KX051292	n/a	n/a	Ref. 41
Pseudodon sp.1	Malaysia	PM	X89	Hap217	KX051293	n/a	n/a	Ref. 41
Pseudodon vondembuschianus (Lea, 1840)	Malaysia	PM	BIV1693	Hap204	KX051311	n/a	n/a	Ref. 41

Putative species (MOTU)	Locality	Range Code	Specimen Voucher	Haplotype Code	COI	16S rRNA	28S rDNA	Reference
Pseudodon vondembuschianus (Lea, 1840)	Malaysia	PM	X38	Hap204	KX051308	n/a	n/a	Ref. 41
Pseudodon vondembuschianus (Lea, 1840)	Malaysia	PM	X39	Hap204	KX051307	n/a	n/a	Ref. 41
Pseudodon vondembuschianus (Lea, 1840)	Malaysia	PM	X001	Hap204	KX051305	n/a	n/a	Ref. 41
Pseudodon vondembuschianus (Lea, 1840)	Malaysia	PM	BIV1669	Hap204	KX051302	n/a	n/a	Ref. 41
Pseudodon vondembuschianus (Lea, 1840)	Malaysia	PM	BIV1692	Hap204	KX051300	n/a	n/a	Ref. 41
Pseudodon vondembuschianus (Lea, 1840)	Malaysia	PM	X157	Hap205	KX051306	n/a	n/a	Ref. 41
Pseudodon vondembuschianus (Lea, 1840)	Malaysia	PM	BIV1657	Hap205	KX051304	n/a	n/a	Ref. 41
Pseudodon vondembuschianus (Lea, 1840)	Malaysia	PM	BIV1670	Hap205	KX051301	n/a	n/a	Ref. 41
Pseudodon vondembuschianus (Lea, 1840)	Malaysia	PM	BIV1822	Hap207	KX051310	n/a	n/a	Ref. 41
Pseudodon vondembuschianus (Lea, 1840)	Malaysia	PM	X32	Hap208	KX051309	n/a	n/a	Ref. 41
Pseudodon vondembuschianus (Lea, 1840)	Malaysia	PM	BIV1806	Hap209	KX051303	n/a	n/a	Ref. 41
Pseudodon vondembuschianus (Lea, 1840)	Malaysia	PM	BIV1721	Hap213	KX051296	n/a	n/a	Ref. 41
**RECTIDENTINAE Modell, 1942**								
Trapezoideus sp.1	Laos: Mekong River basin, Nam Ou River	PM	UMMZ:304347/ UMMZ:MC:304 347	Hap186	KP795036	KF011265	KP795018	Ref. 40
Contradens sp.1	Cambodia: Mekong River basin	PM	UMMZ:304653	Hap185	KP795035	KP795055	KP795017	Ref. 40
Physunio cf. modelli Brandt, 1974	Thailand: Mekong River basin, Chi River	PM	biv_125_2	Hap087	KX865883	KX865654	KX865754	Present study
Physunio cf. modelli Brandt, 1974	Thailand: Mekong River basin, Chi River	PM	biv_125_3	Hap088	KX865884	KX865655	KX865755	Present study
Physunio cf. modelli Brandt, 1974	Thailand: Mekong River basin, Chi River	PM	biv_131_1	Hap087	KX865885	n/a	KX865756	Present study
Physunio cf. modelli Brandt, 1974	Thailand: Mekong River basin, Chi River	PM	biv_131_2	Hap087	KX865886	n/a	KX865757	Present study
Physunio cf. modelli Brandt, 1974	Thailand: Mekong River basin, Chi River	PM	biv_131_3	Hap087	KX865887	n/a	KX865758	Present study
Physunio cf. modelli Brandt, 1974	Thailand: Mekong River basin, Chi River	PM	biv_131	Hap108	KX865888	n/a	KX865759	Present study
Physunio cf. modelli Brandt, 1974	Thailand: Mekong River basin, Phong River	PM	biv205_1	Hap190	KY561627	n/a	KY561659	Present study
Physunio cf. modelli Brandt, 1974	Thailand: Mekong River basin, Phong River	PM	biv205_2	Hap087	KY561628	n/a	KY561660	Present study
Physunio cf. modelli Brandt, 1974	Thailand: Mekong River basin, Phong River	PM	biv205_3	Hap087	KY561629	n/a	KY561661	Present study
Physunio cf. modelli Brandt, 1974	Thailand	PM	n/a	Hap192	KX822655	n/a	n/a	Ref. 27

Putative species (MOTU)	Locality	Range Code	Specimen Voucher	Haplotype Code	COI	16S rRNA	28S rDNA	Reference
Contradens sp.2	Myanmar: Irrawaddy River basin, Nanuinhka Chaung River	WI	biv_111_2	Hap032	KX865889	KX865656	KX865760	Present study
Contradens sp.2	Myanmar: Irrawaddy River basin, Nanuinhka Chaung River	WI	biv_111_21	Hap032	KX865890	KX865657	KX865761	Present study
Contradens sp.2	Myanmar: Irrawaddy River basin, Nanuinhka Chaung River	WI	biv_111_43	Hap034	KX865891	KX865658	KX865762	Present study
Contradens sp.2	Myanmar: Irrawaddy River basin, Mali Hka River basin, Pan Khai stream	WI	biv_101_4	Hap036	KX865892	n/a	n/a	Present study
Contradens sp.2	Myanmar: Irrawaddy River basin, Mali Hka River basin, Nam Shu River	WI	biv_105_24	Hap036	KX865893	n/a	n/a	Present study
Contradens sp.2	Myanmar: Irrawaddy River basin, Mali Hka River basin, Pan Khai stream	WI	biv_101_5	Hap036	KX865894	KX865659	KX865763	Present study
Contradens sp.2	Myanmar: Irrawaddy River basin, Mali Hka River basin, Pan Khai stream	WI	biv_101_6	Hap036	KX865895	n/a	KX865764	Present study
Contradens sp.2	Myanmar: Irrawaddy River basin, Mali Hka River basin, Mansakun River	WI	biv_103_17	Hap036	KX865896	n/a	KX865765	Present study
Contradens sp.2	Myanmar: Irrawaddy River basin, Mali Hka River basin, Mansakun River	WI	biv_103_18	Hap036	KX865897	n/a	KX865766	Present study
Contradens sp.2	Myanmar: Irrawaddy River basin, Mali Hka River basin, Mansakun River	WI	biv_103_19	Hap036	KX865898	n/a	KX865767	Present study
Contradens sp.2	Myanmar: Irrawaddy River basin, Mali Hka River basin, Nam Balak River	WI	biv_102_7	Hap039	KX865899	KX865660	KX865768	Present study
Contradens sp.2	Myanmar: Irrawaddy River basin, Mali Hka River basin, Nam Balak River	WI	biv_102_11	Hap036	KX865900	KX865661	KX865769	Present study
Contradens sp.2	Myanmar: Irrawaddy River basin, Mali Hka River basin, Nam Balak River	WI	biv_102_14	Hap036	KX865901	KX865662	KX865770	Present study
Contradens sp.2	Myanmar: Irrawaddy River basin, Mali Hka River basin, Nam Shu River	WI	biv_105_31	Hap039	KX865902	n/a	KX865771	Present study
Contradens sp.2	Myanmar: Irrawaddy River basin, Mali Hka River basin, Nam Shu River	WI	biv_105_32	Hap039	KX865903	n/a	KX865772	Present study
Contradens sp.2	Myanmar: Irrawaddy River basin, Mali Hka River basin, unnamed stream	WI	biv_104_34	Hap039	KX865904	n/a	KX865773	Present study
Contradens sp.2	Myanmar: Irrawaddy River basin, Mali Hka River basin, unnamed stream	WI	biv_104_35	Hap039	KX865905	n/a	KX865774	Present study
Contradens sp.2	Myanmar: Irrawaddy River basin, Mali Hka River basin, Pan Khai stream	WI	biv_101_1	Hap035	n/a	n/a	KX865775	Present study
Contradens sp.2	Myanmar: Irrawaddy River basin, Mali Hka River basin, Nam Shu River	WI	biv_105_22	Hap035	n/a	n/a	KX865776	Present study
Contradens sp.3	Myanmar: Sittaung River basin, Tauk Ue Kupt River	WI	biv_144_14	Hap127	KX865906	KX865663	KX865777	Present study
Contradens sp.3	Myanmar: Sittaung River basin, Tauk Ue Kupt River	WI	biv_144_25	Hap127	KX865907	KX865664	KX865778	Present study
Contradens sp.3	Myanmar: Sittaung River basin, Tauk Ue Kupt River	WI	biv_144_19	Hap128	KX865908	KX865665	KX865779	Present study
Contradens sp.4	Myanmar: Sittaung River basin, Kyan Hone River	WI	biv_138_4	Hap115	KX865909	KX865666	KX865780	Present study
Contradens sp.4	Myanmar: Sittaung River basin, Kyan Hone River	WI	biv_138_7	Hap115	KX865910	KX865667	KX865781	Present study

Putative species (MOTU)	Locality	Range Code	Specimen Voucher	Haplotype Code	COI	16S rRNA	28S rDNA	Reference
Contradens sp.4	Myanmar: Sittaung River basin, Kyan Hone River	WI	biv_155_4	Hap115	KX865911	KX865668	KX865782	Present study
Contradens sp.4	Myanmar: Sittaung River basin, Kyan Hone River	WI	biv_155_25	Hap115	KX865912	KX865669	KX865783	Present study
Contradens sp.4	Myanmar: Sittaung River basin, Kyan Hone River	WI	biv_138_12	Hap116	KX865913	KX865670	KX865784	Present study
Contradens sp.4	Myanmar: Sittaung River basin, Kyan Hone River	WI	biv_155_11	Hap116	KX865914	KX865671	KX865785	Present study
Contradens sp.5	Myanmar: Salween River basin, Lake Inle	WI	biv_114_1	Hap050	KX865915	KX865672	KX865786	Present study
Contradens sp.5	Myanmar: Salween River basin, Lake Inle	WI	biv_114_3	Hap050	KX865916	KX865673	KX865787	Present study
Contradens sp.5	Myanmar: Salween River basin, Snake Stream	WI	biv_143_2	Hap050	KX865917	KX865674	KX865788	Present study
Contradens sp.5	Myanmar: Salween River basin, Lake Inle	WI	biv_114_2	Hap051	KX865918	KX865675	KX865789	Present study
Contradens sp.5	Myanmar: Salween River basin, Lake Inle	WI	biv_115_1	Hap050	KX865919	n/a	KX865790	Present study
Contradens sp.5	Myanmar: Salween River basin, Lake Inle	WI	biv_115_3	Hap050	KX865920	n/a	KX865791	Present study
Contradens sp.5	Myanmar: Salween River basin, Lake Inle	WI	biv_115_2	Hap082	KX865921	n/a	KX865792	Present study
Contradens sp.5	Myanmar: Salween River basin, Snake Stream	WI	biv_139_7	Hap117	KX865922	KX865676	KX865793	Present study
Contradens sp.5	Myanmar: Salween River basin, Snake Stream	WI	biv_139_15	Hap118	KX865923	KX865677	KX865794	Present study
Contradens sp.5	Myanmar: Salween River basin, Snake Stream	WI	biv_139_18	Hap119	KX865924	KX865678	KX865795	Present study
Contradens sp.5	Myanmar: Salween River basin, Nam Pilu River	WI	biv_140_22	Hap120	KX865925	KX865679	KX865796	Present study
Contradens sp.5	Myanmar: Salween River basin, Nam Pilu River	WI	biv_140_24	Hap121	KX865926	KX865680	KX865797	Present study
Contradens sp.5	Myanmar: Salween River basin, Nam Pilu River	WI	biv_140_25	Hap50	KX865927	KX865681	KX865798	Present study
Contradens sp.6	Thailand: Mekong River basin, Loei River	PM	biv_119_5	Hap061	KX865928	KX865682	KX865799	Present study
Contradens sp.6	Thailand: Mekong River basin, Loei River	PM	biv_119_6	Hap061	KX865929	KX865683	KX865800	Present study
Contradens sp.6	Laos: Mekong River basin, Nam Long River	PM	biv_182_3	Hap111	KX865930	KX865684	KX865801	Present study
Contradens sp.6	Laos: Mekong River basin, Nam Long River	PM	biv_182_12	Hap111	KX865931	KX865685	KX865802	Present study
Contradens sp.6	Laos: Mekong River basin, Nam Long River	PM	biv_182_10	Hap111	KX865932	n/a	KX865803	Present study
Contradens sp.6	Laos: Mekong River basin, Nam Pe River	PM	biv_184_1	Hap061	KX865933	n/a	KX865804	Present study
Contradens sp.6	Laos: Mekong River basin, Nam Pe River	PM	biv_184_3	Hap061	KX865934	n/a	KX865805	Present study

Putative species (MOTU)	Locality	Range Code	Specimen Voucher	Haplotype Code	COI	16S rRNA	28S rDNA	Reference
Contradens sp.6	Laos: Mekong River basin, Nam Pe River	PM	biv_185_2	Hap061	KX865935	n/a	KX865806	Present study
Physunio cf. eximius (Lea, 1856)	Thailand: Mekong River basin, Chi River	PM	biv_127 _1	Hap095	KX865936	n/a	KX865807	Present study
Physunio cf. eximius (Lea, 1856)	Thailand: Mekong River basin, Chi River	PM	biv_127 _2	Hap096	KX865937	n/a	KX865808	Present study
Physunio cf. eximius (Lea, 1856)	Thailand: Mekong River basin, Chi River	PM	biv_127 _3	Hap095	KX865938	KX865686	KX865809	Present study
Physunio cf. eximius (Lea, 1856)	Thailand: Mekong River basin, Phong River	PM	biv_121 _1	Hap103	KX865939	KX865687	KX865810	Present study
Physunio cf. eximius (Lea, 1856)	Thailand: Mekong River basin, Phong River	PM	biv_121_2	Hap095	KX865940	KX865688	KX865811	Present study
Physunio cf. eximius (Lea, 1856)	Thailand: Mekong River basin, Phong River	PM	biv_121_3	Hap096	KX865941	KX865689	KX865812	Present study
Physunio superbus (Lea, 1843)	Malaysia	PM	X180	Hap221	KX051282	n/a	n/a	Ref. 41
Physunio superbus (Lea, 1843)	Malaysia	PM	BIV1699	Hap221	KX051280	n/a	n/a	Ref. 41
Physunio superbus (Lea, 1843)	Malaysia	PM	X236	Hap221	KX051277	n/a	n/a	Ref. 41
Physunio superbus (Lea, 1843)	Malaysia	PM	X239	Hap221	KX051276	n/a	n/a	Ref. 41
Physunio superbus (Lea, 1843)	Malaysia	PM	BIV1776	Hap222	KX051278	n/a	n/a	Ref. 41
Physunio superbus (Lea, 1843)	Malaysia	PM	BIV1698	Hap222	KX051281	n/a	n/a	Ref. 41
Physunio superbus (Lea, 1843)	Malaysia	PM	BIV1756	Hap222	KX051279	n/a	n/a	Ref. 41
Physunio superbus (Lea, 1843)	Malaysia	PM	X246	Hap223	KX051275	n/a	n/a	Ref. 41
Contradens contradens (Lea, 1838)	West Malaysia: Pahang River	PM	ANSP:389059	Hap184	DQ191411	n/a	AF400692	Ref. 96, 97
Contradens contradens (Lea, 1838)	Malaysia	PM	X076	Hap215	KX051294	n/a	n/a	Ref. 41
Contradens contradens (Lea, 1838)	Malaysia	PM	BIV1787	Hap229	KX051270	n/a	n/a	Ref. 41
Contradens contradens (Lea, 1838)	Malaysia	PM	BIV1789	Hap229	KX051269	n/a	n/a	Ref. 41
Contradens contradens (Lea, 1838)	Malaysia	PM	BIV1765	Hap229	KX051265	n/a	n/a	Ref. 41
Contradens contradens (Lea, 1838)	Malaysia	PM	BIV1638	Hap229	KX051259	n/a	n/a	Ref. 41
Contradens contradens (Lea, 1838)	Malaysia	PM	X054	Hap229	KX051251	n/a	n/a	Ref. 41
Contradens contradens (Lea, 1838)	Malaysia	PM	X060	Hap229	KX051250	n/a	n/a	Ref. 41
Contradens contradens (Lea, 1838)	Malaysia	PM	X072	Hap229	KX051247	n/a	n/a	Ref. 41
Contradens contradens (Lea, 1838)	Malaysia	PM	BIV1767	Hap230	KX051253	n/a	n/a	Ref. 41
Contradens contradens (Lea, 1838)	Malaysia	PM	BIV1695	Hap230	KX051249	n/a	n/a	Ref. 41
Contradens contradens (Lea, 1838)	Malaysia	PM	X132	Hap231	KX051257	n/a	n/a	Ref. 41
Contradens contradens (Lea, 1838)	Malaysia	PM	X100	Hap231	KX051246	n/a	n/a	Ref. 41
Contradens contradens (Lea, 1838)	Malaysia	PM	X208	Hap232	KX051258	n/a	n/a	Ref. 41
Contradens contradens (Lea, 1838)	Malaysia	PM	BIV1764	Hap232	KX051254	n/a	n/a	Ref. 41
Contradens contradens (Lea, 1838)	Malaysia	PM	BIV1777	Hap233	KX051262	n/a	n/a	Ref. 41
Contradens contradens (Lea, 1838)	Malaysia	PM	BIV1748	Hap233	KX051244	n/a	n/a	Ref. 41

Putative species (MOTU)	Locality	Range Code	Specimen Voucher	Haplotype Code	COI	16S rRNA	28S rDNA	Reference
Contradens contradens (Lea, 1838)	Malaysia	PM	BIV1709	Hap234	KX051267	n/a	n/a	Ref. 41
Contradens contradens (Lea, 1838)	Malaysia	PM	BIV1757	Hap234	KX051243	n/a	n/a	Ref. 41
Contradens contradens (Lea, 1838)	Malaysia	PM	BIV1710	Hap235	KX051268	n/a	n/a	Ref. 41
Contradens contradens (Lea, 1838)	Malaysia	PM	BIV1839	Hap236	KX051266	n/a	n/a	Ref. 41
Contradens contradens (Lea, 1838)	Malaysia	PM	BIV1639	Hap237	KX051264	n/a	n/a	Ref. 41
Contradens contradens (Lea, 1838)	Malaysia	PM	X231	Hap238	KX051261	n/a	n/a	Ref. 41
Contradens contradens (Lea, 1838)	Malaysia	PM	BIV1714	Hap239	KX051256	n/a	n/a	Ref. 41
Contradens contradens (Lea, 1838)	Malaysia	PM	BIV1758	Hap240	KX051255	n/a	n/a	Ref. 41
Contradens contradens (Lea, 1838)	Malaysia	PM	X43	Hap241	KX051252	n/a	n/a	Ref. 41
Contradens aff. contradens (Lea, 1838) sp.1	Cambodia: Mekong River basin	PM	UMMZ:304652	Hap183	KP795034	KP795054	KP795016	Ref. 40
Contradens sp.7	Laos: Mekong River basin, a tributary of Nam Fa River near Vieng Phou Kha	PM	biv202_2	Hap188	KY561630	KY561645	KY561662	Present study
Contradens sp.7	Laos: Mekong River basin, a tributary of Nam Fa River near Vieng Phou Kha	PM	biv203_5	Hap188	KY561631	KY561646	KY561663	Present study
Contradens sp.7	Laos: Mekong River basin, a tributary of Nam Fa River near Vieng Phou Kha	PM	biv203_4	Hap189	KY561632	KY561647	KY561664	Present study
Ensidens aff. sagittarius (Lea, 1856) sp.1	Cambodia: Mekong River basin	PM	UMMZ:304651	Hap182	KP795033	KP795053	KP795015	Ref. 40
Ensidens aff. sagittarius (Lea, 1856) sp.2	Thailand: Mekong River basin, artificial pond near the Ban Nong-Bua village	PM	biv_117_1	Hap068	KX865942	KX865690	KX865813	Present study
Ensidens aff. sagittarius (Lea, 1856) sp.2	Thailand: Mekong River basin, artificial pond near the Ban Nong-Bua village	PM	biv_117_2	Hap068	KX865943	KX865691	KX865814	Present study
Ensidens aff. sagittarius (Lea, 1856) sp.2	Thailand: Mekong River basin, artificial pond near the Ban Nong-Bua village	PM	biv_117_3	Hap069	KX865944	KX865692	KX865815	Present study
Ensidens aff. sagittarius (Lea, 1856) sp.3	Thailand: Mekong River basin, Chi River	PM	biv_128_1	Hap073	KX865945	KX865693	KX865816	Present study
Ensidens aff. sagittarius (Lea, 1856) sp.3	Thailand: Mekong River basin, Chi River	PM	biv_128_2	Hap074	KX865946	KX865694	KX865817	Present study
Ensidens aff. sagittarius (Lea, 1856) sp.3	Thailand: Mekong River basin, Chi River	PM	biv_128_3	Hap075	KX865947	n/a	KX865818	Present study
Ensidens aff. sagittarius (Lea, 1856) sp.3	Thailand: Mekong River basin, Chi River	PM	biv_123_1	Hap083	KX865948	n/a	KX865819	Present study
Ensidens aff. sagittarius (Lea, 1856) sp.3	Thailand: Mekong River basin, Chi River	PM	biv_123_2	Hap084	KX865949	KX865695	KX865820	Present study
Ensidens aff. sagittarius (Lea, 1856) sp.3	Thailand: Mekong River basin, Chi River	PM	biv_123_3	Hap085	KX865950	KX865696	KX865821	Present study
Ensidens cf. ingallsianus (Lea, 1852)	Laos: Mekong River basin	PM	NCSM84889	Hap193	KX822641	n/a	KX822598	Ref. 27
Ensidens sp.1	Laos: Mekong River basin	PM	NCSM84902	Hap194	KX822642	n/a	KX822599	Ref. 27
Hyriopsis sp.2	Thailand: Mekong River basin, Chi River	PM	biv_130_1	Hap076	KX865951	KX865697	KX865822	Present study
Hyriopsis sp.2	Thailand: Mekong River basin, Chi River	PM	biv_130_2	Hap077	KX865952	KX865698	KX865823	Present study
Hyriopsis sp.2	Thailand: Mekong River basin, Chi River	PM	biv_130_3	Hap078	KX865953	n/a	KX865824	Present study

Putative species (MOTU)	Locality	Range Code	Specimen Voucher	Haplotype Code	COI	16S rRNA	28S rDNA	Reference
Hyriopsis sp.2	Thailand: Mekong River basin	PM	n/a	Hap195	KX822643	n/a	KX822600	Ref. 27
Hyriopsis sp.2	Thailand: Mekong River basin	PM	Hb5	Hap195	KX383941	n/a	n/a	Ref. 41
Hyriopsis sp.2	Thailand: Mekong River basin	PM	Hb4	Hap195	KX383940	n/a	n/a	Ref. 41
Hyriopsis sp.2	Thailand: Mekong River basin	PM	Hb3	Hap195	KX383939	n/a	n/a	Ref. 41
Hyriopsis sp.1	Thailand: Chao Phraya basin	PM	839512HbB	Hap226	KX383948	n/a	n/a	Ref. 41
Hyriopsis sp.1	Thailand: Chao Phraya basin	PM	839511HbA	Hap227	KX383947	n/a	n/a	Ref. 41
Hyriopsis bialata Simpson, 1900	Malaysia	PM	BIV1774	Hap224	KX051274	n/a	n/a	Ref. 41
Hyriopsis bialata Simpson, 1900	Malaysia	PM	BIV1775	Hap224	KX051273	n/a	n/a	Ref. 41
Hyriopsis myersiana (Lea, 1856)	Thailand	PM	n/a	Hap197	KX822645	n/a	KX822602	Ref. 27
Hyriopsis myersiana (Lea, 1856)	Thailand: Mekong River basin	PM	Biv40	Hap197	KX383943	n/a	n/a	Ref. 41
Hyriopsis myersiana (Lea, 1856)	Thailand: Mekong River basin	PM	COI17	Hap225	KX383949	n/a	n/a	Ref. 41
Hyriopsis desowitzi Brandt, 1974	Thailand	PM	n/a	Hap196	KX822644	n/a	KX822601	Ref. 27
Hyriopsis desowitzi Brandt, 1974	Thailand: Chao Phraya basin	PM	Hd11	Hap196	KX383945	n/a	n/a	Ref. 41
Hyriopsis desowitzi Brandt, 1974	Thailand: Chao Phraya basin	PM	Hd10	Hap196	KX383944	n/a	n/a	Ref. 41
Hyriopsis desowitzi Brandt, 1974	Thailand: Chao Phraya basin	PM	n/a	Hap228	KX383946	n/a	n/a	Ref. 41
Rectidens sumatrensis (Dunker, 1852)	Malaysia	PM	n/a	Hap203	KX822664	n/a	KX822620	Ref. 27
**UNIONINAE Rafinesque, 1820**								
Unio crassus Philipsson, 1788*	France	EU	n/a	hap260	KC703878	n/a	KC703644	Ref. 78
Unio pictorum (Linnaeus, 1758)	Europe	EU	n/a	hap261	KC429109	n/a	KC429447	Ref. 98
Unio tumidus Philipsson, 1788	Ukraine	EU	n/a	hap262	KX822672	n/a	KX822630	Ref. 27
Aculamprotula tortuosa (Lea, 1865)	China	EA	n/a	hap263	KX822631	n/a	KX822586	Ref. 27
Cuneopsis heudei (Heude, 1874)	China	EA	n/a	hap264	KX822638	n/a	KX822595	Ref. 27
Cuneopsis pisciculus (Heude, 1874)	China	EA	n/a	hap265	KX822639	n/a	KX822596	Ref. 27
Cuneopsis rufescens (Heude, 1874)	China	EA	n/a	hap266	KX822640	n/a	KX822597	Ref. 27
Nodularia douglasiae (Griffith & Pidgeon, 1833)	China	EA	n/a	hap267	KX822653	n/a	KX822610	Ref. 27
Nodularia nuxpersicae Dunker, 1848	Vietnam	EA	NCSM84990	hap268	KX822654	n/a	KX822611	Ref. 27
Schistodesmus lampreyanus (Baird & Adams, 1867)	China	EA	n/a	hap269	KX822665	n/a	KX822621	Ref. 27
**ANODONTINAE Rafinesque, 1820**								
Alasmidonta marginata Say, 1818	USA	NA	UMMZ 265695	hap242	AF156502	n/a	AF400688	Ref. 96
Anodonta anatina (Linnaeus, 1758)	European Russia	EU	n/a	hap243	KX822632	n/a	KX822588	Ref. 27

Putative species (MOTU)	Locality	Range Code	Specimen Voucher	Haplotype Code	COI	16S rRNA	28S rDNA	Reference
Anodonta cygnea (Linnaeus, 1758)	Italy	EU	n/a	hap244	KX822633	n/a	KX822589	Ref. 27
Anodonta nuttalliana Lea, 1838	USA	NA	n/a	hap245	KX822634	n/a	KX822590	Ref. 27
Lasmigona compressa (Lea, 1829)	USA	NA	UMMZ 265696	hap246	AF156503	n/a	DQ191414	Ref. 97, 99
Pseudanodonta complanata (Rossmässler, 1835)	Ukraine	EU	n/a	hap247	KX822661	n/a	KX822617	Ref. 27
Pyganodon grandis (Say, 1829)*	USA	NA	n/a	hap248	AF231734	n/a	AF305384	Ref. 99, 100
Simpsonaias ambigua (Say, 1825)	USA	NA	NCSM30607	hap249	KX822666	n/a	KX822622	Ref. 27
Strophitus undulatus (Say, 1817)*	USA	NA	UMMZ 265693	hap250	AF156505	n/a	DQ191415	Ref. 97, 99
Anemina sp.	Siberia	EA	n/a	hap251	KY561633	KY561648	KY561665	Present study
Cristaria plicata (Leach, 1814)	Vietnam	EA	n/a	hap252	KY561634	n/a	KY561666	Present study
Cristaria sp.	China	EA	n/a	hap253	EU698940	n/a	n/a	GenBank
Pletholophus tenuis (Griffith & Pidgeon, 1833)	Vietnam	EA	NCSM84924	hap254	KX822658	n/a	KX822614	Ref. 27
Sinanodonta lucida (Heude, 1877)	China	EA	n/a	hap255	KX822667	n/a	KX822624	Ref. 27
Sinanodonta sp.	Vietnam	EA	n/a	hap256	KY561635	KY561649	KY561667	Present study
Lanceolaria gladiola (Heude, 1877)	China	EA	n/a	hap257	KX822648	n/a	KX822605	Ref. 27
Lanceolaria grayana (Lea, 1834)	China	EA	n/a	hap258	KX822649	n/a	KX822606	Ref. 27
Lanceolaria grayii (Griffith & Pidgeon, 1833)	Vietnam	EA	n/a	hap259	KX822650	n/a	KX822607	Ref. 27
**GONIDEINAE Ortmann, 1916**								
Chamberlainia hainesiana (Lea, 1856)	Thailand	PM	n/a	hap270	KX822635	n/a	KX822592	Ref. 27
Sinohyriopsis cumingii (Lea, 1852)*	Vietnam	EA	n/a	hap271	KY561636	KY561650	KY561668	Present study
Lamprotula caveata (Heude, 1877)	China	EA	n/a	hap272	KX822646	n/a	KX822603	Ref. 27
Lamprotula leaii (Griffith & Pidgeon, 1833)	Vietnam	EA	n/a	hap273	KY561637	KY561651	KY561669	Present study
Potomida littoralis (Cuvier, 1798)	France	EU	n/a	hap274	JN243905	n/a	JN243883	Ref. 37
Pronodularia japanensis (Lea, 1859)*	Japan	EA	NCSM27183	hap275	KX822659	KU946322	KX822615	Ref. 27, 101
Gonidea angulata (Lea, 1838)*	USA	NA	n/a	hap276	DQ272371	n/a	AF400691	Ref. 96, 102
Leguminaia wheatleyi (Lea, 1862)	Turkey	EU	n/a	hap277	KX822651	n/a	KX822608	Ref. 27
Leguminaia sp.	Turkey	EU	n/a	hap278	KY561638	KY561652	n/a	Present study
Microcondylaea bonellii (A. Ferussac 1827)	Italy	EU	n/a	hap279	KX822652	n/a	KX822609	Ref. 27
Solenaia carinata (Heude, 1877)	China	EA	n/a	hap280	KX822669	n/a	KX822626	Ref. 27
Solenaia oleivora (Heude, 1877)	China	EA	n/a	hap281	KX822670	n/a	KX822627	Ref. 27
Solenaia sp.	Vietnam	EA	n/a	hap282	KY561639	KY561653	KY561670	Present study

Putative species (MOTU)	Locality	Range Code	Specimen Voucher	Haplotype Code	COI	16S rRNA	28S rDNA	Reference
**AMBLEMINAE Rafinesque, 1820**								
Amblema plicata (Say, 1817)	USA	NA	n/a	hap283	U56841	n/a	AF305385	Ref. 96, 103
Actinonaias ligamentina (Lamarck, 1819)	USA	NA	n/a	hap284	AF156517	n/a	DQ191420	Ref. 97, 99
Lampsilis cardium Rafinesque, 1820*	USA	NA	n/a	hap285	AF120653	n/a	AF305386	Ref. 96, 104
Villosa iris (Lea, 1829)	USA	NA	n/a	hap286	AF156524	n/a	DQ191422	Ref. 97, 99
Elliptio complanata (Lightfoot, 1786)*	USA	NA	n/a	hap287	EU448173	n/a	JF899181	Ref. 27, 105
Elliptio dilatata (Rafinesque, 1820)*	USA	NA	n/a	hap288	AF156507	n/a	AF400690	Ref. 96, 99
Pleurobema sintoxia (Rafinesque, 1820)	USA	NA	n/a	hap289	AF156509	n/a	DQ191418	Ref. 97, 99
Quadrula quadrula (Rafinesque, 1820)	USA	NA	n/a	hap290	AF156511	n/a	DQ191417	Ref. 97, 99
Quadrula verrucosa (Rafinesque, 1820)	USA	NA	n/a	hap291	DQ191413	n/a	DQ191416	Ref. 97
**Out-Group Taxa**								
**MARGARITIFERIDAE Haas, 1940**								
Margaritifera laosensis (Lea, 1863)	Laos: Mekong River basin, Nam Long River	not used	biv 186_1	Hap030	JX497731	KC845943	KT343741	Ref. 36
Margaritifera dahurica (Middendorff, 1850)	Far East of Russia: Amur River basin, Ilistaya River	not used	biv 92_6	Hap031	KJ161516	KJ943526	KT343747	Ref. 36
Margaritifera margaritifera (Linnaeus, 1758)	Northwestern Russia: Onega River basin, Somba River	not used	biv 618	Hap026	KX550089	KX550091	KX550093	Present study
Margaritifera laevis (Haas, 1910)	Far East of Russia: Kurile Archipelago, Kunashir Island, Sennaya River	not used	biv d0036/22	Hap024	KJ161500	KJ943523	KT343742	Ref. 36
Margaritifera middendorffi (Rosén, 1926)	Far East of Russia: Kamchatka, Bolshaya River basin, Nachilova River	not used	biv d0099/6	Hap025	KJ161547	KJ943528	KT343745	Ref. 36
Margaritifera falcata (Gould, 1850)	USA: Idaho, Fremont Co., Buffalo River	not used	n/a	Hap027	AY579128	AY579085	n/a	Ref. 106
Margaritifera auricularia (Spengler, 1793)	Spain: Tarragona, Ebro River	not used	n/a	Hap010	AY579125	AY579083	n/a	Ref. 106
Margaritifera marocana (Pallary, 1918)	Morocco: Oum Er-Rbia River basin, El-Abid River	not used	n/a	Hap011	EU429679	EU429691	n/a	Ref. 107
Margaritifera monodonta (Say, 1829)	USA: Mississippi River basin	not used	n/a	Hap028	AY579131	AY579089	AF305382	Ref. 96, 106
Margaritifera marrianae Johnson, 1983*	USA: Alabama River basin	not used	n/a	Hap029	HM849098	AY579086	n/a	Ref. 106, 108
**IRIDINIDAE Swainson, 1840**								
Aspatharia pfeifferiana (Bernardi,	Zambia: Chambeshi River	not	n/a	Hap009	KC429107	KC429264	n/a	Ref. 98, 109

Putative species (MOTU)	Locality	Range Code	Specimen Voucher	Haplotype Code	COI	16S rRNA	28S rDNA	Reference
1860)		used						
Chambardia wahlbergi (Krauss, 1848)*	Zambia: Zambezi River	not used	n/a	Hap012	JN243886	KP184845	JN243864	Ref. 47
**ETHERIIDAE Deshayes, 1832**								
Etheria elliptica Lamarck, 1807	Zambia: Chambeshi River	not used	n/a	Hap014	KP184897	KP184847	KP184873	Ref. 47
**MYCETOPODIDAE Gray, 1840**		not used						
Anodontites elongata (Swainson, 1823)	Peru	not used	n/a	Hap013	KP184896	KP184846	KP184872	Ref. 47
**HYRIIDAE Swainson, 1840**								
Triplodon corrugatus (Lamarck, 1819)	Peru	not used	n/a	Hap015	JN243890	KP184851	JN243868	Ref. 47
Castalia ambigua Lamarck, 1819	Peru	not used	n/a	Hap016	JN243889	KP184848	JN243867	Ref. 47
Diplodon suavidicus (Lea, 1856)	Guyana	not used	n/a	Hap017	KP184898	KP184849	KP184874	Ref. 47
Microdontia anodontaeformis (Tapparone Canefri, 1883)	New Guinea	not used	n/a	Hap018	KP184909	KP184861	KP184885	Ref. 47
Alathyria jacksoni Iredale, 1934	Australia: New South Wales	not used	n/a	Hap019	KP184912	KP184864	KP184888	Ref. 47
Alathyria pertexta Iredale, 1934	Australia: Queensland	not used	n/a	Hap020	KP184910	KP184862	KP184886	Ref. 47
Alathyria profuga (Gould, 1850)	Australia: New South Wales	not used	n/a	Hap021	KP184913	KP184865	KP184889	Ref. 47
Lortiella froggatti Iredale, 1934*	Western Australia	not used	n/a	Hap022	AF231746	KP184867	KP184891	Ref. 47
Velesunio ambiguus (Philippi, 1847)*	Australia: New South Wales	not used	n/a	Hap023	KP184915	KP184868	KP184892	Ref. 47, 98
**TRIGONIIDAE Lamarck, 1819**								
Neotrigonia margaritacea (Lamarck, 1804)*	Tasmania and Australia	not used	n/a	Hap001	U56850	DQ280034	DQ279963	Ref. 103, 106, 110
Neotrigonia lamarckii (Gray, 1838)	Australia: Coral Sea, North Stradbroke Island, Queensland	not used	n/a	Hap002	KC429105	KC429262	KC429443	Ref. 98, 109

Molecular Operational Taxonomic Units (MOTUs) are separated using the Bayesian PTP model (see Methods). Prospective biological species were delineated based on the MOTU approach with the supplement of genetic distance analyses, nuclear gene phylogeny and morphological analyses. *Chimeric taxa (sequences were obtained from different specimens). **Sequences that revealed doubtful placement in the COI phylogeny, but the analysis of nuclear 28S rDNA sequences supports the status of both taxa (see Materials and Methods section). ***Prospective species-level units that were delineated solely based on the MOTUs approach, because other data (nuclear gene sequences and specimen vouchers) were not available.

Supplementary Table 2. Results of Repeatability Clade Analysis (RCA) of main clades corresponding to the preferred topology

Analyses and clades	Combined dataset		mtDNA				28S nDNA
	3 codons of COI + 16S + 28S*	COI + 16S + 28S	3 codons of COI + 16S	COI + 16S	COI (3 codons)	COI	
BI (BPP, %)							
Parreysiinae	**100**	**100**	**100**	**100**	83	**100**	**100**
Pseudodontinae	**100**	**100**	**100**	**96**	82	**96**	**100**
Pseudodontini (Paleo-Mekong radiation I)	**100**	**100**	-	58	-	58	**100**
Rectidentinae	**100**	**100**	**100**	**100**	**100**	**100**	**100**
Rectidentini (Paleo-Mekong radiation II)	**100**	**100**	**100**	**100**	**100**	**100**	-
Contradentini	**100**	**100**	**100**	**100**	**100**	**100**	**100**
(Unioninae + Anodontinae) + (Gonideinae + Ambleminae)	54	**100**	-	-	-	-	76
(Unioninae + Anodontinae)	**100**	**100**	**100**	**100**	**100**	**100**	**100**
Unioninae	89	-	**99**	81	**99**	81	-
Anodontinae	**99**	92	**99**	-	**98**	-	-
(Gonideinae + Ambleminae)	**100**	**99**	-	-	-	-	-
Gonideinae	74	-	88	**99**	-	-	-
Ambleminae	**100**	**100**	**100**	**100**	**100**	**100**	-
ML (BS, %)							
Parreysiinae	**100**	**100**	**93**	**95**	60	68	**98**
Pseudodontinae	**99**	**99**	**88**	**86**	56	59	**89**
Pseudodontini (Paleo-Mekong radiation I)	**95**	**93**	38	40	-	-	**90**
Rectidentinae	**100**	**100**	**94**	**95**	**77**	**87**	**98**
Rectidentini (Paleo-Mekong radiation II)	**99**	**99**	**98**	**96**	**84**	**92**	-
Contradentini	**100**	**100**	**90**	**97**	**82**	**92**	**99**
(Unioninae + Anodontinae) + (Gonideinae + Ambleminae)	62	57	-	-	-	-	31
(Unioninae + Anodontinae)	**100**	**100**	**82**	**84**	**83**	77	**94**
Unioninae	58	65	72	72	71	66	-
Anodontinae	60	66	41	55	-	48	-
(Gonideinae + Ambleminae)	50	46	13	-	-	-	-
Gonideinae	-	23	31	41	-	-	-
Ambleminae	**98**	**95**	**97**	**96**	**95**	**95**	13

*Preferred topology. "-" Topological difference (lack of a clade in the optimal topology). High support values (BPP ≥95%, BS ≥75%) are highlighted in bold.

Supplementary Table 3. Diversification rate statistics for each endemic Indo-Chinese clade of the Unionidae

**Clade**	**Paradis's test of diversification with three survival models**[95]				**The constant-rates test**[94]	
	**Div. rate (delta ± s.e.)**	**LRT *p*-value (constant rate model vs. Weibull model)**	**Beta parameter of the Weibull model (± s.e.)**	**Selected model (by AIC)**	**Gamma statistic**	***p*-value (two-sided)**
Rectidentinae	0.037±0.008	0.013*	1.603±0.256	Weibull (variable rate through time)	-1.612	0.107
Mekong's Rectidentini	0.036±0.011	0.018*	2.744±0.573	Weibull (variable rate through time)	-2.306	0.021*
Contradentini	0.047±0.014	0.102	1.520±0.345	Constant rate	-0.747	0.455
Mekong's Pseudodontinae	0.038±0.010	0.022*	1.683±0.324	Weibull (variable rate through time)	-1.448	0.148

*A variable diversification rate.

Supplementary Table 4. List of new sampling localities in Indo-China. *N* is the number of sequenced specimens (total *N* = 153), and *S* is the number of putative biological species (total *S* = 28)

Country	Freshwater Drainage	Sampling Locality	Locality Code	Latitude (N)	Longitude (E)	Alt., m a.s.l.	Habitat	Meso-habitat	*N*	*S*
Laos	Nam Ou River -> Mekong River	Nam Long River	01	21.7700	102.1863	480	Mountain river	Run	5	2
Laos	Nam Ou River -> Mekong River	Nam Pe River	02	21.5905	102.0829	863	Mountain river	Run	4	2
Laos	Nam Fa River -> Mekong River	A tributary of Nam Fa River near Vieng Phou Kha	03	20.6820	101.0794	674	Mountain stream	Pool	7	2
Myanmar	Irrawaddy River	A tributary of Lake Indawgyi	04	25.1209	96.2812	174	Plain stream	Pool	3	1
Myanmar	Irrawaddy River	Lake Indawgyi	05	25.1099	96.2925	170	Lake	Pool	12	3
Myanmar	Mali Hka River -> Irrawaddy River	Mansakun River	06	27.4909	97.3351	413	Mountain river	Run	3	1
Myanmar	Mali Hka River -> Irrawaddy River	Nam Balak River	07	27.4741	97.3493	418	Mountain river	Run	4	2
Myanmar	Mali Hka River -> Irrawaddy River	Nam Shu River	08	27.5482	97.3700	434	Mountain river	Run	3	1
Myanmar	Mali Hka River -> Irrawaddy River	Pan Khai Stream	09	27.4493	97.3432	209	Mountain stream	Pool	4	2
Myanmar	Mali Hka River -> Irrawaddy River	Unnamed stream	10	27.5475	97.3705	435	Mountain stream	Run	2	1
Myanmar	Irrawaddy River	Nanuinhka Chaung River	11	25.0815	96.2874	178	Plain stream	Run	3	1
Myanmar	Kaladan River	Unnamed puddle	12	21.0078	92.9831	14	Puddle	Pool	1	1
Myanmar	Salween River	Lake Inle	13	20.4420	96.9036	887	Lake	Pool	9	2
Myanmar	Salween River	Lake Inle, a channel in Nuangshve	14	20.6632	96.9310	892	Lake	Pool	3	1
Myanmar	Salween River	Nam Pilu River	15	19.6746	97.1352	878	Plain river	Pool	6	2
Myanmar	Salween River	Snake Stream	16	19.7266	97.0992	878	Plain stream	Pool	5	2
Myanmar	Sittaung River	Kyan Hone River	17	19.5059	96.8280	896	Mountain river	Run	6	1
Myanmar	Sittaung River	Tauk Ue Kupt River	18	19.3075	96.7219	426	Mountain river	Run	3	1
Myanmar	Tavoy River	Tavoy River	19	14.5012	98.1557	18	Plain river	Run	13	3
Thailand	Mekong River	A pond near the Ban Nong-Bua village	20	17.4547	101.3958	650	Pond	Pool	6	2
Thailand	Mekong River	Chi River	21	16.2258	103.3007	145	Plain river	Run	27	7
Thailand	Mekong River	Huai Nam Khu Reservoir	22	17.1050	101.6193	288	Reservoir	Pool	1	1
Thailand	Mekong River	Loei River	23	17.0982	101.4814	531	Mountain river	Run	6	2
Thailand	Mekong River	Phong River	24	16.8616	101.9105	242	Plain river	Run	17	4

Supplementary Table 5. List of sequenced specimens for each prospective biological species of the Unionidae from the Oriental Region over corresponding freshwater basins. Freshwater basins are as follows: (ME) Mekong, (CP) Chao Phraya, (MA) Malay Peninsula, (IR) Irrawaddy, (SW) Salween, (ST) Sittaung, (TA) Tavoy, (KA) Kaladan, and (IN) Indian rivers. Each value in the table represents the number of sequenced specimens (see Supplementary Table 1 and 4 for details)

**Subfamily/Species**	**Paleo-Mekong basin**			**Western Indo-China**					**IN**	**Total**
	**ME**	**CP**	**MA**	**IR**	**SW**	**ST**	**TA**	**KA**		
PARREYSIINAE										
Lamellidens aff. *corrianus* (Lea, 1834) sp.1	-	-	-	1	-	-	-	-	-	1
Lamellidens aff. *corrianus* (Lea, 1834) sp.2	-	-	-	-	-	-	-	-	1	1
Lamellidens aff. *marginalis* (Lamarck, 1819) sp.1	-	-	-	-	-	-	-	-	1	1
Lamellidens aff. *marginalis* (Lamarck, 1819) sp.2	-	-	-	-	-	-	-	-	1	1
Lamellidens aff. *marginalis* (Lamarck, 1819) sp.3	-	-	-	-	-	-	-	1	-	1
Lamellidens aff. *marginalis* (Lamarck, 1819) sp.4	-	-	-	-	-	-	-	-	1	1
Lamellidens aff. *marginalis* (Lamarck, 1819) sp.5	-	-	-	-	-	-	-	-	1	1
Lamellidens exolescens (Gould, 1843)	-	-	-	-	-	-	6	-	-	6
Lamellidens indawgyiensis Prashad, 1930	-	-	-	4	-	-	-	-	-	4
Lamellidens cf. *generosus* (Gould, 1847)	-	-	-	-	10	-	-	-	-	10
Oxynaia cf. *pugio* (Benson, 1862)	-	-	-	1	-	-	-	-	-	1
Parreysia cf. *burmana* (Blanford, 1869)	-	-	-	6	-	-	-	-	-	6
Parreysia cf. *corrugata* (Müller, 1774)	-	-	-	-	-	-	-	-	6	6
Parreysia olivacea Prashad, 1930	-	-	-	1	-	-	-	-	-	1
Parreysia tavoyensis (Gould, 1843)	-	-	-	2	-	-	1	-	-	3
Radiatula aff. *caerulea* (Lea, 1831)	-	-	-	3	-	-	-	-	-	3
Radiatula aff. *humilis* (Lea, 1856) sp.1	6	-	-	-	-	-	-	-	-	6
Radiatula aff. *humilis* (Lea, 1856) sp.2	2	-	-	-	-	-	-	-	-	2
Radiatula aff. *khadakvaslaensis* (Ray, 1966) sp.1	-	-	-	-	-	-	-	-	1	1
Radiatula aff. *khadakvaslaensis* (Ray, 1966) sp.2	-	-	-	-	-	-	-	-	1	1
Radiatula aff. *khadakvaslaensis* (Ray, 1966) sp.3	-	-	-	-	-	-	-	-	1	1
Radiatula cf. *bonneaudii* (Eydoux, 1838)	-	-	-	1	-	-	-	-	-	1
Scabies aff. *crispata* (Gould, 1843)	1	-	-	-	-	-	-	-	-	1
Scabies crispata (Gould, 1843)	-	-	-	-	-	-	6	-	-	6
**Total**	**9**	**0**	**0**	**19**	**10**	**0**	**13**	**1**	**14**	**66**
PSEUDODONTINAE										
Pilsbryoconcha aff. *compressa* (Martens, 1860) sp.1	1	-	-	-	-	-	-	-	-	1
Pilsbryoconcha aff. *exilis* (Lea, 1838) sp.1	1	-	-	-	-	-	-	-	-	1
Pilsbryoconcha aff. *exilis* (Lea, 1838) sp.2	-	-	1	-	-	-	-	-	-	1
Pilsbryoconcha cf. *compressa* (Martens, 1860)	5	-	3	-	-	-	-	-	-	8
Pilsbryoconcha cf. *exilis* (Lea, 1838)	-	-	5	-	-	-	-	-	-	5
Pseudodon aff. *inoscularis* (Gould, 1844) sp.1	1	-	-	-	-	-	-	-	-	1
Pseudodon aff. *mouhotii* (Lea, 1863) sp.1	7	-	-	-	-	-	-	-	-	7
Pseudodon aff. *mouhotii* (Lea, 1863) sp.2	4	-	-	-	-	-	-	-	-	4
Pseudodon aff. *mouhotii* (Lea, 1863) sp.3	2	-	-	-	-	-	-	-	-	2

Subfamily/Species	Paleo-Mekong basin			Western Indo-China					IN	Total
	ME	CP	MA	IR	SW	ST	TA	KA		
Pseudodon aff. *vondembuschianus* (Lea, 1840) sp.1	1	-	-	-	-	-	-	-	-	1
Pseudodon aff. *vondembuschianus* (Lea, 1840) sp.2	1	-	-	-	-	-	-	-	-	1
Pseudodon cambodjensis (Petit de la Saussaye, 1865)	2	-	3	-	-	-	-	-	-	5
Pseudodon cf. *avae* (Theobald, 1873)	-	-	-	3	-	-	-	-	-	3
Pseudodon cf. *ellipticus* Conrad, 1865	10	-	-	-	-	-	-	-	-	10
Pseudodon cf. *inoscularis* (Gould, 1844)	1	-	-	-	-	-	-	-	-	1
Pseudodon cumingii (Lea, 1850)	2	-	-	-	-	-	-	-	-	2
Pseudodon sp.1	-	-	3	-	-	-	-	-	-	3
Pseudodon vondembuschianus (Lea, 1840)	-	-	13	-	-	-	-	-	-	13
**Total**	**38**	**0**	**28**	**3**	**0**	**0**	**0**	**0**	**0**	**69**
RECTIDENTINAE										
Contradens aff. *contradens* (Lea, 1838) sp.1	1	-	-	-	-	-	-	-	-	1
Contradens contradens (Lea, 1838)	-	-	26	-	-	-	-	-	-	26
Contradens sp.1	1	-	-	-	-	-	-	-	-	1
Contradens sp.2	-	-	-	19	-	-	-	-	-	19
Contradens sp.3	-	-	-	-	-	3	-	-	-	3
Contradens sp.4	-	-	-	-	-	6	-	-	-	6
Contradens sp.5	-	-	-	-	13	-	-	-	-	13
Contradens sp.6	8	-	-	-	-	-	-	-	-	8
Contradens sp.7	3	-	-	-	-	-	-	-	-	3
Ensidens aff. *sagittarius* (Lea, 1856) sp.1	1	-	-	-	-	-	-	-	-	1
Ensidens aff. *sagittarius* (Lea, 1856) sp.2	3	-	-	-	-	-	-	-	-	3
Ensidens aff. *sagittarius* (Lea, 1856) sp.3	6	-	-	-	-	-	-	-	-	6
Ensidens cf. *ingallsianus* (Lea, 1852)	1	-	-	-	-	-	-	-	-	1
Ensidens sp.1	1	-	-	-	-	-	-	-	-	1
Hyriopsis bialata Simpson, 1900	-	-	2	-	-	-	-	-	-	2
Hyriopsis desowitzi Brandt, 1974	-	4	-	-	-	-	-	-	-	4
Hyriopsis myersiana (Lea, 1856)	3	-	-	-	-	-	-	-	-	3
Hyriopsis sp.1	-	2	-	-	-	-	-	-	-	2
Hyriopsis sp.2	7	-	-	-	-	-	-	-	-	7
Physunio cf. *eximius* (Lea, 1856)	6	-	-	-	-	-	-	-	-	6
Physunio cf. *modelli* Brandt, 1974	10	-	-	-	-	-	-	-	-	10
Physunio superbus (Lea, 1843)	-	-	8	-	-	-	-	-	-	8
Rectidens sumatrensis (Dunker, 1852)	-	-	1	-	-	-	-	-	-	1
Trapezoideus sp.1	1	-	-	-	-	-	-	-	-	1
**Total**	**52**	**6**	**37**	**19**	**13**	**9**	**0**	**0**	**0**	**136**

Supplementary Table 6. Similarity matrix (Jaccard Similarity Index, %) between freshwater basins of the Oriental Region based on the presence/absence of each prospective biological species of the Unionidae (see Supplementary Table 5 for details). The nonzero values are in bold. Freshwater basins are as follows: (ME) Mekong, (CP) Chao Phraya, (MA) Malay Peninsula, (IR) Irrawaddy, (SW) Salween, (ST) Sittaung, (TA) Tavoy, (KA) Kaladan, and (IN) Indian rivers

Basins	ME	CP	MA	IR	SW	ST	TA	KA
CP	0.00							
MA	**5.26**	0.00						
IR	0.00	0.00	0.00					
SW	0.00	0.00	0.00	0.00				
ST	0.00	0.00	0.00	0.00	0.00			
TA	0.00	0.00	0.00	**8.33**	0.00	0.00		
KA	0.00	0.00	0.00	0.00	0.00	0.00	0.00	
IN	0.00	0.00	0.00	0.00	0.00	0.00	0.00	0.00

Supplementary Table 7. Primer sequences for PCR amplification and sequencing

Gene fragment	Primer's name	Direction	Sequence (5′-3′)	Reference
COI	LoboF1	Forward	kbtchacaaaycayaargayathgg	Ref. 111
	LoboR1	Reverse	taaacytcwggrtgwccraaraayca	
16S rRNA	16Sar	Forward	cgcctgtttatcaaaaacat	Ref. 112
	16sar-L-myt	Forward	cgactgtttaacaaaaacat	Ref. 113
	16sbr-H-myt	Reverse	ccgttctgaactcagctcatgt	
28S rDNA	C1	Forward	acccgctgaatttaagcat	Ref. 114
	D2	Reverse	tccgtgtttcaagacgg	

Supplementary Table 8. Alignment length prior to and after treatment for length variability in GBlocks v. 0.91b

Partition	Original length of alignment (bp)	Fraction selected by GBlocks (%)	Final length of alignment (bp)
COI	659	100	659
16S rRNA	556	78	434
28S rDNA	866	87	755

Supplementary Table 9. Probability (*p*-value) of phylogenetic conflict among sequence data sets from a partition-homogeneity test implemented in PAUP* v. 4.0a150. Significant value is in bold

Sequence data set	16S	28S
COI	1.00	0.49
16S		***0.03***
COI+16S		0.70

Supplementary Table 10. Models of sequence evolution for each partition based on corrected Akaike Information Criterion (AICc) of MEGA6 that were applied within a Bayesian inference framework

Partition	Model	Gamma	Invariant
Indo-China + Africa data set			
COI (1st+2nd+3rd codons)	GTR+G+I	0.92	0.50
1st codon of COI	GTR+G	2.16	n/a
2nd codon of COI	TN93+G+I	0.92	0.51
3rd codon of COI	TN93+I	n/a	0.78
Full data set			
COI (1st+2nd+3rd codons)	GTR+G+I	0.60	0.40
1st codon of COI	GTR+G	1.25	n/a
2nd codon of COI	GTR+G+I	1.15	0.49
3rd codon of COI	GTR+G	0.54	n/a
16S	GTR+G+I	0.40	0.24
28S	GTR+G+I	0.59	0.30

n/a – not available.

Supplementary Table 11. Evaluation of calibration lineages based on the empirical scaling factor (ESF) of Marshall[88]

Calibration No	MRCA	Node-to-tip length relative to base of tree (*NTL_i*)	Age of oldest fossil, Ma (*FA_i*)	Empirical scaling factor (*$si = FA_i / NTL_i$*)	Source of calibration
Calibration 1	*Lamprotula*	0.0634	34.0	536	Present study
Calibration 2	*Cristaria*	0.0500	34.0	680	Present study
Calibration 3	*Cuneopsis*	0.1185	34.0	287	Present study
Calibration 4	*Margaritifera falcata – M. laevis*	0.0898	46.0	512	Ref. 36
Calibration 5	*Margaritifera margaritifera – M. dahurica*	0.0833	34.0	408	Ref. 36
Calibration 6	Margaritiferidae	0.1422	129.4	910	Present study
Calibration 7	Unionidae	0.5616	155.0	276	Ref. 47
Calibration 8	'The core Velesunioninae' (*Velesunio* + *Alathyria* + *Lortiella* + *Microdontia*)	0.1862	99.6	535	Ref. 47
Calibration 9*	Coelaturini (*Nitia*, *Mweruella*, *Coelatura*)	0.1532	7.5	49	Ref. 47
Calibration 10*	*Parreysia*	0.0517	9.0	174	Present study
Calibration 11*	*Lamellidens*	0.0748	9.0	120	Present study

* – These calibrations were not used in BEAST analyses because of low ESF values (<100), which could reveal incomplete fossil records of the lineage[88], see Supplementary Table 13 for details.

Supplementary Table 12. List of fossil calibrations that were not used in the BEAST analyses because of low ESF values (<100), which could reveal incomplete fossil records of the lineage[88]

Calibration no.	MRCA	Description	Reference
Calibration 9	Coelaturini (*Nitia*, *Mweruella*, *Coelatura*)	Absolute age estimate: 7.5 Ma.	Ref. 32: Crown calibration
Calibration 10	*Parreysia*	Hard minimum age: 9 Ma, †*Parreysia binaiensis* Takayasu, Gurung & Matsuoka, 1995. Diagnosis and phylogenetic placement: The assignation of this species to the genus is based on the heavy, inflated and rounded subrhomboidal shell with radial zigzag sculpture on the umbo, and irregular, heavy and striated pseudocardinal teeth[115]. Stratigraphic horizon and locality: Lower member of the Binai Khola Formation, Nepal[115]. Absolute age estimate: lower boundary of the Binai Khola Formation, 9 Ma, based on stratigraphy[115].	Present study: Crown calibration
Calibration 11	*Lamellidens*	Hard minimum age: 9 Ma, †*Lamellidens arungensis* Takayasu, Gurung & Matsuoka, 1995. Diagnosis and phylogenetic placement: The assignation of this species to the genus is based on the elongated elliptical shell, compressed, elongated pseudocardinal teeth and long posterior lamellar teeth[115]. Stratigraphic horizon and locality: Lower member of the Binai Khola Formation, Nepal[115]. Absolute age estimate: lower boundary of the Binai Khola Formation, 9 Ma, based on stratigraphy[115].	Present study: Crown calibration

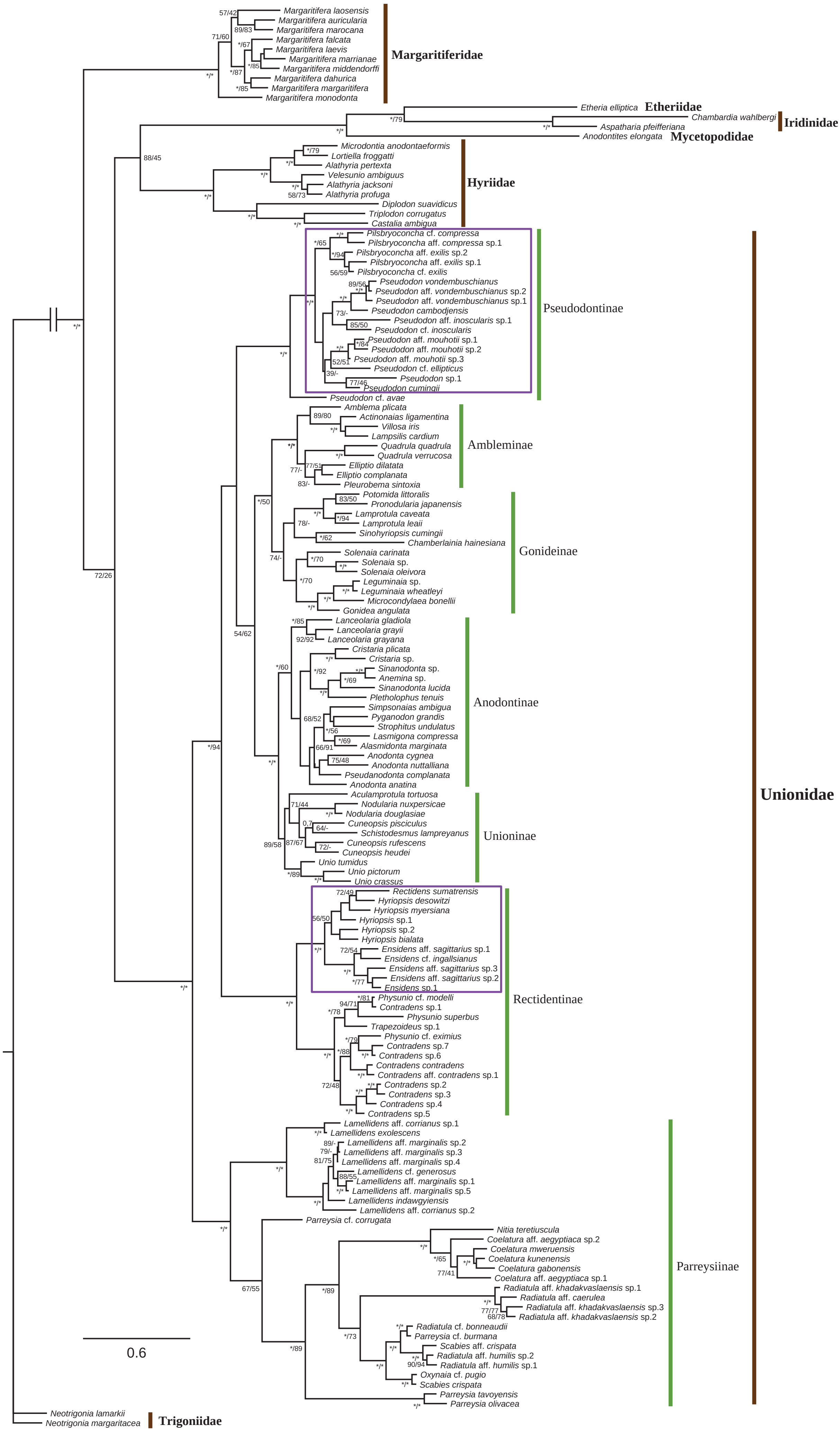

Supplementary Figure 1. Majority rule consensus phylogenetic tree of the Unionidae recovered from BI analysis of the complete data set of mitochondrial and nuclear sequences (five partitions: three codons of COI + 16S rRNA + 28S rDNA). Numbers near branches are the Bayesian posterior probability/ML bootstrap support values (an asterisk indicates value of ≥95%; values <50% are omitted; "-" indicates topological difference). The violet frames indicate the two ancient bivalve radiations within the Paleo-Mekong drainage basin.

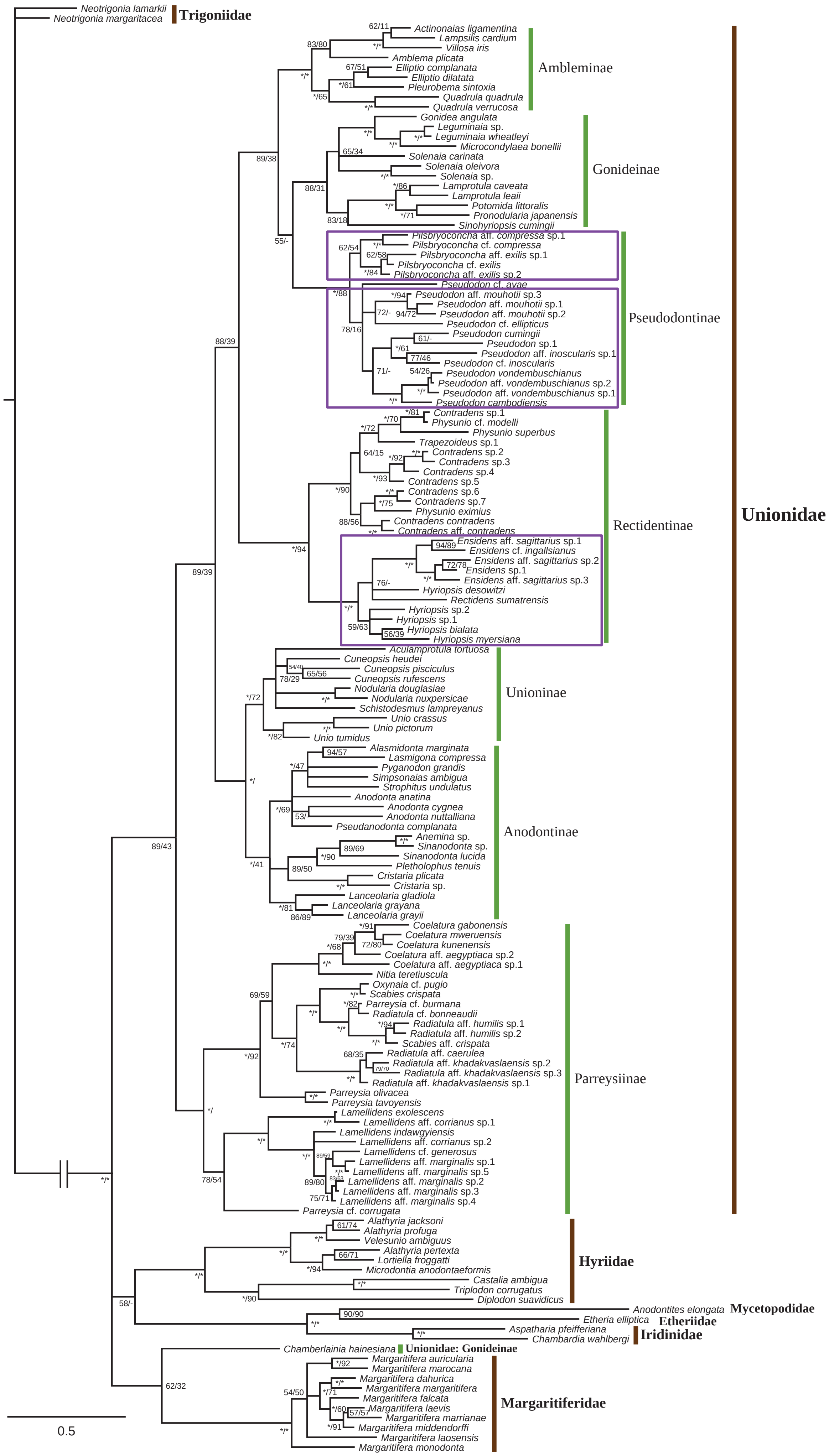

Supplementary Figure 2. Majority rule consensus phylogenetic tree of the Unionidae recovered from BI analysis of the mtDNA data set (four partitions: three codons of COI + 16S rRNA). Numbers near branches are the Bayesian posterior probability/ML bootstrap support values (an asterisk indicates value of ≥95%; values <50% are omitted; "-" indicates topological difference). The violet frames indicate the two ancient bivalve radiations within the Paleo-Mekong drainage basin.

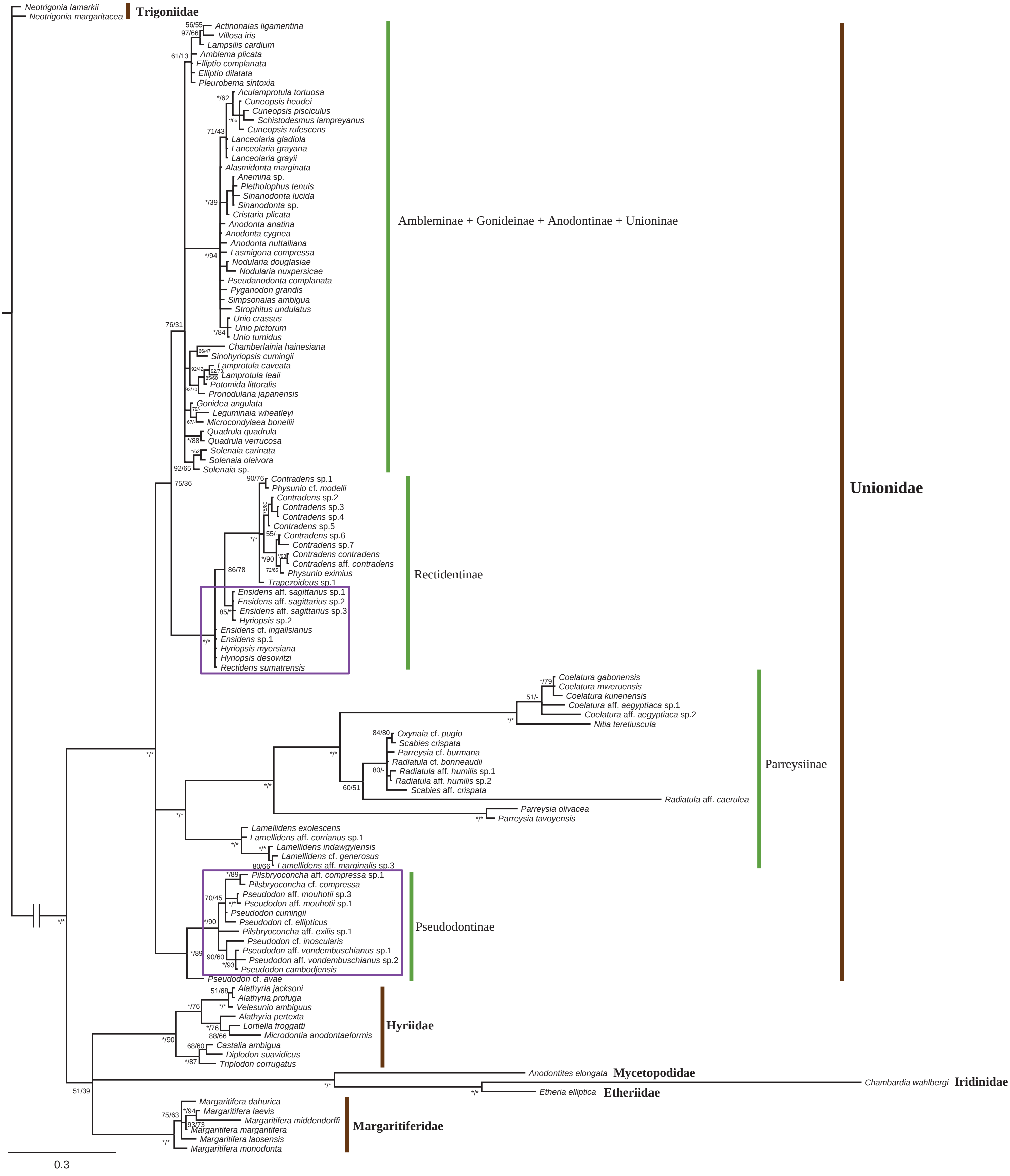

Supplementary Figure 3. Majority rule consensus phylogenetic tree of the Unionidae recovered from BI analysis of the nuclear 28S rDNA data set. Numbers near branches are the Bayesian posterior probability/ML bootstrap support values (an asterisk indicates value of ≥95%; values <50% are omitted; "-" indicates topological difference). The violet frames indicate the two ancient bivalve radiations within the Paleo-Mekong drainage basin.

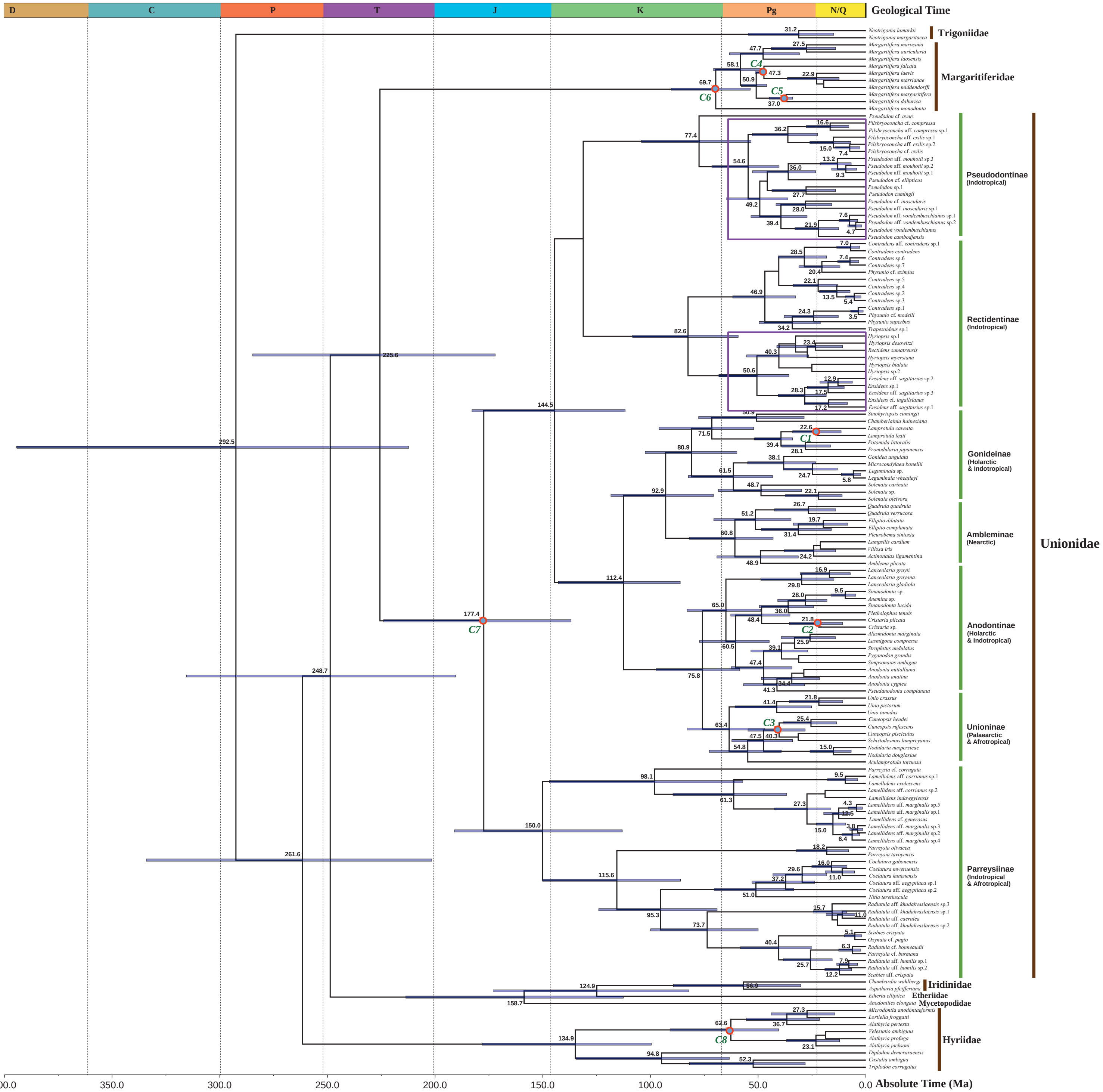

Supplementary Figure 4. Fossil-calibrated ultrametric chronogram calculated under a lognormal relaxed clock model and a Yule process speciation implemented in BEAST 1.8.3 and obtained for the complete data set of mitochondrial and nuclear sequences (five partitions: three codons of COI + 16S rRNA + 28S rDNA). The violet frames indicate the two ancient bivalve radiations within the Paleo-Mekong drainage basin. Blue circles indicate eight fossil calibrations (C1–C8, see Supplementary Table 12 for details). Black numbers near nodes are the mean age values, and bars are 95% confidence intervals of the estimated divergence time between lineages (Ma). The timing of doubtful nodes, the position of which differs in different analyses, is omitted. Stratigraphic chart according to the International Commission on Stratigraphy, 2015. The list of sequences is presented in Supplementary Table 1.

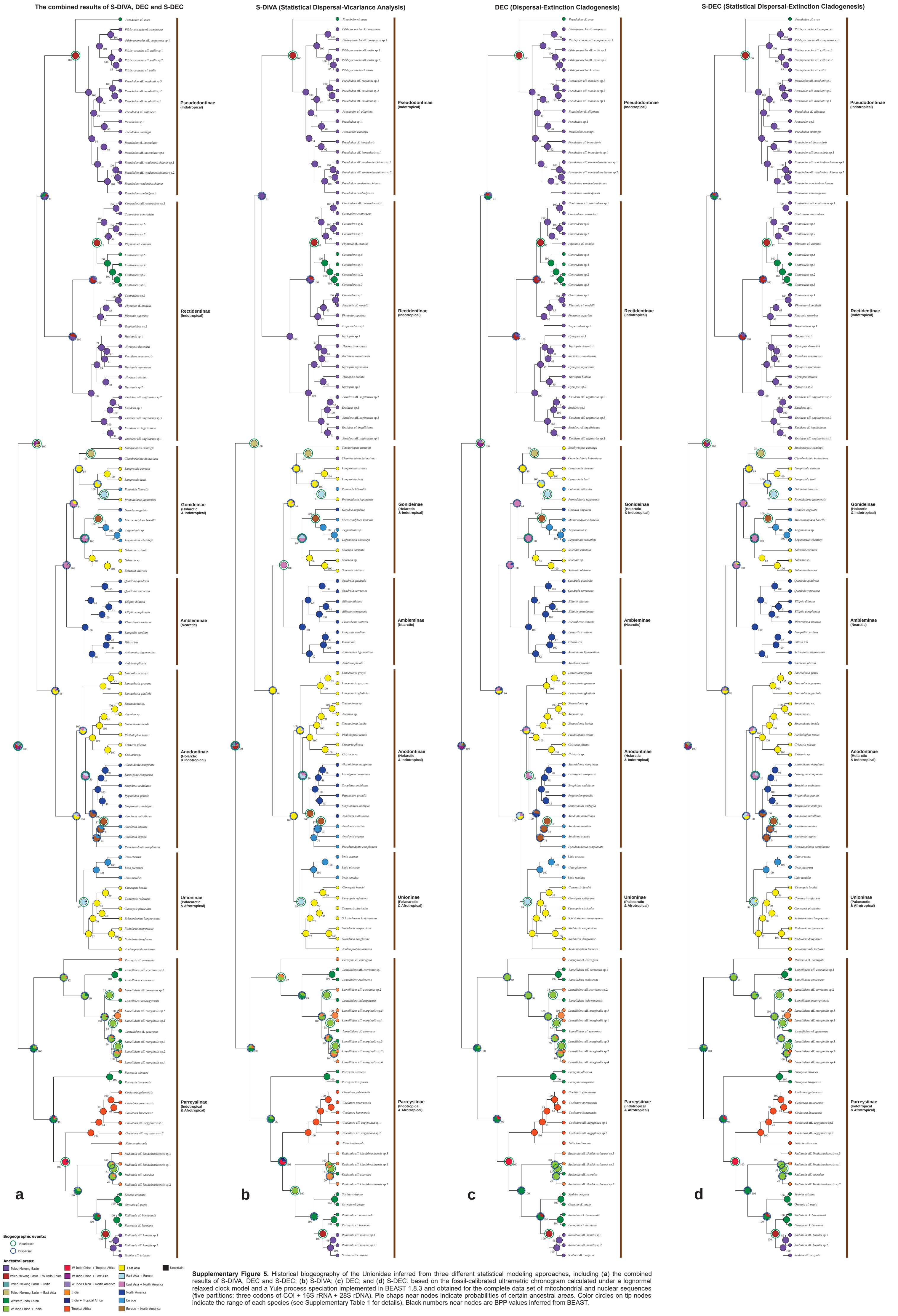

Supplementary Figure 5. Historical biogeography of the Unionidae inferred from three different statistical modeling approaches, including (**a**) the combined results of S-DIVA, DEC and S-DEC; (**b**) S-DIVA; (**c**) DEC; and (**d**) S-DEC. based on the fossil-calibrated ultrametric chronogram calculated under a lognormal relaxed clock model and a Yule process speciation implemented in BEAST 1.8.3 and obtained for the complete data set of mitochondrial and nuclear sequences (five partitions: three codons of COI + 16S rRNA + 28S rDNA). Pie chaps near nodes indicate probabilities of certain ancestral areas. Color circles on tip nodes indicate the range of each species (see Supplementary Table 1 for details). Black numbers near nodes are BPP values inferred from BEAST.

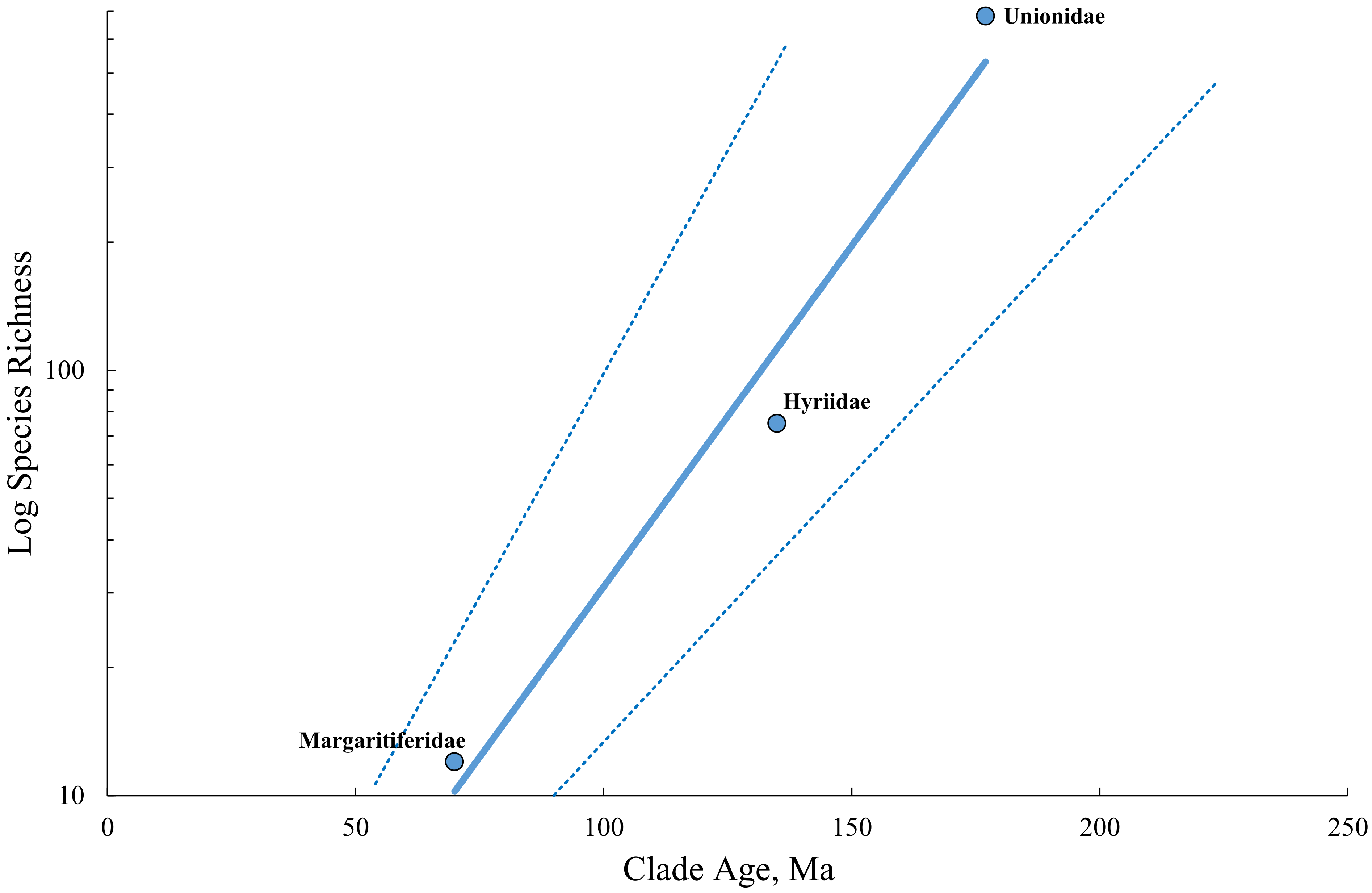

Supplementary Figure 6. Plot of log species richness vs. clade age for selected families of the Unionoida. The solid line is an exponential regression line ($y = 0.771e^{0.037x}$; $R^2 = 0.97$). Dashed lines are 95% confidence intervals of a regression based on 95% HPD of clade age. The species richness is given according to Graf (2013) and Bolotov et al. (2016).

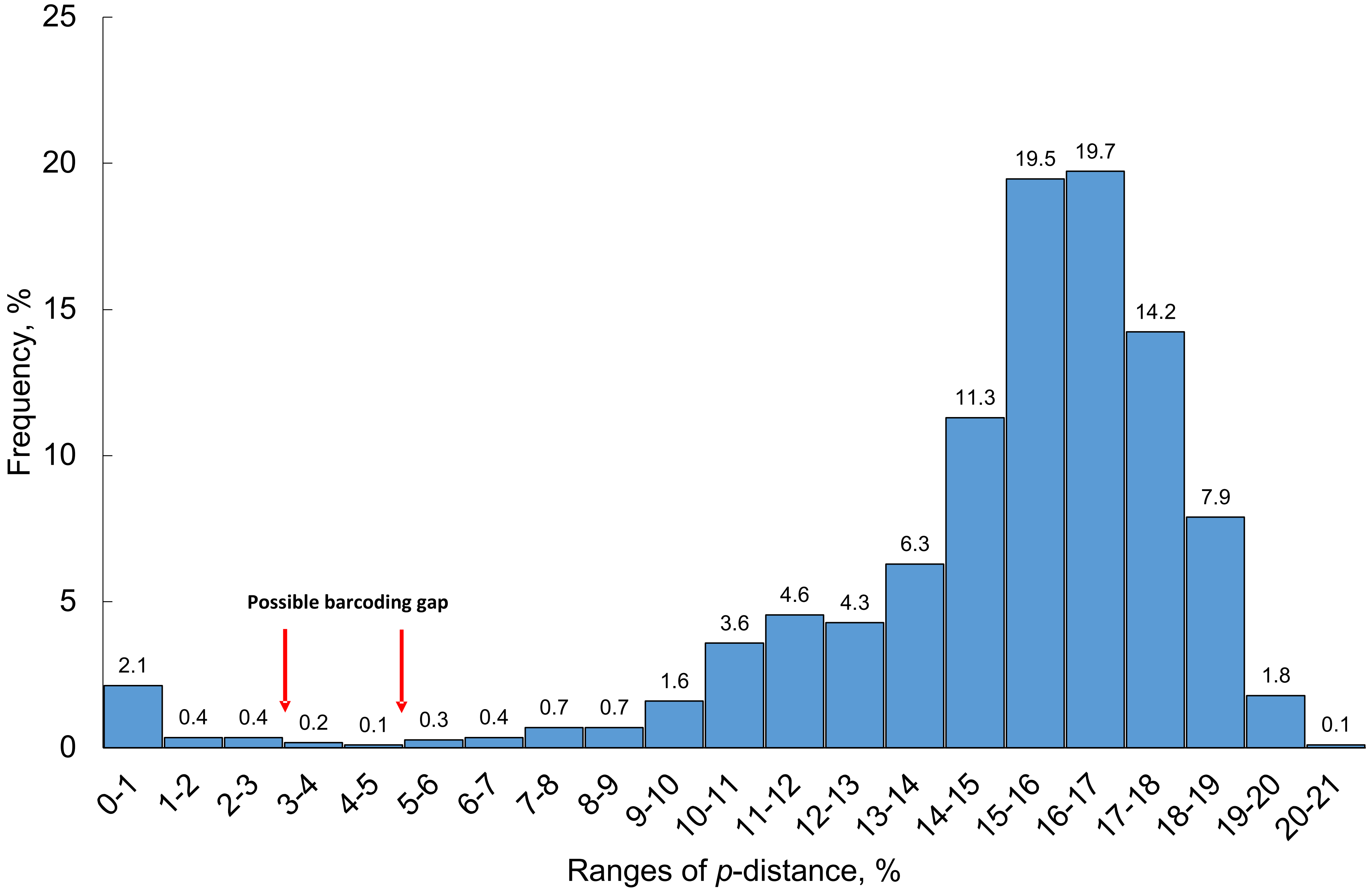

Supplementary Figure 7. Barcoding gap analyses for the Unionidae from the Oriental Region and related African taxa based on available COI sequences (*N* = 287). The list of sequences is presented in Supplementary Table 1.

References

1. Brooks, J. L. Speciation in ancient lakes (concluded). *The Quarterly Review of Biology* **25,** 131–176 (1950).
2. Martens, K., Coulter, G. & Goddeeris, B. Speciation in ancient lakes - 40 years after Brooks. *Ergebnisse der Limnologie* **44,** 75–96 (1994).
3. Martens, K. Speciation in ancient lakes. *Trends in Ecology & Evolution* **12,** 177–182 (1997).
4. Sherbakov, D. Y. Molecular phylogenetic studies on the origin of biodiversity in Lake Baikal, *Trends in Ecology & Evolution* 14, 92–95 (1999).
5. Von Rintelen, T., Wilson, A. B., Meyer, A. & Glaubrecht, M. Escalation and trophic specialization drive adaptive radiation of freshwater gastropods in ancient lakes on Sulawesi, Indonesia. *Proceedings of the Royal Society of London B: Biological Sciences* **271,** 2541–2549 (2004).
6. Wilson, A. B., Glaubrecht, M. & Meyer, A. Ancient lakes as evolutionary reservoirs: evidence from the thalassoid gastropods of Lake Tanganyika. *Proceedings of the Royal Society of London B: Biological Sciences* **271,** 529–536 (2004).
7. Vanhove, M. P. *et al.* Hidden biodiversity in an ancient lake: phylogenetic congruence between Lake Tanganyika tropheine cichlids and their monogenean flatworm parasites. *Scientific Reports* **5,** 13669 (2015).
8. Albrecht, C., Trajanovski, S., Kuhn, K., Streit, B. & Wilke, T. Rapid evolution of an ancient lake species flock: freshwater limpets (Gastropoda: Ancylidae) in the Balkan Lake Ohrid. *Organisms Diversity & Evolution* **6,** 294–307 (2006).
9. Albrecht, C. & Wilke, T. Ancient Lake Ohrid: biodiversity and evolution. In *Patterns and Processes of Speciation in Ancient Lakes*. 103–140 (Springer Netherlands, 2008).
10. Von Rintelen, T., von Rintelen, K., Glaubrecht, M., Schubart, C. D. & Herder, F. Aquatic biodiversity hotspots in Wallacea: the species flocks in the ancient lakes of Sulawesi, Indonesia. In *Biotic evolution and environmental change in Southeast Asia*. 290–315 (Cambridge University Press, Cambridge, 2012).
11. Ivory, S. J. *et al.* Environmental change explains cichlid adaptive radiation at Lake Malawi over the past 1.2 million years. *Proceedings of the National Academy of Sciences* **113,** 11895–11900 (2016).
12. Vannote, R. L., Minshall, G. W., Cummins, K. W., Sedell, J. R. & Cushing CE. The river continuum concept. *Canadian Journal of Fisheries and Aquatic Sciences* **37,** 130–137 (1980).
13. Junk, W. J., Bayley, P. B. & Sparks, R. E. The flood pulse concept in river-floodplain systems. *Canadian Special Publication of Fisheries and Aquatic Sciences* **106,** 110–127 (1989).
14. Hooke, J. M. Spatial variability, mechanisms and propagation of change in an active meandering river. *Geomorphology* **84,** 277–296 (2007).
15. Grande, L. & Bemis, W. E. Osteology and phylogenetic relationships of fossil and recent paddlefishes (Polyodontidae) with comments on the interrelationships of Acipenseriformes. *Journal of Vertebrate Paleontology* **11,** 1–21 (1991).
16. Bemis, W. E., Kynard, B. Sturgeon rivers: an introduction to acipenseriform biogeography and life history. *Environmental Biology of Fishes* **48,** 167–183 (1997).

17. Glaubrecht, M. & Köhler, F. Radiating in a river: systematics, molecular genetics and morphological differentiation of viviparous freshwater gastropods endemic to the Kaek River, central Thailand (Cerithioidea, Pachychilidae). *Biological Journal of the Linnean Society* **82,** 275–311 (2004).
18. Hershler, R., Liu, H. P. & Sada, D. W. Origin and diversification of the Soldier Meadow springsnails (Hydrobiidae: Pyrgulopsis), a species flock in the northwestern Great Basin, United States. *Journal of Molluscan Studies* **73,** 167–183 (2007).
19. Köhler, F., Panha, S. & Glaubrecht, M. Speciation and radiation in a river: assessing the morphological and genetic differentiation in a species flock of viviparous gastropods (Cerithioidea: Pachychilidae). In *Evolution in Action*. 513–550 (Springer Berlin Heidelberg, 2010).
20. Köhler, F. & Glaubrecht, M. Uncovering an overlooked radiation: Morphological and mitochondrial DNA differentiation in endemic freshwater snails on Madagascar (Caenogastropoda: Pachychilidae) and their biogeography. *Biological Journal of the Linnean Society* **99,** 867–894 (2010).
21. Glaubrecht, M. Towards Solving Darwin's "mystery": Speciation and Radiation in Lacustrine and Riverine Freshwater Gastropods. *American Malacological Bulletin* **29,** 187–216 (2011).
22. De Bruyn, M. *et al.* Paleo-drainage basin connectivity predicts evolutionary relationships across three Southeast Asian biodiversity hotspots. *Systematic Biology* **62,** 398–410 (2013).
23. Wesselingh, F. P. Long-lived lake molluscs as island faunas: a bivalve perspective. In *Biogeography, time, and place: distributions, barriers, and islands*. 275–314 (Springer Netherlands, 2007).
24. Bogan, A. E. & Roe, K. J. Freshwater bivalve (Unioniformes) diversity, systematics, and evolution: status and future directions. *Journal of the North American Benthological Society* **27,** 349–369 (2008).
25. Graf, D. L. Patterns of freshwater bivalve global diversity and the state of phylogenetic studies on the Unionoida, Sphaeriidae, and Cyrenidae. *American Malacological Bulletin* **31,** 135–153 (2013).
26. Graf, D. L. & Cummings, K. S. Review of the systematics and global diversity of freshwater mussel species (Bivalvia: Unionoida). *Journal of Molluscan Studies* **73,** 291–314 (2007).
27. Lopes-Lima, M. *et al.* Phylogeny of the most species-rich freshwater bivalve family (Bivalvia: Unionida: Unionidae): Defining modern subfamilies and tribes. *Molecular Phylogenetics and Evolution* **106,** 174–191 (2017).
28. Voris, H. K. Maps of Pleistocene sea levels in Southeast Asia: shorelines, river systems and time durations. *Journal of Biogeography* **27,** 1153–1167 (2000).
29. De Bruyn, M., Nugroho, E., Hossain, M. M., Wilson, J. C, Mather, P. B. Phylogeographic evidence for the existence of an ancient biogeographic barrier: the Isthmus of Kra Seaway. *Heredity* **94,** 370–378 (2005).
30. De Bruyn, M. *et al.* Borneo and Indochina are major evolutionary hotspots for Southeast Asian biodiversity. *Systematic Biology* **63,** 879–901 (2014).
31. Lohman, D. J. et al. Biogeography of the Indo-Australian archipelago. *Annual Review of Ecology, Evolution, and Systematics* **42,** 205–226 (2011).
32. Graf, D. L., Geneva, A. J., Pfeiffer, J. M. & Chilala, A. D. Phylogenetic analysis of *Prisodontopsis* Tomlin, 1928 and *Mweruella* Haas, 1936 (Bivalvia: Unionidae) from Lake

Mweru (Congo basin) supports a Quaternary radiation in the Zambian Congo. *Journal of Molluscan Studies* **80,** 303–314 (2014).
33. Scholz, H. & Glaubrecht, M. Evaluating limnic diversity: Toward a revision of the unionid bivalve *Coelatura* Conrad, 1853 in the Great Lakes of East Africa and adjacent drainage systems (Mollusca, Bivalvia, Unionidae). *Zoosystematics and Evolution* **80,** 89–121 (2004).
34. Banarescu, P. *Zoogeography of fresh waters. Volume 2: Distribution and dispersal of freshwater animals in North America and Eurasia*. 519–1091 (1991).
35. Starobogatov, Y. I. *Fauna Mollyuskov I Zoogeograficheskoe Raionirovanie Kontinental'nykh Vodoemov Zemnogo Shara [Mollusk Fauna and Zoogeographical Partitioning of Continental Water Reservoirs of the World]*. (Nauka, Leningrad, 1970) (In Russian).
36. Bolotov, I. N. *et al.* Multi-locus fossil-calibrated phylogeny, biogeography and a subgeneric revision of the Margaritiferidae (Mollusca: Bivalvia: Unionoida). *Molecular Phylogenetics and Evolution* **103,** 104–121 (2016).
37. Whelan, N. V., Geneva, A. J. & Graf, D. L. Molecular phylogenetic analysis of tropical freshwater mussels (Mollusca: Bivalvia: Unionoida) resolves the position of *Coelatura* and supports a monophyletic Unionidae. *Molecular Phylogenetics and Evolution* **61,** 504–514 (2011).
38. Köhler, F. *et al.* The status and distribution of freshwater molluscs of the Indo-Burma region. In *The status and distribution of freshwater biodiversity in Indo-Burma*, 66–88 (2012).
39. Konopleva, E. S., Bolotov, I. N., Vikhrev, I. V., Gofarov, M. Y. & Kondakov, A. V. An integrative approach underscores the taxonomic status of *Lamellidens exolescens*, a freshwater mussel from the Oriental tropics (Bivalvia: Unionidae). *Systematics and Biodiversity* **15**, 204–217 (2017).
40. Pfeiffer, J. M. III & Graf. D. L. Evolution of bilaterally asymmetrical larvae in freshwater mussels (Bivalvia: Unionoida: Unionidae). *Zoological Journal of the Linnean Society* **175,** 307–318 (2015).
41. Zieritz, A. *et al.* Factors driving changes in freshwater mussel (Bivalvia, Unionida) diversity and distribution in Peninsular Malaysia. *Science of the Total Environment* **571,** 1069–1078 (2016).
42. Zieritz, A. *et al.* Diversity, biogeography and conservation of freshwater mussels (Bivalvia: Unionida) in East and Southeast Asia. *Hydrobiologia*, doi: 10.1007/s10750-017-3104-8 (2017).
43. Brandt, R. A. M. The non-marine aquatic Mollusca of Thailand. *Archiv fur Molluskenkunde* **105,** 1–423 (1974).
44. Subba Rao, N. V. *Handbook freshwater molluscs of India*. (Zoological Survey of India, Calcutta, 1989).
45. Nesemann, H. A., Sharma, S. U., Sharma, G. O. & Sinha, R. K. Illustrated checklist of large freshwater bivalves of the Ganga river system (Mollusca: Bivalvia: Solecurtidae, Unionidae, Amblemidae). *Nachrichchtenblatt der Ersten Vorarlberger Malakologischen Gesellschaft* **13,** 1–51 (2005).
46. Bieler, R. *et al.* Investigating the Bivalve Tree of Life – an exemplar-based approach combining molecular and novel morphological characters. *Invertebrate Systematics* **28,** 32–115 (2014).
47. Graf, D. L., Jones, H., Geneva, A. J., Pfeiffer, J. M. & Klunzinger, M. W. Molecular phylogenetic analysis supports a Gondwanan origin of the Hyriidae (Mollusca: Bivalvia:

Unionida) and the paraphyly of Australasian taxa. *Molecular Phylogenetics and Evolution* **85,** 1–9 (2015).

48. McPeek, M. A. & Brown, J. M. Clade age and not diversification rate explains species richness among animal taxa. *The American Naturalist* **169,** E97–E106 (2007).
49. Rabosky, D. L. Ecological limits and diversification rate: alternative paradigms to explain the variation in species richness among clades and regions. *Ecology Letters* **12,** 735–743 (2009).
50. Fang, Z. J., Chen, J. H., Chen, C. Z., Sha, J. G., Lan, X. & Wen, S. X. Supraspecific taxa of the Bivalvia first named, described, and published in China (1927–2007). *University of Kansas Paleontological Contributions: New Series* **17,** 1–157 (2009).
51. Pan, Y. H. & Sha, J. G. Middle Jurassic unionids (non-marine Bivalvia) from the Shiwandashan Basin, southern China, with special emphasis on *Cuneopsis* Simpson. *GFF* **131,** 183–194 (2009).
52. Skawina, A. & Dzik, J. Umbonal musculature and relationships of the Late Triassic filibranch unionoid bivalves. *Zoological Journal of the Linnean Society* **163,** 863–883 (2011).
53. Schneider, S., Böhme, M. & Prieto, J. Unionidae (Bivalvia; Palaeoheterodonta) from the Palaeogene of northern Vietnam: exploring the origins of the modern East Asian freshwater bivalve fauna. *Journal of Systematic Palaeontology* **11,** 337–357 (2013).
54. Jordan, S. M., Barraclough, T. G. & Rosindell, J. Quantifying the effects of the break up of Pangaea on global terrestrial diversification with neutral theory. *Philosophical Transactions of the Royal Society B: Biological Sciences* **371,** 20150221 (2016).
55. Wilkinson, M., Sheps, J. A., Oommen, O. V. & Cohen, B. L. Phylogenetic relationships of Indian caecilians (Amphibia: Gymnophiona) inferred from mitochondrial rRNA gene sequences. *Molecular Phylogenetics and Evolution* **23,** 401–407 (2002).
56. Biju, S.D. & Bossuyt, F. New frog family from India reveals an ancient biogeographical link with the Seychelles. *Nature* **425,** 711–713 (2003).
57. Bossuyt, F. & Milinkovitch, M. C. Amphibians as indicators of Early Tertiary “Out-of-India” dispersal of vertebrates. *Science* **292,** 93–95 (2001).
58. Klaus, S., Schubart, C. D., Streit, B. & Pfenninger, M. When Indian crabs were not yet Asian-biogeographic evidence for Eocene proximity of India and Southeast Asia. *BMC Evolutionary Biology* **10,** 287 (2010).
59. Graf, D. L. & Cummings, K. S. Actual and alleged freshwater mussels (Mollusca: Bivalvia: Unionoida) from Madagascar and the Mascarenes, with description of a new genus, *Germainaia*. *Proceedings of the Academy of Natural Sciences of Philadelphia* **158,** 221–238 (2009).
60. Graf, D. L. & Cummings, K. S. Freshwater mussel (Mollusca: Bivalvia: Unionoida) richness and endemism in the ecoregions of Africa and Madagascar based on comprehensive museum sampling. *Hydrobiologia* **678,** 17–36 (2011).
61. Ali, J. R. & Aitchison, J. C. Gondwana to Asia: plate tectonics, paleogeography and the biological connectivity of the Indian sub-continent from the Middle Jurassic through latest Eocene (166–35 Ma). *Earth-Science Reviews* **88,** 145–166 (2008).
62. Briggs, J. C. The biogeographic and tectonic history of India. *Journal of Biogeography* **30,** 381–388 (2003).

63. Köhler, F. & Glaubrecht, M. Out of Asia and into India: on the molecular phylogeny and biogeography of the endemic freshwater gastropod Paracrostoma Cossmann, 1900 (Caenogastropoda: Pachychilidae). *Biological Journal of the Linnean Society* **91,** 627–651 (2007).
64. Froufe, E. *et al.* Who lives where? Molecular and morphometric analyses clarify which Unio species (Unionida, Mollusca) inhabit the southwestern Palearctic. *Organisms Diversity & Evolution* **16,** 597–611 (2016).
65. Mock, K. E. *et al.* Genetic structuring in the freshwater mussel Anodonta corresponds with major hydrologic basins in the western United States. *Molecular Ecology* **19,** 569–591 (2010).
66. Tamura, K., Stecher, G., Peterson, D., Filipski, A. & Kumar, S., MEGA6: Molecular Evolutionary Genetics Analysis version 6.0. *Molecular Biology and Evolution* **30,** 2725–2729 (2013).
67. Talavera, G. & Castresana, J. Improvement of phylogenies after removing divergent and ambiguously aligned blocks from protein sequence alignments. *Systematic Biology* **56,** 564–577 (2007).
68. Xia, X., Xie, Z., Salemi, M., Chen, L. & Wang Y. An index of substitution saturation and its application. *Molecular Phylogenetics and Evolution* **26,** 1–7 (2003).
69. Xia, X. & Lemey, P. Assessing substitution saturation with DAMBE. In *The Phylogenetic Handbook: A Practical Approach to DNA and Protein Phylogeny, Second Edition* (Lemey, P., Salemi, M. & Vandamme, A., eds.) 615–630 (Cambridge University Press, 2009).
70. Swofford, D.L. *PAUP*. Phylogenetic Analysis Using Parsimony (*and Other Methods). Version 4.0b10.* (Sinauer Associates, Sunderland, Massachusetts, 2002).
71. Blaxter, M. *et al.* Defining operational taxonomic units using DNA barcode data. *Philosophical Transactions of the Royal Society B: Biological Sciences* **360,** 1935–1943 (2005).
72. De Queiroz, K. Species concepts and species delimitation. *Systematic Biology* **56,** 879–886 (2007).
73. Jones, M., Ghoorah, A. & Blaxter, M. jMOTU and Taxonerator: Turning DNA barcode sequences into annotated operational taxonomic units. *PLoS ONE* **6,** e19259 (2011).
74. Wiens, J. J. Species delimitation: new approaches for discovering diversity. *Systematic Biology* **56,** 875–878 (2007).
75. Zhang, J., Kapli, P., Pavlidis, P. & Stamatakis, A. A general species delimitation method with applications to phylogenetic placements. *Bioinformatics* **29,** 2869–2876 (2013).
76. Stamatakis, A. RAxML-VI-HPC: maximum likelihood-based phylogenetic analyses with thousands of taxa and mixed models. *Bioinformatics* **22,** 2688–2690 (2006).
77. Miller, M., Pfeiffer, W. & Schwartz, T. Creating the CIPRES Science Gateway for inference of large phylogenetic trees. In *Gateway Computing Environments Workshop* (GCE). 1–8 (IEEE, 2010).
78. Prié, V. & Puillandre, N. Molecular phylogeny, taxonomy, and distribution of French *Unio* species (Bivalvia, Unionidae). *Hydrobiologia* **735,** 95–110 (2014).
79. Bolotov, I. N. *et al.* Origin of a divergent mtDNA lineage of a freshwater snail species, *Radix balthica*, in Iceland: cryptic glacial refugia or a postglacial founder event? *Hydrobiologia* **787,** 73–98 (2017).

80. Fehér, Z., Major, Á. & Krízsik, V. Spatial pattern of intraspecific mitochondrial diversity in the Northern Carpathian endemic spring snail, *Bythinella pannonica* (Frauenfeld, 1865) (Gastropoda: Hydrobiidae). *Organisms Diversity & Evolution* **13,** 569–581 (2013).
81. Villesen, P. FaBox: an online toolbox for fasta sequences. *Molecular Ecology Notes* **7,** 965–968 (2007).
82. Stamatakis, A., Hoover, P. & Rougemont, J. A rapid bootstrap algorithm for the RAxML web servers. *Systematic Biology* **57,** 758–771 (2008).
83. Ronquist, F. *et al.* MrBayes 3.2: Efficient Bayesian Phylogenetic Inference and Model Choice Across a Large Model Space. *Systematic Biology* **61,** 539–542 (2012).
84. Rambaut, A., Suchard, M. & Drummond, A. J. Tracer v1.6. Available: http://beast.bio.ed.ac.uk/software/tracer/ (2013).
85. Drummond, A. J., Ho, S. Y., Phillips, M. J. & Rambaut, A. Relaxed phylogenetics and dating with confidence. *PLoS Biology* **4,** 699 (2006).
86. Drummond, A.J. & Rambaut, A. BEAST: Bayesian evolutionary analysis by sampling trees. *BMC Evolutionary Biology* **7,** 214. (2007).
87. Drummond, A. J., Suchard, M. A., Xie, D. & Rambaut, A. Bayesian phylogenetics with BEAUti and the BEAST 1.7. *Molecular Biology and Evolution* **29,** 1969–1973 (2012).
88. Marshall, C. R. A simple method for bracketing absolute divergence times on molecular phylogenies using multiple fossil calibration points. *The American Naturalist* **171,** 726–742 (2008).
89. Delvene, G. & Araujo, R. Early Cretaceous non-marine bivalves from the Cameros and Basque-Cantabrian basins of Spain. *Journal of Iberian Geology* **35,** 19–34 (2009).
90. Yu, Y., Harris, A.J., Blair, C. & He, X.J. RASP (Reconstruct Ancestral State in Phylogenies): a tool for historical biogeography. *Molecular Phylogenetics and Evolution* **87,** 46–49 (2015).
91. Paradis, E. *Analysis of Phylogenetics and Evolution with R (Second Edition)*. (New York, Springer, 2012).
92. Popescu, A. A., Huber, K. T. & Paradis, E. APE 3.0: new tools for distance based phylogenetics and evolutionary analysis in R. *Bioinformatics* **28,** 1536–1537 (2012).
93. Bapst, D. W. Paleotree: an R package for paleontological and phylogenetic analyses of evolution. *Methods in Ecology and Evolution* **3,** 803–807 (2012).
94. Pybus, O. G. & Harvey P. H. Testing macro-evolutionary models using incomplete molecular phylogenies. *Proceedings of the Royal Society of London. Series B. Biological Sciences* **267,** 2267–2272 (2000).
95. Paradis, E. Assessing temporal variations in diversification rates from phylogenies: estimation and hypothesis testing. *Proceedings of the Royal Society of London. Series B. Biological Sciences* **264,** 1141–1147 (1997).
96. Graf, D. L. Molecular phylogenetic analysis of two problematic freshwater mussel genera (*Unio* and *Gonidea*) and a re-evaluation of the classification of Nearctic Unionidae (Bivalvia: Palaeoheterodonta: Unionoida). *Journal of Molluscan Studies* **68,** 65–71 (2002).
97. Graf, D. L. & Cummings, K. S. Palaeoheterodont diversity (Mollusca: Trigonioida+ Unionoida): what we know and what we wish we knew about freshwater mussel evolution. *Zoological Journal of the Linnean Society* **148,** 343–394 (2006).

98. Sharma, P. P. *et al.* Into the deep: a phylogenetic approach to the bivalve subclass Protobranchia. *Molecular Phylogenetics and Evolution* **69,** 188–204 (2013).

99. Graf, D. L. & Foighil, D. Ó. The evolution of brooding characters among the freshwater pearly mussels (Bivalvia: Unionoidea) of North America. *Journal of Molluscan Studies* **66,** 157–170 (2000).

100. Bogan, A. E. & Hoeh, W. R. On becoming cemented: evolutionary relationships among the genera in the freshwater bivalve family Etheriidae (Bivalvia: Unionoida). *Geological Society of London, Special Publications* **177,** 159–168 (2000).

101. Froufe, E. *et al.* Phylogeny, phylogeography, and evolution in the Mediterranean region: News from a freshwater mussel (*Potomida*, Unionida). *Molecular Phylogenetics and Evolution* **100,** 322–332 (2016).

102. Gustafson, R. G. & Iwamoto, E. M. A DNA-based identification key to Pacific Northwest freshwater mussel glochidia: importance to salmonid and mussel conservation. *Northwest Science* **79,** 233–245 (2005).

103. Hoeh, W. R. *et al.* Testing altenative hypotheses of *Neotrigonia* (Bivalvia: Trigonioida) phylogenetic relationships using cytochrome c oxidase subunit I DNA sequences. *Malacologia* **40,** 267–278 (1998).

104. Giribet, G. & Wheeler, W. On bivalve phylogeny: a high-level analysis of the Bivalvia (Mollusca) based on combined morphology and DNA sequence data. *Invertebrate Biology* **121,** 271–324 (2002).

105. Distel, D. L. *et al.* Molecular phylogeny of Pholadoidea Lamarck, 1809 supports a single origin for xylotrophy (wood feeding) and xylotrophic bacterial endosymbiosis in Bivalvia. *Molecular Phylogenetics and Evolution* **61,** 245–254 (2011).

106. Huff, S.W. *et al.* Investigations into the phylogenetic relationships of freshwater pearl mussels (Bivalvia: Margaritiferidae) based on molecular data: implications for their taxonomy and biogeography. *Journal of Molluscan Studies* **70,** 379–388 (2004).

107. Araújo, R., Toledo, C., Van Damme, D., Ghamizi, M. & Machordom, A. *Margaritifera marocana* (Pallary, 1918): a valid species inhabiting Moroccan rivers. *Journal of Molluscan Studies* **75,** 95–101 (2009).

108. Breton, S. *et al.* Novel protein genes in animal mtDNA: a new sex determination system in freshwater mussels (Bivalvia: Unionoida)? *Molecular Biology and Evolution* **28,** 1645–1659 (2011).

109. González, V. L. & Giribet, G. A multilocus phylogeny of archiheterodont bivalves (Mollusca, Bivalvia, Archiheterodonta). *Zoologica Scripta* **44,** 41–58 (2015).

110. Giribet, G. *et al.* Evidence for a clade composed of molluscs with serially repeated structures: monoplacophorans are related to chitons. *Proceedings of the National Academy of Sciences* **103,** 7723–7728 (2006).

111. Lobo, J. *et al.* Enhanced primers for amplification of DNA barcodes from a broad range of marine metazoans. *BMC Ecology* 13, 34 (2013).

112. Palumbi, S.R. Nucleic acids II: The polymerase chain reaction. in *Molecular Systematics* (Hillis D.M., Moritz C., Mable B.K., eds.). 205–247 (Sinauer Associates Inc., Sunderland, MA, 1996).

113. Lydeard, C., Mulvey, M., Davis, G. M. Molecular systematics and evolution of reproductive traits of North American freshwater unionacean mussels (Mollusca: Bivalvia) as inferred from 16S rRNA gene sequences. *Philos. T. Roy. Soc. B.* **351,** 1593–1603 (1996).

114. Jovelin, R. & Justine, J. L. Phylogenetic relationships within the polyopisthocotylean monogeneans (Platyhelminthes) inferred from partial 28S rDNA sequences. *Int. J. Parasitol.* **31,** 393–401 (2001).

115. Takayasu, K., Gurung, D. D., Matsuoka, K. Some new species of freshwater bivalves from the Mio-Pliocene Churia Group, west-central Nepal. *Trans. Proc. Palaeont. Soc. Japan N.S.* **179,** 157–168 (1995).
